# Mechanochemically functionalized waste plastics for NO_2_ sensing

**DOI:** 10.1038/s41467-026-72492-8

**Published:** 2026-04-25

**Authors:** Yingnan Zhao, Liang Pang, Zhao Zhao, Hang Shi, Renquan Guan, Zhongling Lang, Ruiqi Yao, Tonghui Wang, Zhimin Ao, Huaqiao Tan, Gao-Feng Han, Xing-You Lang, Qing Jiang

**Affiliations:** 1 Key Laboratory of Automobile Materials (Jilin University), Ministry of Education, and School of Materials Science and Engineering, Jilin University, Changchun, P.R. China; 2 Key Laboratory of Polyoxometalate and Reticular Material Chemistry of Ministry of Education, and Faculty of Chemistry, Northeast Normal University, Changchun, P.R. China; 3 Guangdong Provincial Key Laboratory of Wastewater Information Analysis and Early Warning, Advanced Interdisciplinary Institute of Environment and Ecology, School of Technology for Sustainability, Beijing Normal University, Zhuhai, P.R. China

**Keywords:** Sensors and biosensors, Heterogeneous catalysis

## Abstract

Low-entropy non-depolymerizing upcycling of waste plastics into value-added products is promising, as it can avoid energy-intensive depolymerization process. Here, we report a mechanochemical approach for the direct functionalization of plastics with polyoxometalate (POM) molecules. The resulting POM-plastic composites retain integrated plastic chains and exhibit strong potential for NO_2_ sensing. Among the composites, phosphotungstic acid/polyethylene terephthalate (PW_12_-PET) achieves a low limit of detection (10.52 ppb) and fast response/recovery times (19.2 s/13.5 s at 5.0 ppm), and shows practical applicability after device assembly. Moreover, it exhibits high selectivity against ten interfering gases. Mechanistic studies reveal that PET transfers multiple charges to PW_12_. It not only activates new bridge-oxygen sites on PW_12_ but also maintains a moderate adsorption strength, enabling rapid NO_2_ response. This work represents a promising example of direct non-depolymerizing upcycling of waste plastics into value-added functional materials.

## Abstract (web summary)

Mechanochemical functionalization of waste plastics with polyoxometalate provides a sustainable method for low-entropy non-depolymerizing upcycling them into NO_2_ sensor.

## Introduction

**Fig. 1:**
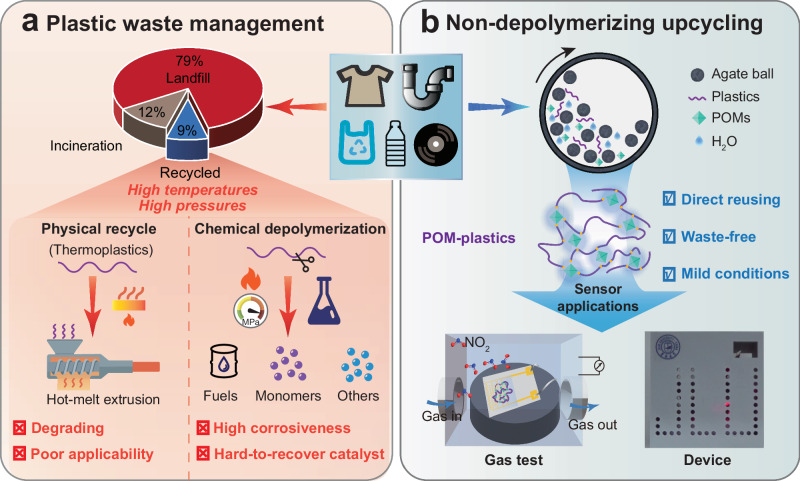
Schematic illustration of plastic recycling strategies. **a** Plastic waste management recycles only approximately 9% of waste and typically requires harsh processing conditions. Physical recycling, primarily used for thermoplastics, involves melt reprocessing, whereas chemical recycling requires depolymerization of plastics into monomers or oligomers. **b** This work presents a non-depolymerizing upcycling strategy that directly converts waste plastics into high-value functional materials. POM-plastic composites are synthesized via a one-step mechanochemical ball-milling method and show great promise in NO_2_ gas sensing.

Plastics containing various functional groups—such as esters, phenyls, amides, or halogens (–F or –Cl)—are widely used in daily life and industry^1–3^. However, plastic waste management systems have failed to keep pace with the surge in plastic production^4^. Most discarded plastics are still incinerated or landfilled, leading to serious environmental concerns^5^. Consequently, plastic recycling has attracted significant interest (Fig. 1a)^6,7^. However, physical recycling often suffers from quality degradation due to chain scission and oxidation^8^. While chemical depolymerization into fuels, monomers, or other chemicals^9^, typically requires harsh reaction conditions, such as elevated temperatures (typically > 200 °C), high pressures, or the use of high corrosive acids or bases^10,11^. Meanwhile, they also face challenges in catalyst recovery and product purification^12^. These limitations raise a critical question: is it possible to develop atom-economical and environmentally friendly strategies that retain the polymer backbone while introducing high-value functional properties?

However, the chemical inertness of plastic surfaces presents a significant hindrance to their effective functionalization^13–15^. In this context, polyoxometalates (POMs) provide a unique solution to this challenge. Specifically, their molecular-level tunability enables them to function as both interfacial activators and sensing centers. Their strong Brønsted acidity and low corrosivity enable the selective activation of specific functional groups (such as C=O in esters) on inert plastic surfaces^16^, facilitating stable chemical anchoring through a synergistic network of hydrogen bonding and electrostatic interactions. Furthermore, the inherent ability of POM anions to undergo reversible multi-electron transfer without structural degradation significantly facilitates charge transfer during gas interaction. Crucially, the plastic functions as a functionalized platform that modulates the electronic environment and charge kinetics of the POM active sites^17,18^. This structure effectively addresses the inherent limitations of pure POMs (limited active site exposure), thereby transforming waste plastics into high-performance, active components for gas sensing.

Herein, a one-step mechanochemical strategy (Fig. 1b) was adopted to functionalize plastics with POMs via low-energy ball-milling. Driven by mechanical force, this process promotes intimate interfacial activation between POMs and plastics. Among various plastics, polyethylene terephthalate (PET) shows the strongest synergy with phosphotungstic acid (PW_12_), which exhibits the best NO_2_ sensing performance at room temperature. The optimized PW_12_-PET-1 sample achieves a low limit of detection (LOD) of 10.52 ppb, along with fast response and recovery times of 19.2 s and 13.5 s at 5.0 ppm NO_2_, respectively. It demonstrates an impressive combination of rapid response/recovery time and LOD. Additionally, it shows high selectivity against ten interfering gases and maintains operational stability. The assembled PW_12_-PET-1 smart device demonstrates practical potential for real-time NO_2_ monitoring. The significance of this composite, which proposes a synergistic mechanism rather than simple blending: ball-milling induces charge transfer from PET to PW_12_, thereby optimizing the surface electronic structure and boosting sensitivity. This work provides a green, economical strategy for upcycling waste plastics into high-performance functional materials, offering a perspective for sustainable waste management.

## Results and discussion

### Synthesis and structure characterization

As depicted in Fig. 1b, waste plastics were directly functionalized with POMs via a one-step mechanochemical method. To prevent depolymerization, we employed a low transmission ratio (1: −1) ball-milling device at a low rotation speed of 300 rpm. For instance, PW_12_, PET, and 5 ml of water were added into the milling jar, while the relative amounts of PET and PW_12_ were varied. In this process, water plays a crucial role. It prevents particle agglomeration and promotes complete dissolution of PW_12_, thereby facilitating effective proton activation and molecular-level functionalization of PET by polyoxoanions. After vacuum drying at 70 °C for 12 h, the resulting composites were named as PW_12_-PET-*x* (*x* = 1/4, 1/2, 1, 2, 4), where *x* denotes the molar ratio of H^+^ in PW_12_ to PET.

**Fig. 2:**
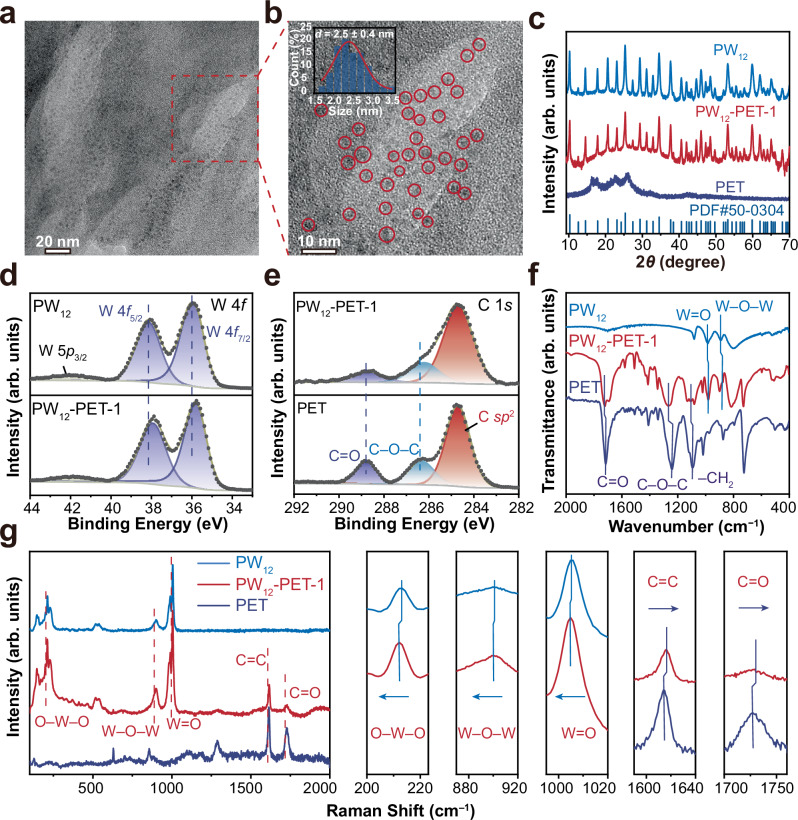
Structure and chemical state characterizations. **a** Cryo-TEM and **b** corresponding High-magnification Cryo-TEM images in the red circle of PW_12_-PET-1. The contrast of PW_12_ particles is significantly different compared to the PET substrate. The average particle size was statistically determined to be approximately 2.5 nm. The images shown are representative of three independent experiments with similar results. **c** XRD pattern of PW_12_, PET, and PW_12_-PET-1. PW_12_-PET-1 consisted of the amorphous PET phase with broad peaks and crystalline PW_12_ peaks (PDF#50-0304 corresponds to PW_12_·6H_2_O). **d**, **e** High resolution XPS of W 4*f* and C 1*s*, respectively. The relative proportions of characteristic peaks in the PW_12_-PET-1 composite remain largely consistent with those in pure PET and PW_12_. Due to the hydrogen bonding and electrostatic interactions formed between PW_12_ and PET, the deconvolution peaks in PW_12_-PET-1 are accompanied by a binding energy shift. **f** FTIR spectra of PW_12_, PET, and PW_12_-PET-1. The step lines indicate blue shifts of the characteristic C=O, C–O–C, and ‒CH_2_ peaks of PET in PW_12_-PET-1, while the W=O and W–O–W peaks of PW_12_ exhibit red shifts. **g** Raman spectra of PW_12_, PET and PW_12_-PET-1. Compared with PW_12_ and PET, the characteristic peaks of O‒W‒O, W‒O‒W, and W=O in PW_12_-PET-1 shift to lower wavenumbers, while the C=C and C=O peaks shift to higher wavenumbers. Source data are provided as a Source Data file.

To investigate the structural and chemical properties, the representative PW_12_-PET-1 sample was selected. As shown in Fig. 2a, the Cryo-Transmission Electron Microscopy (Cryo-TEM) image reveals that PW_12_ nanoparticles are well dispersed on the PET matrix. After magnifying the selected region highlighted by the red circle in Fig. 2a, the average particle size was statistically determined to be approximately 2.5 nm (Fig. 2b). Furthermore, X-ray diffraction (XRD) patterns confirm that these PW_12_ clusters retain their crystalline structure without decomposing. The ball-milled PET exhibits a weak and broad diffraction feature, which can be attributed to the low crystallinity of pristine PET (Fig. 2c).

The chemical states were studied via X-ray photoelectron spectroscopy (XPS, Supplementary Fig. 1). Notably, despite the absence of an external reducing agent, the W 4*f* core levels exhibit a clear shift toward lower binding energies (Fig. 2d). This shift indicates that the PW_12_ clusters have accepted electrons, leading to partial reduction. This phenomenon can be attributed to triboelectric charging of the PET polymer during ball-milling^19^. Benefiting from the intrinsic multi-electron storage capability of POMs, PW_12_ stored the electrons transferred from PET, thereby reinforcing hydrogen bonding and electrostatic interactions between them and enabling efficient PET functionalization^20,21^. Accordingly, the C 1*s* pattern of C=O and C–O–C groups in PW_12_-PET-1 displays a shift toward lower binding energies (Fig. 2e), demonstrating that PW_12_ preferentially interacts with the ester groups in PET.

The interactions between PET and PW_12_ were further investigated by Fourier-transform infrared (FTIR) spectroscopy. As shown in Fig. 2f and Supplementary Table 1, the characteristic vibrational bands of PET in PW_12_-PET-1—including those corresponding to ester groups (C=O and C–O–C), aromatic C–H, and glycol –CH_2_—exhibit distinct blue shifts, whereas the W–O–W vibrational bands of PW_12_ undergoes a red shift^9,22^. Consistently, the Raman spectra of PW_12_-PET-1 show a similar trend to that observed in the FTIR results^23,24^. As shown in Fig. 2g, the characteristic C=C and C=O bands of PW_12_-PET-1 show blue shifts, while the W=O, W–O–W and O–W–O exhibit red shifts.

Taken together, these results demonstrate that the mechanochemical functionalization proceeds through a non-destructive and non-depolymerizing pathway. During ball milling, PET transfers multiple charges to PW_12_, enabling molecular-level functionalization via hydrogen bonding and electrostatic interactions while preserving PET's structural integrity.

### NO_2_ sensing performance

Gas sensing represents a value-added application, driven by the increasing demand for real-time safety and environmental monitoring^25^. Based on the observed molecular-level integration and redox activity of PW_12_-PET-1, we selected NO_2_ sensing as a model to evaluate the gas-sensing capabilities of POM-plastic composites at room temperature. The sensor response was defined as the relative change in current upon NO_2_ exposure, calculated using the formula: *S* = (*I* − *I*_0_) / *I*_0_ × 100%, where *I* and *I*_0_ represent the current before and after gas exposure, respectively.

To identify a suitable POM for high-sensitivity POM-PET composite sensors, three commercially available Keggin-type POMs were evaluated under 100 ppm NO_2_. As shown in Supplementary Fig. 2, PW_12_ exhibited the highest response (624%), followed by tungstosilicic acid (SiW_12_, 293%) and phosphomolybdic acid (PMo_12_, 270%). Density functional theory (DFT) calculations reveal that this trend correlates with their relative Brønsted acid strengths (PW_12_ > SiW_12_ > PMo_12_), where the Brønsted acid sites serve as the exclusive adsorption sites for NO_2_, with calculated adsorption energies of −0.52 eV, −0.48 eV, and −0.45 eV, respectively (Supplementary Fig. 3). Accordingly, PW_12_ was selected for further investigation.

The effects of PW_12_ loading on elevated sensing behavior were further investigated by preparing a series of PW_12_-PET-*x* composites. As shown in Supplementary Fig. 4, PET alone shows a negligible response due to its insulating nature and poor NO_2_ adsorption capacity. In contrast, the introduction of PW_12_ markedly enhanced the sensing performance, with the exception of PW_12_-PET-1/4 in which the low PW_12_ content restricted both conductivity and active site availability. Notably, PW_12_-PET-1 achieved the highest response of 2588%, which is 4.15 times higher than that of pure PW_12_. This enhancement arises from the synergistic effects of PET-induced electronic modulation, the dispersion of partially reduced PW_12_, and improved dipole interactions with NO_2_. Moreover, at this composition, the conductivity of PW_12_-PET-1 likely meets the electron transport requirements for efficient gas sensing, thereby maximizing its performance^26^. Upon further increasing the PW_12_ content (PW_12_-PET-2, 4), the continued rise in electrical conductivity does not significantly enhance the performance. Instead, it leads to a decline due to increased grain boundary resistance caused by the uneven physical mixing of excess PW_12_.

**Fig. 3:**
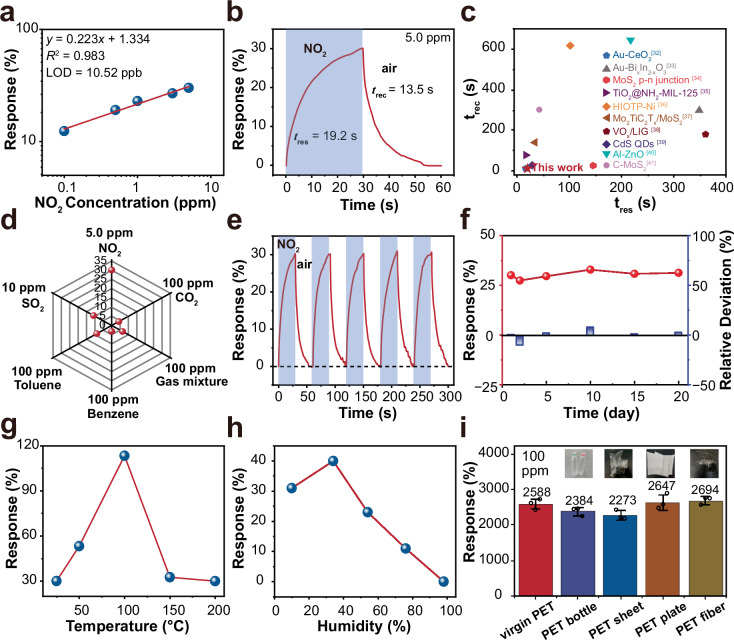
NO_2_ gas-sensing performance. **a** Log-log linear relationship between the response value and NO_2_ concentration (0.1–5.0 ppm) of PW_12_-PET-1 sample. The solid line represents a linear fit. **b** Normalized response-recovery curve of PW_12_-PET-1 sample in 5.0 ppm NO_2_. *t*_res_ and *t*_rec_ denote the time to reach 90% response and recovery, respectively. **c** Comparison of *t*_res_ and *t*_rec_ of PW_12_-PET-1 at 5.0 ppm with previous chemoreceptive materials. **d** Responses of PW_12_-PET-1 sensor to interfering various gases [inset: the gas mixture includes CH_4_ (25.63%), C_2_H_4_ (7.58%), C_2_H_6_ (7.41%), C_3_H_8_ (5.05%), C_4_H_10_ (5.12%), H_2_ (49.22%)]. CO_2_, SO_2_, and gas mixture are tested directly using standard gases; benzene and toluene are tested after being vaporized into gases. **e** Five consecutive response-recovery cycles of PW_12_-PET-1 at 5.0 ppm NO_2_. **f** Long-term cyclic stability of PW_12_-PET-1 sample towards 5.0 ppm NO_2_ (The left represents the change in the response value, while the right shows the relative deviation value of the response value). **g** Responses of PW_12_-PET-1 under different temperature conditions (25, 50, 100, 150, and 200 °C) upon exposure to 5.0 ppm NO_2_. **h** Responses of PW_12_-PET-1 under different relative humidity conditions (10%, 34%, 54%, 76%, and 98%) upon exposure to 5.0 ppm NO_2_. **i** Responses of PW_12_-PET fabricated from a variety of PET waste sources. The sensing responses of PW_12_-PET derived from reagent-grade PET (virgin) and four types of post-consumer PET waste—including discarded bottles, packaging sheets, thermoformed plates, and textile fibers—are similar under 100 ppm NO_2_. Data are shown as representative results or mean values with standard deviation error bars from at least three independent experiments. Source data are provided as a Source Data file.

Given the adverse health effects of NO_2_ even at low concentrations (0.5–5.0 ppm), particularly for sensitive populations, the low-concentration sensing performance of PW_12_-PET-1 was further evaluated across a range of 0.1–5.0 ppm^27^. As shown in Fig. 3a and Supplementary Fig. 5, PW_12_-PET-1 exhibits good log-log linearity in its response to NO_2_ (*R*^2^ = 0.983), with response values of 30% at 5.0 ppm, 19% at 0.5 ppm, and 12% even at 0.1 ppm. Based on this trend, the LOD was estimated at 10.52 ppb, demonstrating that PW_12_-PET-1 has good sensitivity for NO_2_ detection. In addition, PW_12_-PET-1 displays rapid response and recovery dynamics upon exposure to 5.0 ppm NO_2_ and subsequent air purging, with calculated response time (*t*_res_) and recovery time (*t*_rec_) values of 19.2 s and 13.5 s, respectively (Fig. 3b). Compared to reported NO_2_ chemoreceptive materials and other detection platform sensors, PW_12_-PET-1 exhibits superior real-time performance in terms of *t*_res_, *t*_rec_, and LOD (Fig. 3c, Table 1, Supplementary Fig. 6 and Supplementary Table 2)^28–41^.

**Table 1:** Compared with other detection platforms

Sensor types	Materials	Auxiliary method	Concentrations (ppm)	*t*_res_ (s)	*t*_rec_ (s)	LOD (ppb)	ref.
Optical	g-C_3_N_4_	-	5.0	30	120	3.2×10^5^	[29]
	Cu-MOF-808	-	50	160	180	16	[30]
FET	CS-FETs	-	100	83	20	1000	[31]
	Pd/WS_2_-FET	420 nm light	1.0	250	800	3.2	[32]
Chemoreceptive	PW_12_-PET-1	**-**	100	23.2	1.8	10.52	This work
		**-**	5.0	19.2	13.5		

Comparison of the *t*_res_, *t*_rec_ and LOD value of the PW_12_-PET-1 chemoreceptive sensor with previous reported optical and field-effect transistor (FET)-based sensors for NO_2_ detection

To assess the practical applicability of PW_12_-PET-1, its selectivity, stability, and recyclability were subsequently evaluated under ambient conditions. As illustrated in Fig. 3d, a series of representative atmospheric pollutants (CO_2_, SO_2_), volatile organic compounds (benzene, toluene), and a gas mixture containing CH_4_ (25.63%), C_2_H_4_ (7.58%), C_2_H_6_ (7.41%), C_3_H_8_ (5.05%), C_4_H_10_ (5.12%) and H_2_ (49.22%) were selected as potential interferents. Notably, PW_12_-PET-1 exhibits a markedly higher response to 5.0 ppm NO_2_ compared with 10 ppm SO_2_ and 100 ppm of the other gases, demonstrating excellent selectivity in complex gas environments. To evaluate its recyclability, five consecutive response-recovery cycles were conducted under 5.0 ppm NO_2_, yielding an average response of 30% and demonstrating complete recovery to the baseline (Fig. 3e). Long-term cyclic stability tests over 21 days (Fig. 3f) further confirmed excellent durability, which can be attributed to the strong hydrogen bonding and electrostatic interactions between PW_12_ and PET, as well as the rapid sensing dynamics of the composite. Additionally, the XRD and FTIR spectra after cycling reveal no significant structural changes, further verifying the long-term structural stability of PW_12_-PET-1 (Supplementary Fig. 7).

To further examine environmental adaptability, the response of PW_12_-PET-1 to varying temperatures and relative humidity (RH) was investigated. As shown in Fig. 3g and Supplementary Fig. 8a, the temperature-dependent response exhibits a volcano-shaped profile, peaking at 100 °C. The initial increase is attributed to the accelerated thermal motion and improved charge transfer, whereas the subsequent decline results from the loss of crystalline water in PW_12_ and thermal deterioration of the PET substrate^42–44^. Similarly, the effect of relative humidity was also assessed (Fig. 3h and Supplementary Fig. 8b), with a peak response at 35% RH, as moderate humidity facilitates charge transport^45^. Beyond this range, increased humidity leads to time-dependent baseline drift and slower recovery, which is detrimental to the long-term stability and reusability of the sensor (Supplementary Fig. 9)^33^. When the relative humidity approaches 100%, short-circuiting may occur due to the formation of a surface water film.

Based on these findings, we further evaluated the universality of the mechanochemical PW_12_-PET approach by preparing composites from a variety of real-life PET waste sources, including bottles, sheets, plates, and fibers. Under 100 ppm NO_2_, all composites exhibited consistently high responses (Fig. 3i), demonstrating that this strategy is broadly applicable across different PET substrates.

Overall, the high sensitivity, rapid response and recovery, superior selectivity, long-term stability, environmental adaptability, and easy availability of PW_12_-PET-1 highlight its strong potential for real-time NO_2_ monitoring in environmental and health-related applications.

### Gas-sensing mechanisms and theoretical calculations

**Fig. 4:**
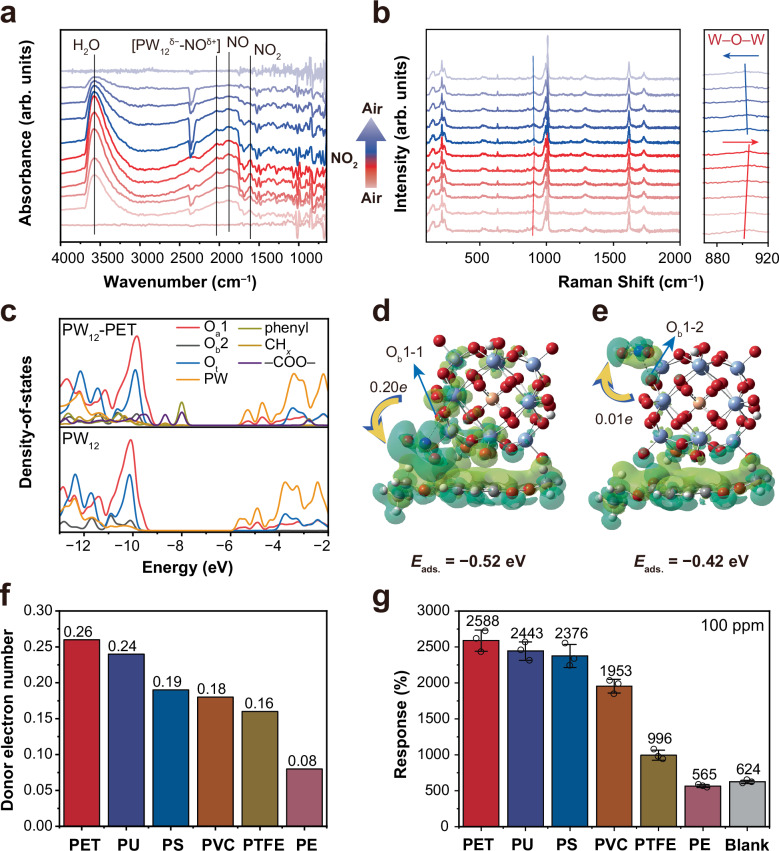
Mechanism of NO_2_ adsorption on PW_12_-PET and expanded experiments on different PW_12_-plastics. **a** In-situ DRIFTS difference spectra of PW_12_-PET-1 at room temperature. When continuously exposed to 100 ppm of NO_2_, as the exposure time increases, the peaks of NO_2_, NO, NO^+^, and −OH increase. After reaching a stable state, it was continuously exposed to the air at room temperature and then returned to its original state. **b** In-situ Raman study of the PW_12_-PET-1 at room temperature. During adsorption, the characteristic W‒O‒W peak shifts to a higher wavenumber. After being exposed to the air, the characteristic peak of W‒O‒W shift to lower wavenumbers. **c** PDOS of PW_12_ and PW_12_-PET. **d**, **e** Differential charge density distribution diagrams for NO_2_ adsorption on the O_b_1-1 (**d**, near the PET interface) and O_b_1-2 (**e**, away from PET) bridge oxygen sites of 3*e* reduced PW_12_. Here, O_b_1 and O_b_2 denote the corner-sharing and face-sharing bridging oxygen atoms, respectively, while O_t_ denotes the terminal oxygen atom. The gray-white, orange, blue, and red spheres represent H, P, W, and O atoms, respectively. **f** Theoretical predictions, and **g** experimental validations of NO_2_ sensing performance for various PW_12_-plastics (including PU, PS, PVC, PTFE, and PE; the blank is the contrast sample without plastic). The other plastics and PET were treated in the same way as PET. The theoretical and experimental results exhibit consistent trends in NO_2_ sensing performance. Data are shown as mean values with standard deviation error bars from at least three independent experiments. Source data are provided as a Source Data file and detailed DFT calculation results are provided in Supplementary Data 1.

To understand the dynamic surface reactions and charge transfer processes of PW_12_-PET-1 during NO_2_ sensing, in-situ diffuse reflectance infrared Fourier transform spectra (DRIFTS) and in-situ Raman measurements were performed. As shown in Fig. 4a, upon exposure to NO_2_, DRIFTS reveals that NO_2_ is rapidly adsorbed on the PW_12_-PET-1 surface, as evidenced by the characteristic NO_2_ band observed at 1621 cm^−1^. Following adsorption, NO_2_ accepts electrons from the sensor and reacts with the protons around adsorption sites to form NO and H_2_O, as indicated by the appearance of peaks at 1894 cm^−1^ and 3582 cm^−1^, respectively^46–48^. Moreover, a strong signal at 2033 cm^−1^, attributed to the NO^δ+^ intermediate appears^49^, reflecting a strong interaction between NO and PW_12_. Concurrently, in-situ Raman spectra (Fig. 4b) show that the W–O–W bridge-oxygen peak gradually shifts to higher wavenumbers during NO_2_ adsorption, providing direct evidence for electron transfer from PW_12_-PET-1 to NO_2_. Thus, the corresponding adsorption pathways can be summarized as follows:

1 $${{\mathrm{NO}}}_{2}+2{e}^{-}+2{\mathrm{H}}^{+}\to {\mathrm{NO}}+{\mathrm{H}}_{2}{\mathrm{O}}$$

2 $${{\mathrm{NO}}}+{{\mathrm{PW}}}_{12}\to [{{\mathrm{PW}}}_{12}^{\delta -}-{{\mathrm{NO}}}^{\delta+}]$$

Subsequently, the reversibility of PW_12_-PET-1 was confirmed during desorption. Upon switching to air, the NO_2_ adsorption peak and the intermediate signals in DRIFTS difference spectra rapidly declined, while the W–O–W peak in Raman returned to its initial position. This indicates that the adsorption-desorption reaction is highly reversible, with NO and H_2_O undergoing an inverse reaction to regenerate NO_2_ and H^+^, thereby restoring the initial electronic state.

3 $$[{{\mathrm{PW}}}_{12}^{\delta -}-{{\mathrm{NO}}}^{\delta+}]\to {\mathrm{NO}}+{{\mathrm{PW}}}_{12}$$

4 $${\mathrm{NO}}+{\mathrm{{H}}_{2}O}\to {\mathrm{N{O}}_{2}}+{2e}^{-}+{\mathrm{2H}}^{+}$$

To deep elucidate the excellent NO_2_ adsorption response mechanism of PW_12_-PET composite, density functional theory (DFT) calculations were further performed. A simplified PET model comprising a single repeat unit capped with alkyl groups was employed, as its frontier orbital characteristics and band gap remain nearly unchanged with increasing chain length (Supplementary Fig. 10a–c). Subsequently, the optimized structure of PW_12_-PET composite shows that the C=O group of the PET ester forms a hydrogen bond with the Brønsted acidic proton of PW_12_ (Supplementary Fig. 10d). Differential charge density analysis further reveals pronounced electrostatic and weak van der Waals interactions between PET and PW_12_, leading to an electron transfer of approximately 0.26*e* from PET to PW_12_ (Supplementary Fig. 11). This interfacial charge redistribution significantly modulates the electronic band structure of the composite. Projected density of states (PDOS) analysis shows that the HOMO of PW_12_-PET is primarily derived from the phenyl and ester orbitals of PET, while the LUMO is mainly localized on the bridge O_b_1 and the P and W atoms of PW_12_ (Fig. 4c). Owing to its strong electron-accepting character, PW_12_ can capture the multiple charges from PET during ball-milling, leading to partial reduction^50^. Moreover, the spatial separation of the HOMO and LUMO across PET and PW_12_ effectively suppresses charge recombination and enhances the reduction degree of PW_12_ within the composite.

Considering that the reduction state of PW_12_ within PW_12_-PET composite substantially influences NO_2_ adsorption behavior, adsorption energies and associated charge transfer values were calculated for the 0*e*, 1*e*, 2*e*, and 3*e* reduced states. As illustrated in Supplementary Fig. 12a–c, PW_12_ exhibits a strong electron-accepting character from 0*e* to 2*e*, with NO_2_ preferentially adsorbing at the Brønsted acid sites. However, increased reduction enhances electrostatic repulsion, weakening the interaction between PW_12_-PET and NO_2_. While at the 3*e* reduced state, the NO_2_ adsorption behavior changes markedly. As shown in Supplementary Fig. 12d, the direction of charge transfer reverses, with PW_12_-PET donating approximately 0.30*e* to NO_2_, and the adsorption energy at the Brønsted acid site markedly increases to −1.57 eV. Due to the strong electron delocalization inherent in PW_12_ clusters, the bridging oxygen atoms of O_b_1 are activated as additional active sites, with O_b_1-1 and O_b_1-2 adsorption energies of −0.52 eV and −0.42 eV, respectively (Fig. 4d, e). Given that PET preferentially wraps around Brønsted acid sites during ball-milling, the accessible O_b_1 atoms serve as the dominant adsorption centers. Thus, these findings establish that the improved response of PW_12_-PET-1 results from the formation of a certain reduced degree of PW_12_, which increases the number of accessible active sites and possesses moderate adsorption-desorption energetics.

### Extensions and practical application

To verify the broader applicability of waste plastics for PW_12_-plastic sensors, five common polymers—polyurethane (PU), polystyrene (PS), polyvinyl chloride (PVC), polytetrafluoroethylene (PTFE), and polyethylene (PE)—were evaluated alongside PET. DFT calculations were primarily performed to assess the electron-donating abilities of these plastics to PW_12_. As shown in Fig. 4f and Supplementary Fig. 13, the calculated electron transfers from PU, PS, PVC, PTFE, and PE to PW_12_ are 0.24*e*, 0.19*e*, 0.18*e*, 0.16*e*, and 0.08*e*, respectively. Although these values are slightly lower than that of PET (0.26*e*), PU, PS, PVC, and PTFE still demonstrate potential as plastic precursors.

To experimentally validate this, PW_12_-plastic composites were fabricated using the same optimized PW_12_ content as used in PW_12_-PET-1 (Supplementary Figs. 14–18). Then, their sensing performance was evaluated at room temperature in the presence of 100 ppm NO_2_. As shown in Fig. 4g, the observed NO_2_ sensing responses closely follow the DFT-predicted electron-donating trend. This correlation can be attributed to the different intrinsic electronic properties of the polymer functional groups and their resulting interfacial interactions with PW_12_. Specifically, in PW_12_-PET-1, PW_12_-PU, and PW_12_-PS, electron-rich groups of phenyl ester (–C_6_H_5_COO–), urethane (–NHCOO–), and phenyl (–C_6_H_5_) facilitate favorable interfacial electronic coupling with PW_12_ via π–π or n–π* orbital interactions. These interactions likely enhance charge transfer, resulting in significantly improved sensing responses of 2588%, 2443%, and 2376%, respectively. In contrast, PW_12_-PVC and PW_12_-PTFE exhibit weaker interfacial interactions due to the high electronegativity of Cl and F atoms, resulting in lower responses of 1953% and 996%, respectively. For PE, the absence of polar or electron-donating functional groups limits interfacial charge exchange. As a result, PW_12_ remains primarily physically mixed, leading to high interfacial resistance and poor charge transport. Consequently, its response drops to just 565%.

The NO_2_ sensing performance of PW_12_-PU, PW_12_-PS, and PW_12_-PVC was further evaluated within a low concentration range of 0.1 to 10 ppm. As illustrated in Supplementary Fig. 19, they all exhibit good log-log linearity (*R*^2^ = 0.968–0.997) and low LODs of 12.31, 16.81, and 11.71 ppb, respectively, indicating high sensitivity. Meanwhile, they exhibit fast response and recovery behaviors: the *t*_res_ values are 23.0 s, 14.6 s and 12.4 s and the *t*_rec_ values are 19.8 s, 20.8 s and 7.1 s for PW_12_-PU, PW_12_-PS, and PW_12_-PVC, respectively (Supplementary Fig. 20). Although their selectivity toward NO_2_ is lower than that of PW_12_-PET-1 due to differing functional groups (Supplementary Fig. 21), these results highlight the potential of tuning sensing behavior through plastic selection. This approach offers a versatile strategy for multi-scenario sensing while enabling waste plastic recycling. Finally, PW_12_-PET-1 was selected for device integration to showcase its practical applicability (Supplementary Figs. 22–24). As shown in the Supplementary Movie 1, the system triggers a real-time LED and buzzer alarm once the NO_2_ concentration surpasses 5.0 ppm, illustrating its promise for environmental and health monitoring.

In summary, we present a facile, waste-free, and environmentally benign mechanochemical ball-milling strategy for directly non-depolymerizing upcycling waste plastics into POM-plastic composites for NO_2_ sensing. Among these composites, PW_12_-PET-1 exhibits the best sensing performance at room temperature, with fast *t*_res_/*t*_rec_ of 19.2 s/13.5 s at 5.0 ppm NO_2_ and a LOD value of 10.52 ppb, outperforming most reported NO_2_ sensors. Moreover, it exhibits high selectivity against ten interfering gases and retains long-term stability. Mechanistic studies revealed that PET acts as an electron donor, transferring multiple charges to PW_12_ in PW_12_-PET-1. DFT calculations indicate that this charge transfer can activate the additional bridging oxygen sites on PW_12_, thereby enhancing NO_2_ adsorption while maintaining moderate adsorption strength. In-situ DRIFTS difference spectra and Raman measurements confirm that the NO_2_ adsorption-desorption process is highly reversible, with NO and H_2_O regenerating NO_2_ and restoring the initial electronic state. Expanded investigations demonstrate that waste plastics with electron-rich functional groups can also be applied for PW_12_-plastic sensors. Although their selectivity toward NO_2_ is poor, they may hold potential for the development of other specialized gas sensors in future studies. Notably, a practical PW_12_-PET-1 device triggers a real-time LED and buzzer alarm when NO_2_ concentration exceeds 5.0 ppm, confirming its applicability. Overall, this work offers a green strategy for the direct non-depolymerizing upcycling of waste plastics into functional materials, and may spark broader interest in sustainable plastic waste management.

## Methods

### Synthesis of PW_12_-PET-*x* composites

Polyethylene terephthalate (PET, granular, Macklin) was first ground into fine powder using a batch mill (A10 basic, IKA), and then passed through a 100-mesh sieve. Subsequently, PET (0.500 g, 2.60 mmol), phosphotungstic acid (PW_12_, AR, Macklin; 0.624 g, 0.22 mmol; 1.248 g, 0.43 mmol; 2.496 g, 0.87 mmol; 4.992 g, 1.73 mmol; and 9.984 g, 3.47 mmol; respectively) and H_2_O (5 ml) were added to a custom-made 90 ml agate ball-mill jar. Then, the mixture was ball-milled at 300 rpm for 12 h using Retsch PM 100 CM machine with a transmission ratio (1: −1). To prevent excessive heat buildup from mechanical friction, milling was paused for 10 mins after every 60 mins of operation. After ball-milling, the resulting product was dried at 70 °C for 12 h to obtain the PW_12_-PET-*x* composites, where *x* denotes the molar ratio of H^+^ in PW_12_ to PET.

### Performance comparison of different Keggin-type POMs

To identify suitable POMs for fabricating high-sensitivity POM-PET composite-based NO_2_ sensor devices, two additional commercially available Keggin-type POMs, tungstosilicic acid (SiW_12_, AR, Macklin) and phosphomolybdic acid (PMo_12_, AR, Macklin), were selected for comparative experiments alongside PW_12_. To maintain a constant molar ratio of H^+^ to PET, the amounts of PET and water were kept unchanged, while equivalent H^+^ molar amounts of SiW_12_ (1.871 g, 0.87 mmol) and PMo_12_ (1.582 g, 0.87 mmol) were added. Subsequently, the same ball-milling method used for PW_12_-PET-1 was applied, and the SiW_12_-PET and PMo_12_-PET composites were obtained after drying.

### Extension to other plastics

Polyurethane (PU, Merck) and polystyrene (PS, Aladdin) are both supplied in granular form. Polyvinyl chloride (PVC, Macklin), polytetrafluoroethylene (PTFE, Macklin), and polyethylene (PE, Macklin) are provided as powders. Their pretreatment followed the same procedure as that of PET: grinding into fine powder using a batch mill, followed by sieving through a 100-mesh screen.

To maintain the same molar amounts of PET as in PW_12_-PET-1, the as-prepared powders of PVC (0.163 g, 2.60 mmol), PU (0.787 g, 2.60 mmol), PS (0.271 g, 2.60 mmol), PTFE (0.260 g, 2.60 mmol) and PE (0.073 g, 2.60 mmol) are respectively added to the ball-milling jar together with PW_12_ (2.496 g) and water (5 ml). Subsequently, the same ball-milling method used for the preparation of PW_12_-PET-*x* was adopted, and the resulting products of PW_12_-PVC, PW_12_-PU, PW_12_-PS, PW_12_-PTFE, and PW_12_-PE were obtained after drying, respectively.

### Extension to **real post-consumer PET waste**

The PET waste (discarded bottles, packaging sheets, thermoformed plates, and textile fibers) followed the same procedure as virgin PET: grinding into a fine powder using a batch mill, then sieving through a 100-mesh screen. Subsequently, the same ball-milling method used for the preparation of PW_12_-PET was adopted, and four types of PW_12_-PET were obtained after drying.

### Fabrication of gas sensor

5 mg of POM-PET powder were dispersed in 100 μl of the acetone-ethanol mixed solvent (CH_3_COCH_3_:EtOH = 6:4), and ultrasonically treated for 10 mins to ensure uniform dispersion. Then, a 4 μl aliquot of the dispersion was drop-cast onto interdigitated electrodes with 10 pairs of fingers and 50 μm gaps. After drying, the sensor was ready for gas-sensing measurements.

### Gas sensing measurements

The testing procedure employed the conventional static method. All measurements were conducted under laboratory conditions using a multichannel potentiostat (CHI660E, Shanghai Chenhua Instrument Co., Ltd.) in “*i*-*t*” mode. Initial sensing tests were carried out under exposure to 100 ppm NO_2_ at 25 °C. Based on the results, PW_12_-PET-1, which exhibited the best performance, was selected for a series of subsequent tests. The temperature-dependent and humidity-dependent tests employed the single-factor variable method. While keeping other experimental conditions constant, the temperature was varied (25, 50, 100, 150, and 200 °C) during exposure to 5.0 ppm NO_2_. Correspondingly, the relative humidity (RH) was independently adjusted (10%, 34%, 54%, 76%, and 98%) under a constant 5.0 ppm NO_2_.

### Characterizations

X-ray diffraction (XRD) measurements were performed using a Rigaku MiniFlex 600-C diffractometer with a monochromated Cu *K*α radiation source. Cryo-Transmission Electron Microscopy (Cryo-TEM) images were carried out using a field-emission transmission electron microscope (Talos F200C, FEI) with 200 keV accelerating voltage. The Fourier transform infrared (FTIR) spectra was performed on Cary 630 (Agilent). The diffuse reflectance infrared Fourier transform spectra (DRIFTS) were performed on Nicolet iS10 (Thermo Fisher), spanning 600–4000 cm^–1^, and controlled by OMINIC software. Raman and in-situ Raman spectra were recorded using a Renishaw micro-Raman spectrometer with a 532 nm excitation laser. X-ray photoelectron spectra (XPS) were acquired using an AXIS Supra^+^ spectrometer, with Al-*K*α radiation (1486.6 eV) as the X-ray light source and referencing the C *sp*^2^ peak at 284.70 eV for PET.

### Mathematical formulation

The sensor response was defined as the relative change in current before and after exposure to the target gas, calculated using the formula:

5 $${Response}(\%)=\frac{I-{I}_{0}}{{I}_{0}}\times 100\%$$

where *I* is the current value before contact with the gas and *I*_0_ is the current value after contact with the gas. The limit of detection (LOD) is defined as a concentration of the analyte that causes a response 3 times higher than the noise level of the device. Using Eqs. (6) and (7), the root-mean-square *RMS*_noise_ was calculated to be 0.000782.

6 $${V}_{{x}^{2}}={\sum }_{i=1}^{N}({Re}_{i}-{\overline{Re}})$$

7 $${RM}{S}_{{\mathrm{noise}}}=\sqrt{\frac{{V}_{{x}^{2}}}{N}}$$

$\it {{Re}}_{i}$ represents the measured data point, $\it {\overline{Re}}$ is the mean of the baseline data, and *N* is the number of data points (50 data points were used in the curve fitting).

8 $${LOD}=3\times \frac{{{RMS}}_{{\mathrm{noise}}}}{{Slope}}$$

where *RMS*_noise_ represents the noise value calculated through root-mean-square deviation from the baseline and the *Slope* was obtained from the inset of log-log linear relationship^51^.

### Computational details

DFT calculations of the NO_2_ adsorption mechanism on PW_12_-PET were implemented using the Gaussian 16 package at the B3-LYP level without symmetry restrictions^52,53^. To account for weak interactions between PW_12_ and plastic matrix, the B3LYP-D3(BJ) dispersion-corrected functional was employed^54^. Throughout all calculations, the LANL2DZ basis set was employed for W, whereas the 6–31 G (d, p) basis set was used for P, O, H, and C atoms^55–57^. Partial density-of-states (PDOS) analysis, with a full width at half maximum (FWHM) of 0.01 calculation was carried out using the Multiwfn 3.8 program^58^.

### Reporting summary

Further information on research design is available in the Nature Portfolio Reporting Summary linked to this article.

## Supplementary information

Supplementary Information

Description of Additional Supplementary File

Supplementary Data 1

Supplementary Movie 1

Reporting Summary

Transparent Peer Review file

## Source data

Source Data

## Data Availability

The data that were generated in this study are presented in the main text and Supplementary Information. Source data are provided with this paper.
