# Supplementary material for: Mechanochemically functionalized waste plastics for NO2 sensing
Source: Nat Commun. 2026 Apr 25;17:5745. doi: 10.1038/s41467-026-72492-8 (PMC13324026; doi:10.1038/s41467-026-72492-8)
Supplement: Supplementary file 1 — Supplementary Information [file 41467_2026_72492_MOESM1_ESM.pdf]

Supplementary Information

for

Mechanochemically functionalized waste plastics for NO_2 sensing

Yingnan Zhao, Liang Pang, Zhao Zhao, Hang Shi, Renquan Guan, Zhongling Lang, Ruiqi Yao, Tonghui Wang, Zhimin Ao, Huaqiao Tan*, Gao-Feng Han*, Xing-You Lang and Qing Jiang*

Correspondence to: Gao-Feng Han, gfhan@jlu.edu.cn; Qing Jiang, jiangq@jlu.edu.cn; Huaqiao Tan, tanhq870@nenu.edu.cn

This Supplementary Information includes:

Supplementary Figures 1–24

Supplementary Tables 1,2

Supplementary References

Supplementary Figures

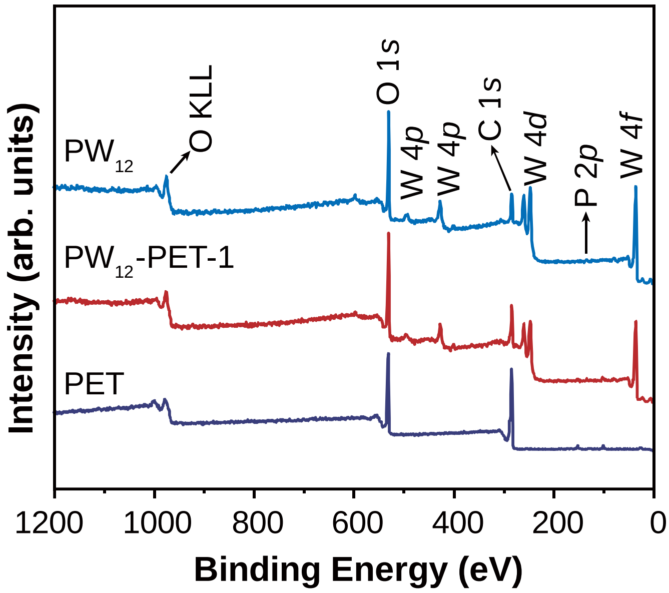

Supplementary Fig. 1 | X-ray photoelectron spectroscopy. XPS survey spectra of PW_{12}, PW_{12}-PET-1 and PET. Source data are provided as a Source Data file.

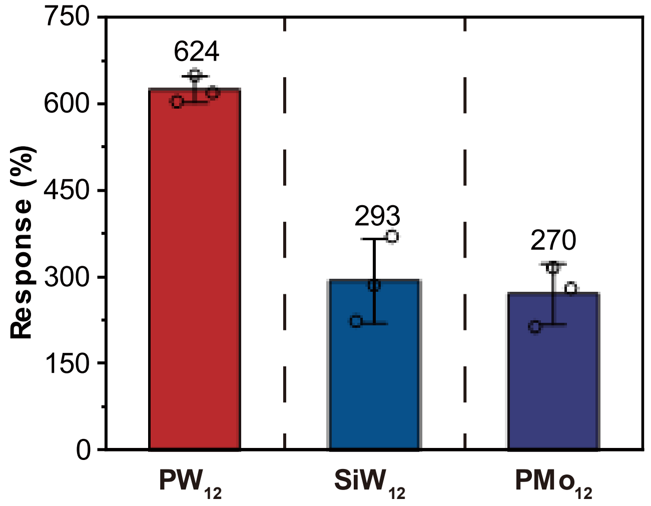

Supplementary Fig. 2 | Comparisons of three typical Keggin-type POMs. Gas sensing performance of commercially available Keggin-type POMs of PW_{12}, SiW_{12} and PMo_{12} towards 100 ppm NO_2. Error bars represent the standard deviation of at least three independent measurements. Data are shown as mean values with standard deviation error bars from at least three independent experiments. Source data are provided as a Source Data file.

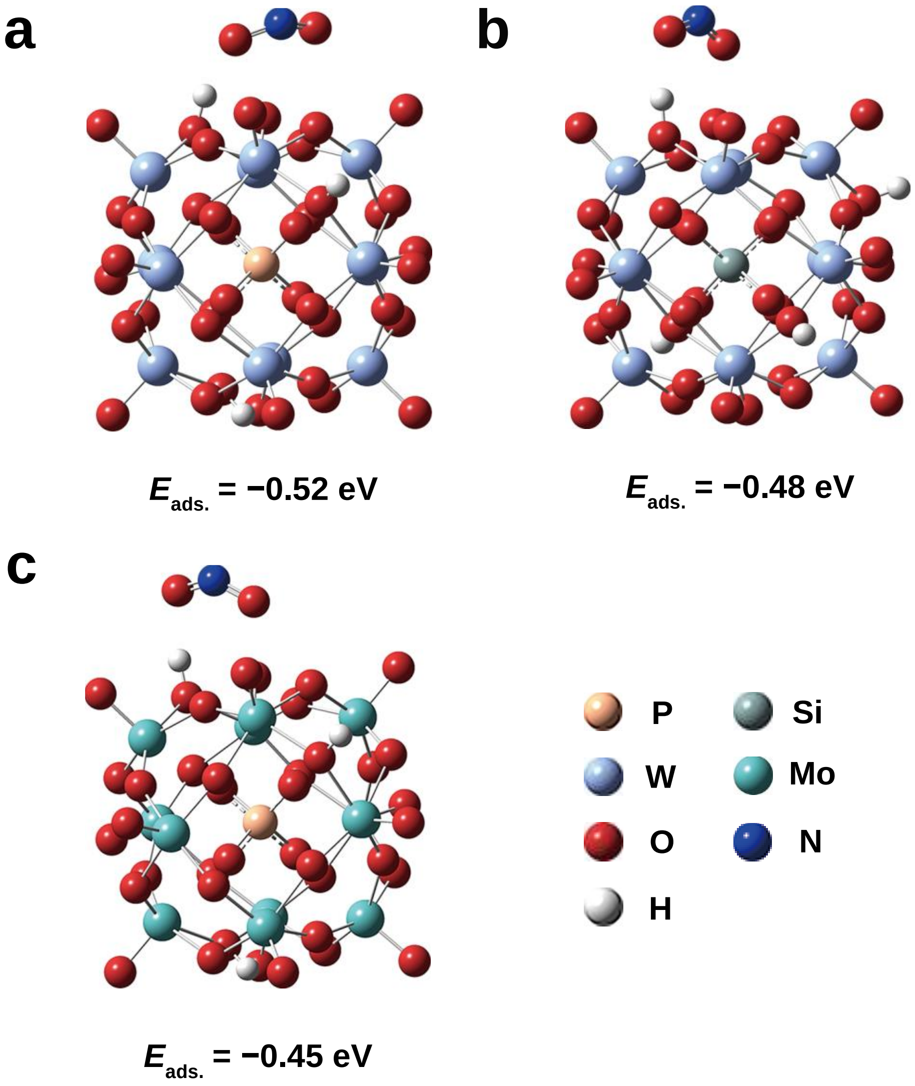

Supplementary Fig. 3 | NO_2 adsorption on three typical Keggin-type POMs. Optimized NO_2 adsorption models and corresponding adsorption energies for **a**, PW_{12}, **b**, SiW_{12} and **c**, PMo_{12}, respectively. The trend in adsorption energies of NO_2 is consistent with their experimental NO_2 response values and correlates with their relative Brønsted acid strengths (PW_{12} > SiW_{12} > PMo_{12}). Detailed DFT calculation results are provided in Supplementary Data 1.

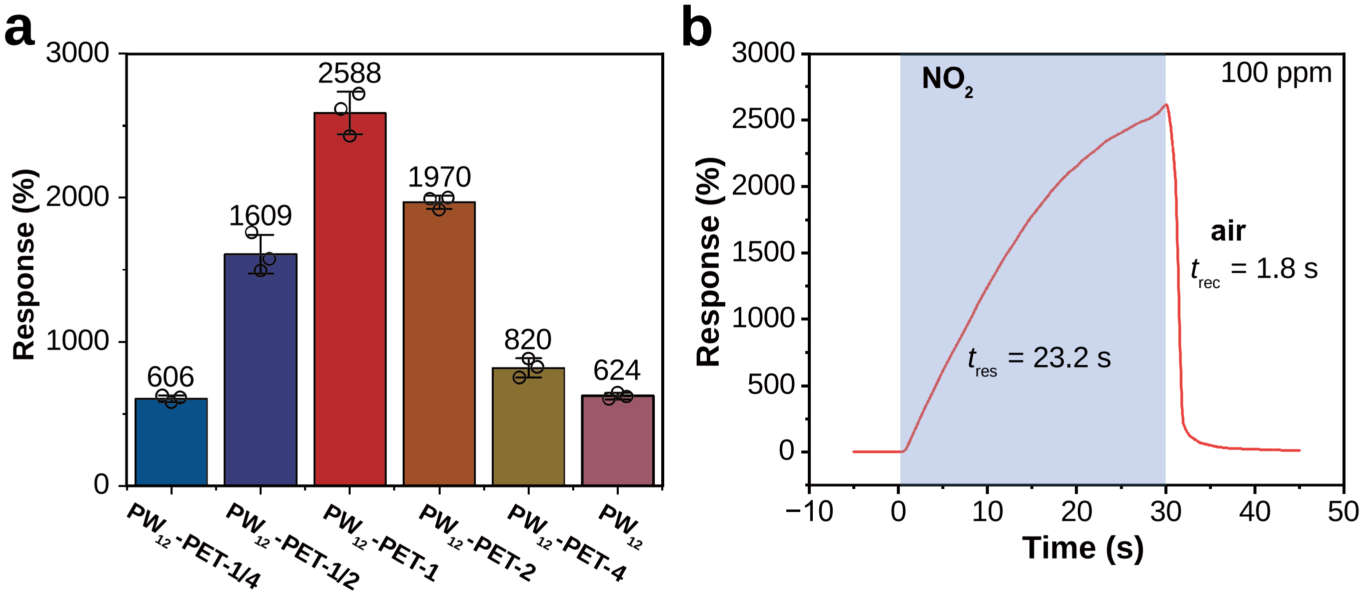

Supplementary Fig. 4 | Gas sensing performance of PW_{12}-PET-x (x = 1/4, 1/2, 1, 2, 4) samples in 100 ppm NO_2. a, Response value of PW_{12}-PET-x to 100 ppm NO_2. PW_{12}-PET-1 achieved the highest response of 2588%. **b**, Normalized response-recovery curve of PW_{12}-PET-1 sample in 100 ppm NO_2. The response time (t_{res}) and the recovery time (t_{rec}) denote the time to reach 90% response and recovery, respectively. The blue shaded area corresponds to NO_2 exposure, and the left area to air. Data are shown as representative results or mean values with standard deviation error bars from at least three independent experiments. Source data are provided as a Source Data file.

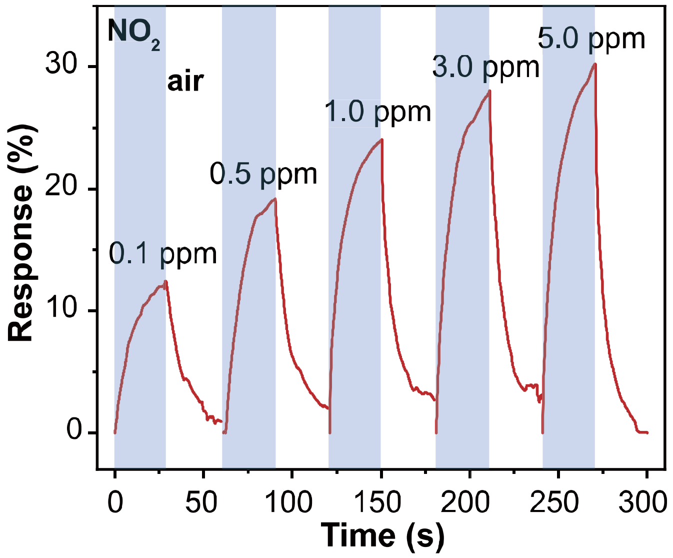

Supplementary Fig. 5 | Gas sensing performance of PW_{12}-PET-1 for NO_2 detection across a concentration range of 0.1–5.0 ppm. The response increases upon exposure to NO_2 and decreases after returning to air. Data shown are representative of three independent experiments with similar results. Data are shown as representative results from at least three independent experiments. Source data are provided as a Source Data file.

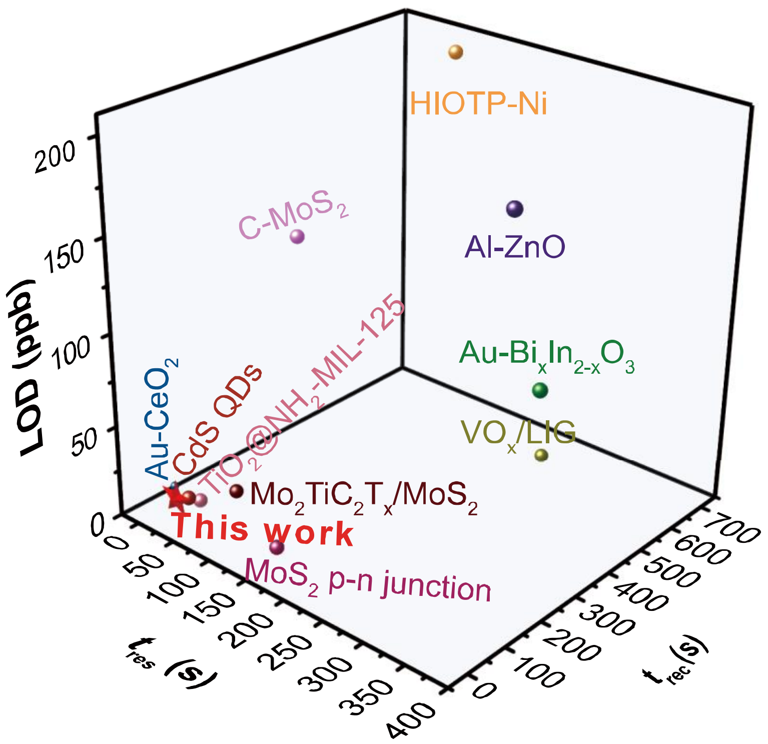

Supplementary Fig. 6 | Comparison of t_{res}, t_{rec} and LOD value of PW_{12}-PET-1 at 5.0 ppm with previous chemoreceptive materials. Compared to other reported NO_2 chemoreceptive materials, PW_{12}-PET-1 exhibits superior real-time performance in terms of t_{res}, t_{rec}, and LOD. Source data are provided as a Source Data file.

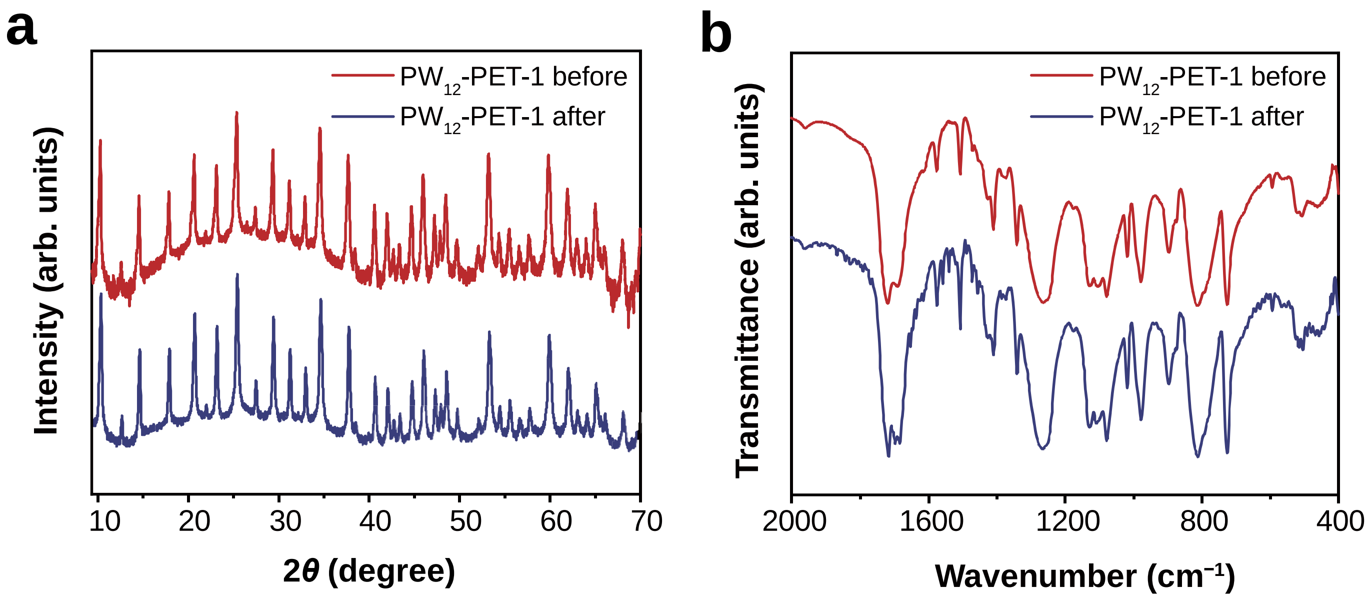

Supplementary Fig. 7 | Characterizations of PW_{12}-PET-1 before and after the tests. a, XRD and **b**, FTIR patterns of PW_{12}-PET-1 before and after long-term cyclic stability tests over 21 days. Source data are provided as a Source Data file.

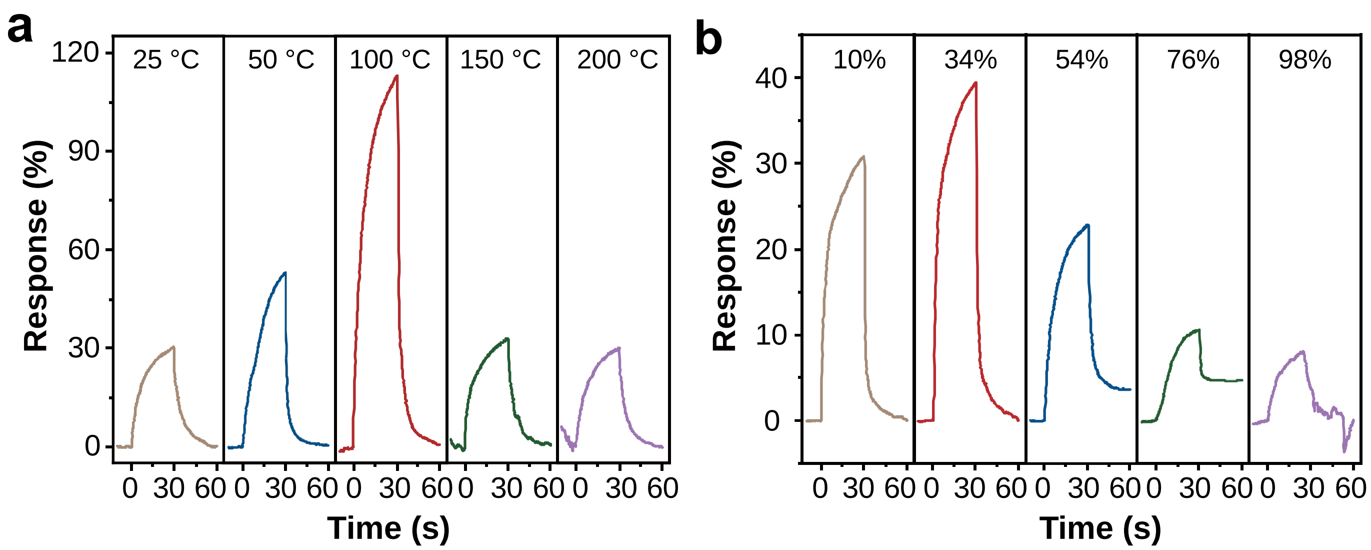

Supplementary Fig. 8 | Gas sensing performance of PW_{12}-PET-1 for NO_2 detection under different environment. a, Normalized response-recovery curve of PW_{12}-PET-1 under different temperature conditions (25, 50, 100, 150 and 200 °C) upon exposure to 5.0 ppm NO_2. **b**, Normalized response-recovery curve of PW_{12}-PET-1 under different relative humidity conditions (10%, 34%, 54%, 76%, and 98%) upon exposure to 5.0 ppm NO_2. Data are shown as representative results from at least three independent experiments. Source data are provided as a Source Data file.

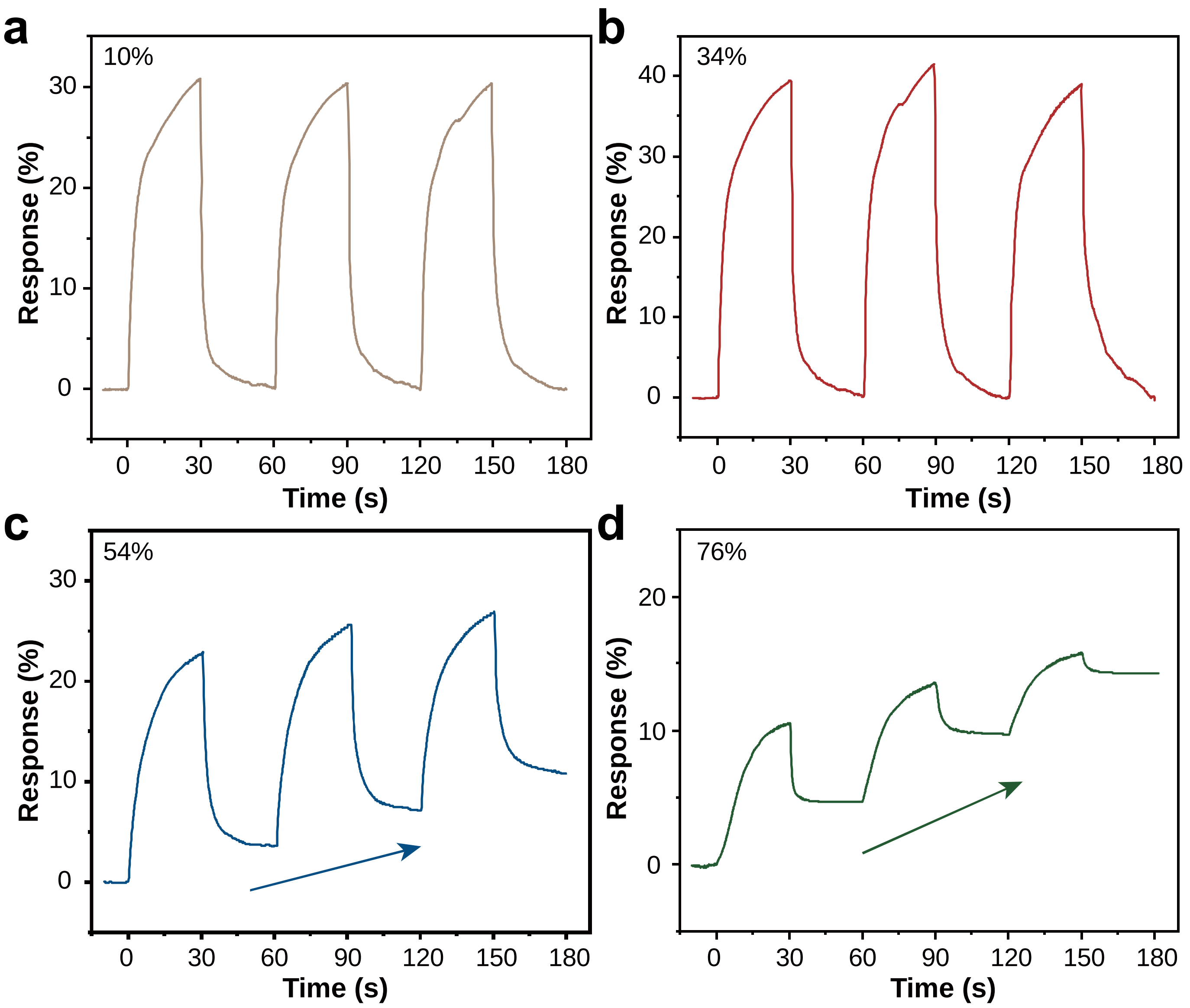

Supplementary Fig. 9 | Repetitive response-recovery cycles of PW12-PET-1 toward 5.0 ppm NO_2 at various relative humidity (RH) levels (10%, 34%, 54%, and 76%). The sensor exhibits high cyclic stability and baseline consistency at 10% RH (a) and 34% RH (b). Gradual baseline drift and decreased responses are observed at 54% RH (c) and 76% RH (d). Data are shown as representative results from at least three independent experiments. Source data are provided as a Source Data file.

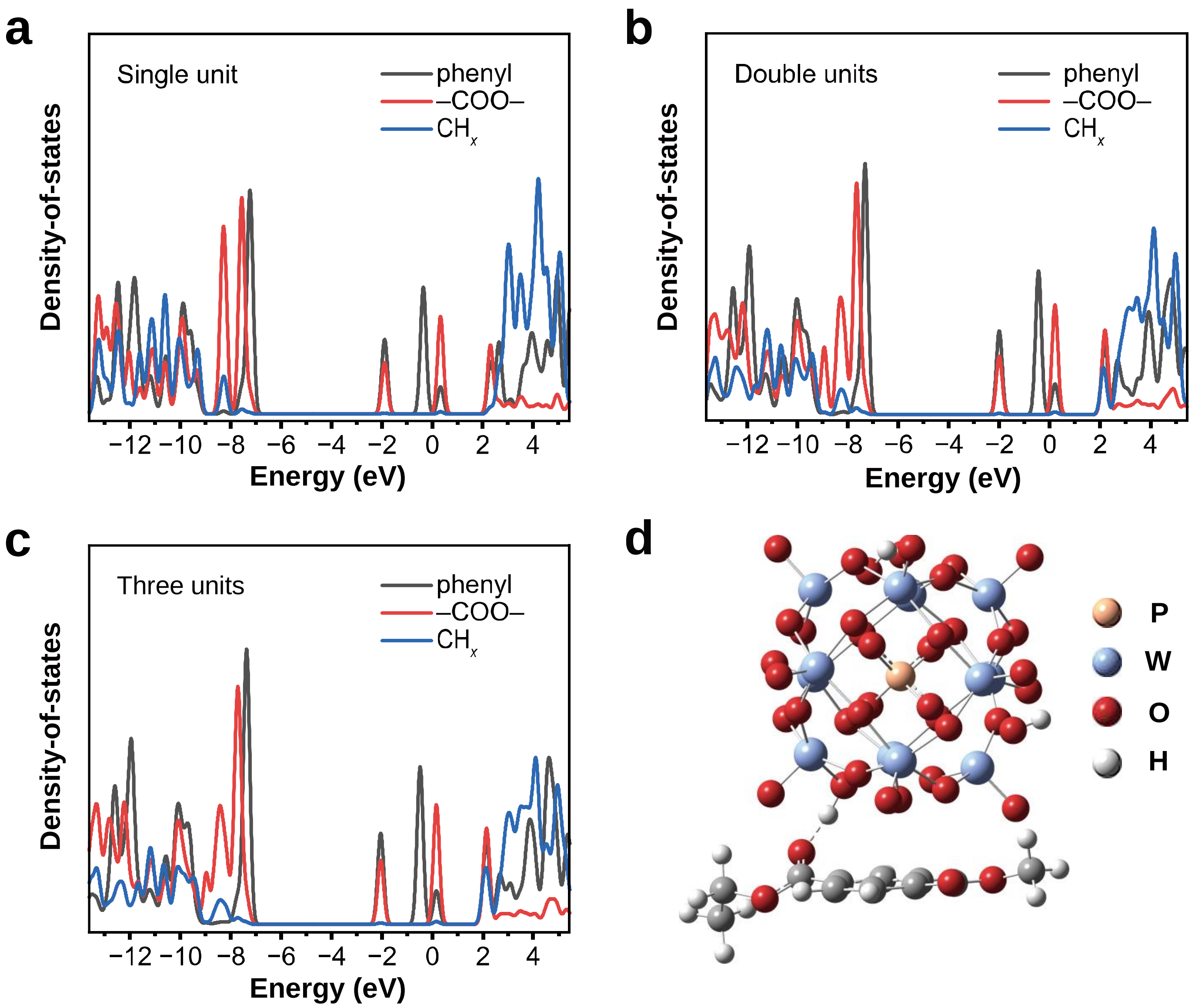

Supplementary Fig. 10 | Structural model determination of PW_{12}-PET. The projected density of states (PDOS) of **a**, single, **b**, double and **c**, three units of PET. The frontier orbital characteristics and band gap remain nearly unchanged with increasing chain length. **d**, The optimized structure of the representative PW_{12}-PET composite. Detailed DFT calculation results are provided in Supplementary Data 1.

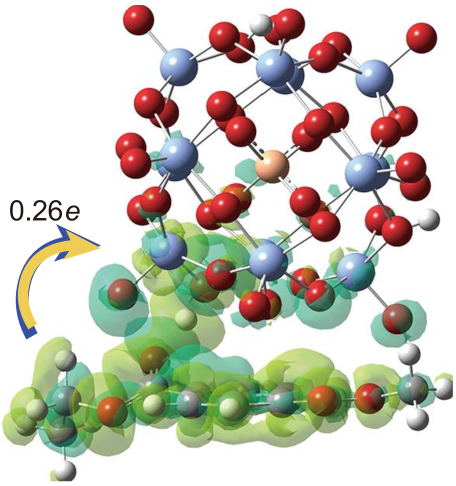

Supplementary Fig. 11 | Differential charge density diagram of PW_{12}-PET. It reveals pronounced electrostatic and weak van der Waals interactions between PET and PW_{12}, leading to an electron transfer of approximately 0.26*e* from PET to PW_{12}. Detailed DFT calculation results are provided in Supplementary Data 1.

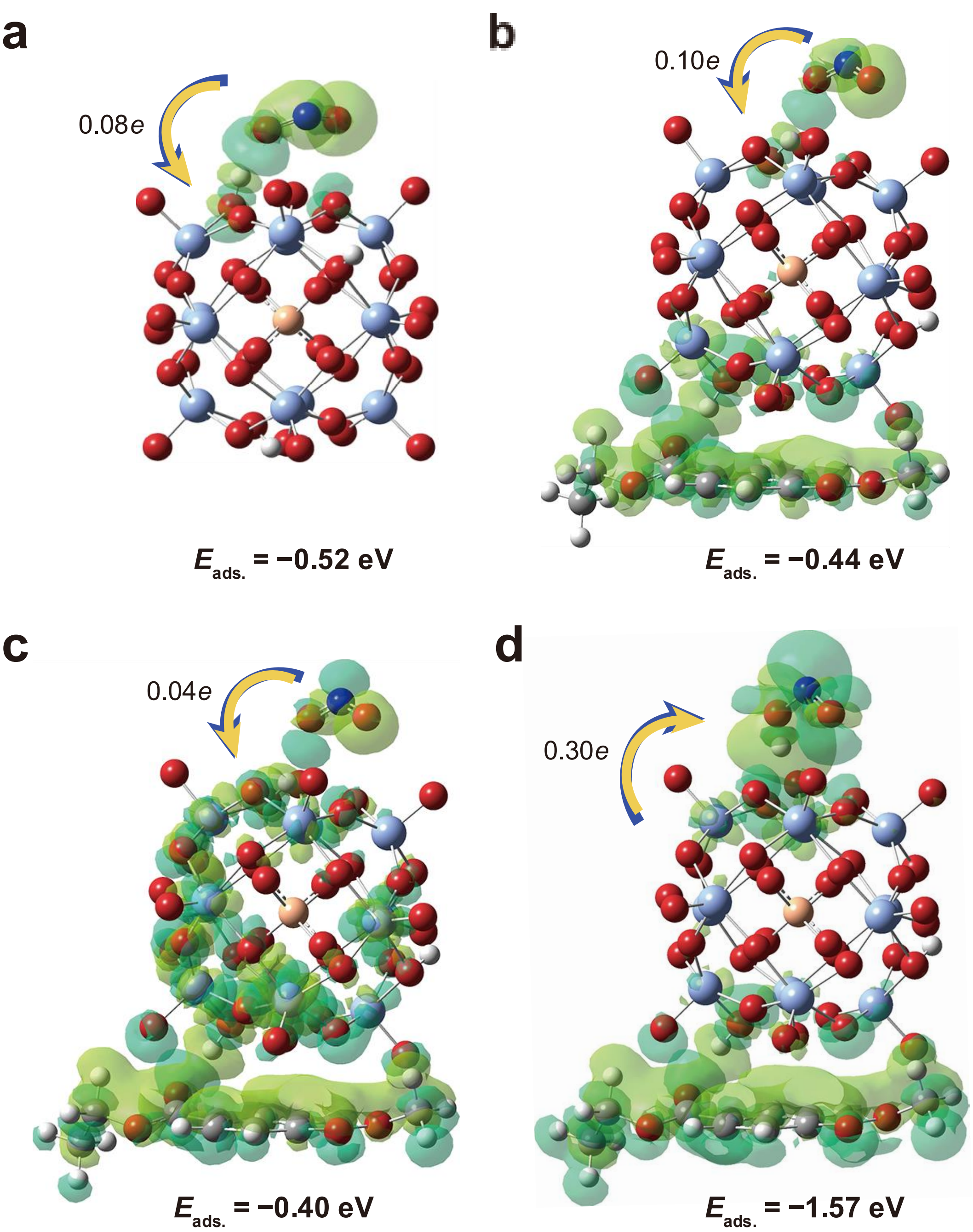

Supplementary Fig. 12 | Differential charge density distribution for NO_2 adsorption on PW_{12} and reduced PW_{12}-PET. The differential charge density distribution diagrams for NO_2 adsorption on **a**, PW_{12}, **b**, 1*e*, **c**, 2*e* and **d**, 3*e* reduced PW_{12}-PET Brønsted acid sites, respectively. Detailed DFT calculation results are provided in Supplementary Data 1.

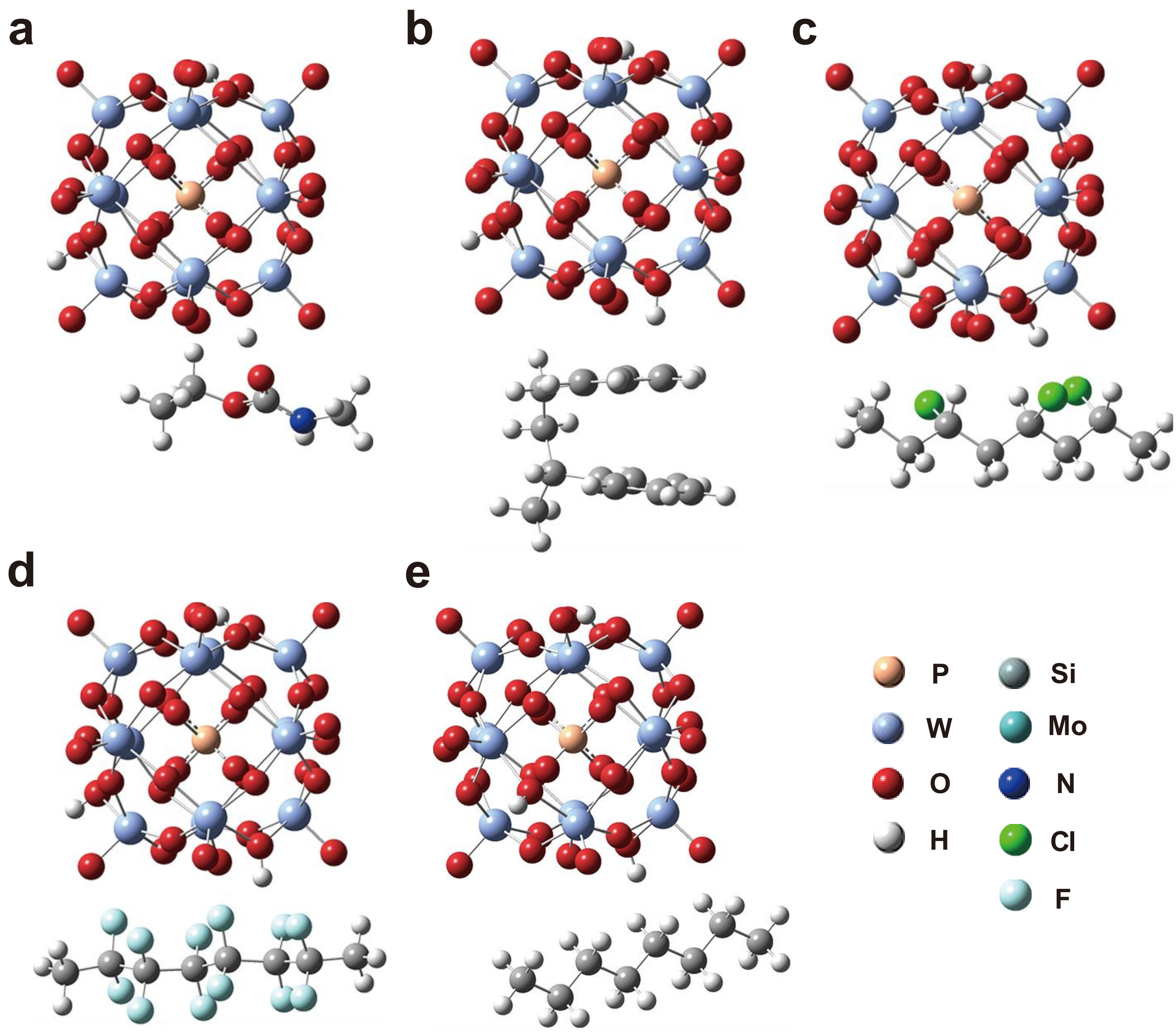

Supplementary Fig. 13 | Simplified models of PW_{12}-plastics with different functional groups. **a**, PW_{12}-PU, **b**, PW_{12}-PS, **c**, PW_{12}-PVC, **d**, PW_{12}-PTFE, **e**, PW_{12}-PE. All simplified plastic models were rigorously constructed based on a series of frontier orbital characteristic calculations, as done for PET. Detailed DFT calculation results are provided in Supplementary Data 1.

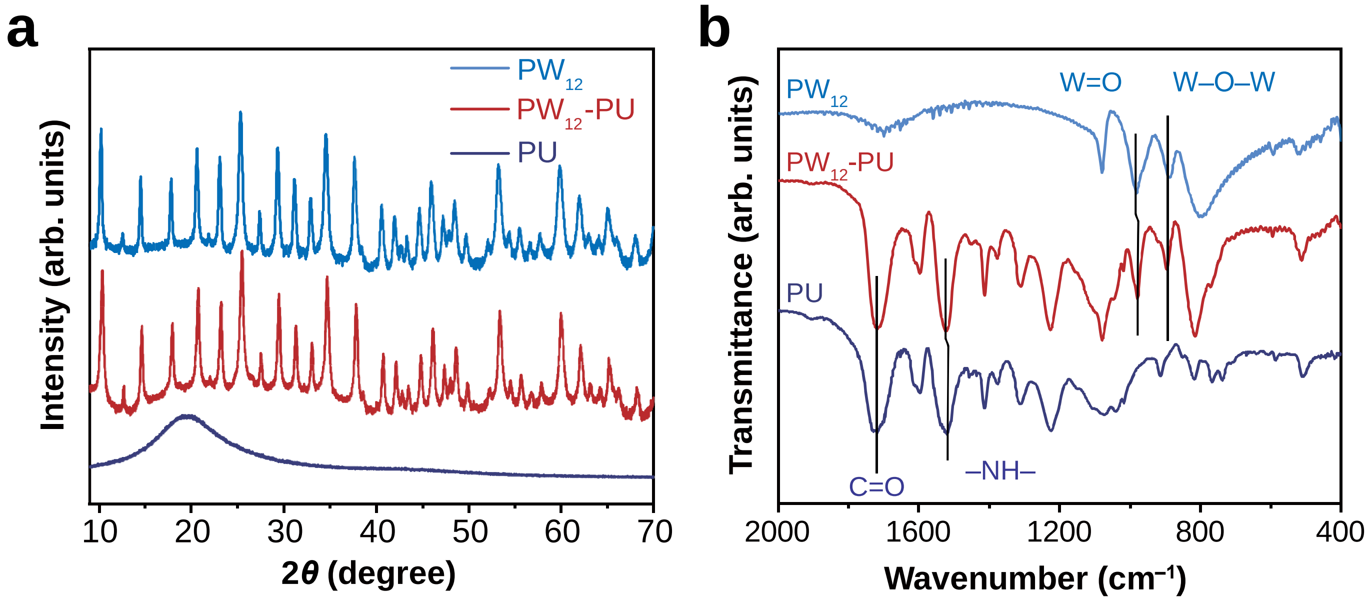

Supplementary Fig. 14 | Characterization of PW_{12}-PU extension composite. a, XRD and **b**, FTIR spectra of PW_{12}, PU, and PW_{12}-PU. Source data are provided as a Source Data file.

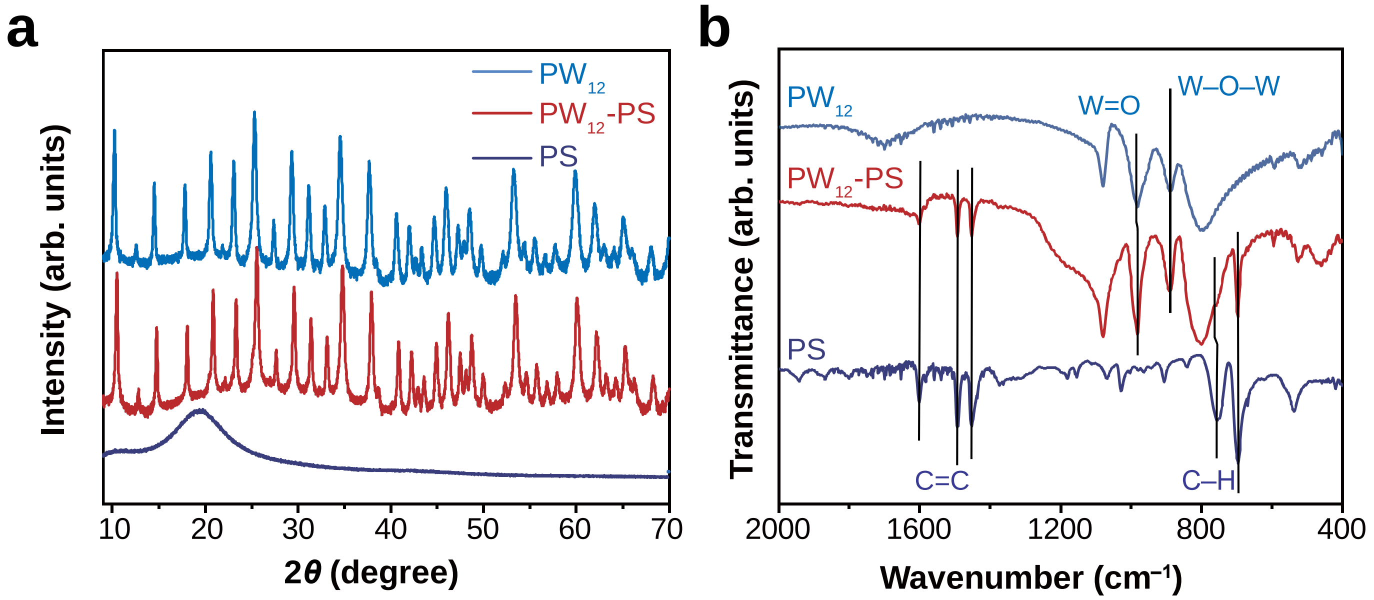

Supplementary Fig. 15 | Characterization of PW_{12}-PS extension composite. a, XRD and **b**, FTIR spectra of PW_{12}, PS, and PW_{12}-PS. Source data are provided as a Source Data file.

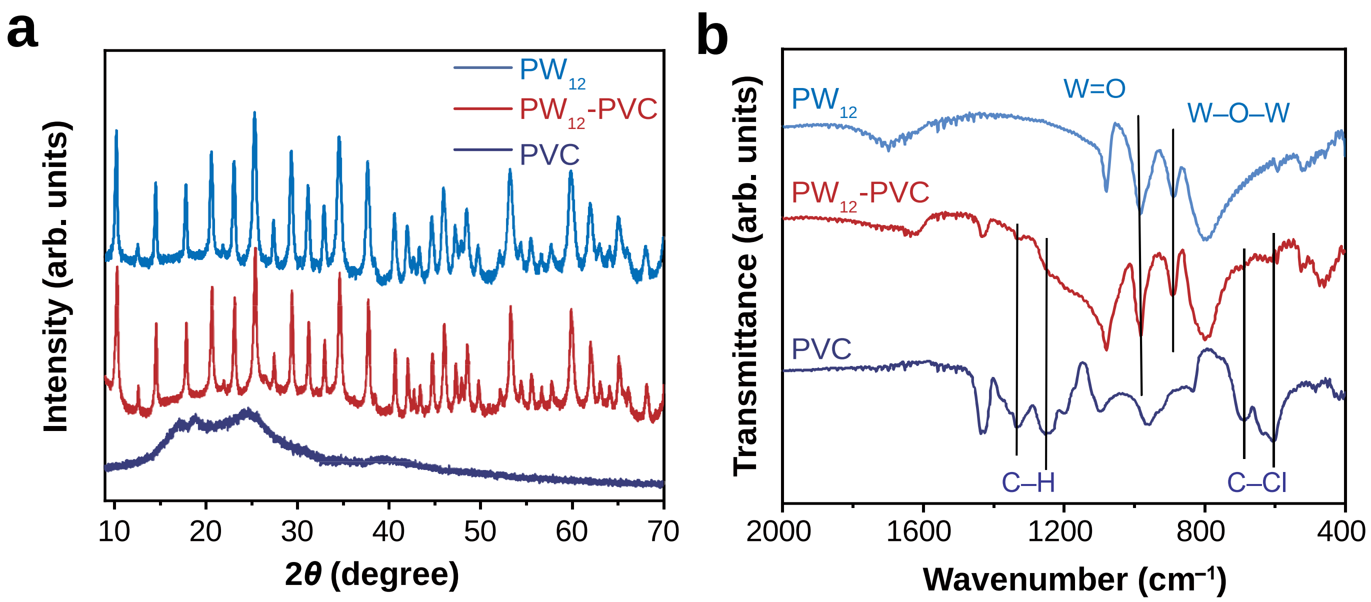

Supplementary Fig. 16 | Characterization of PW_{12}-PVC extension composite. a, XRD and **b**, FTIR spectra of PW_{12}, PVC, and PW_{12}-PVC. Source data are provided as a Source Data file.

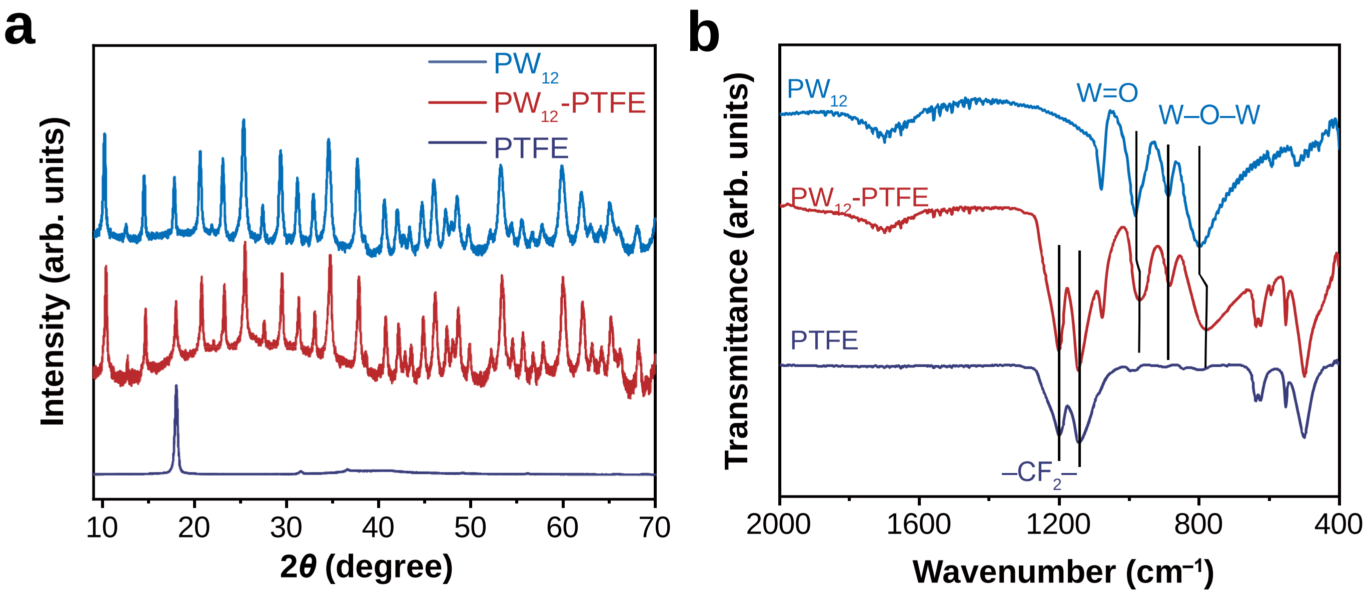

Supplementary Fig. 17 | Characterization of PW_{12}-PTFE extension composite. **a**, XRD and **b**, FTIR spectra of PW_{12}, PTFE, and PW_{12}-PTFE. Source data are provided as a Source Data file.

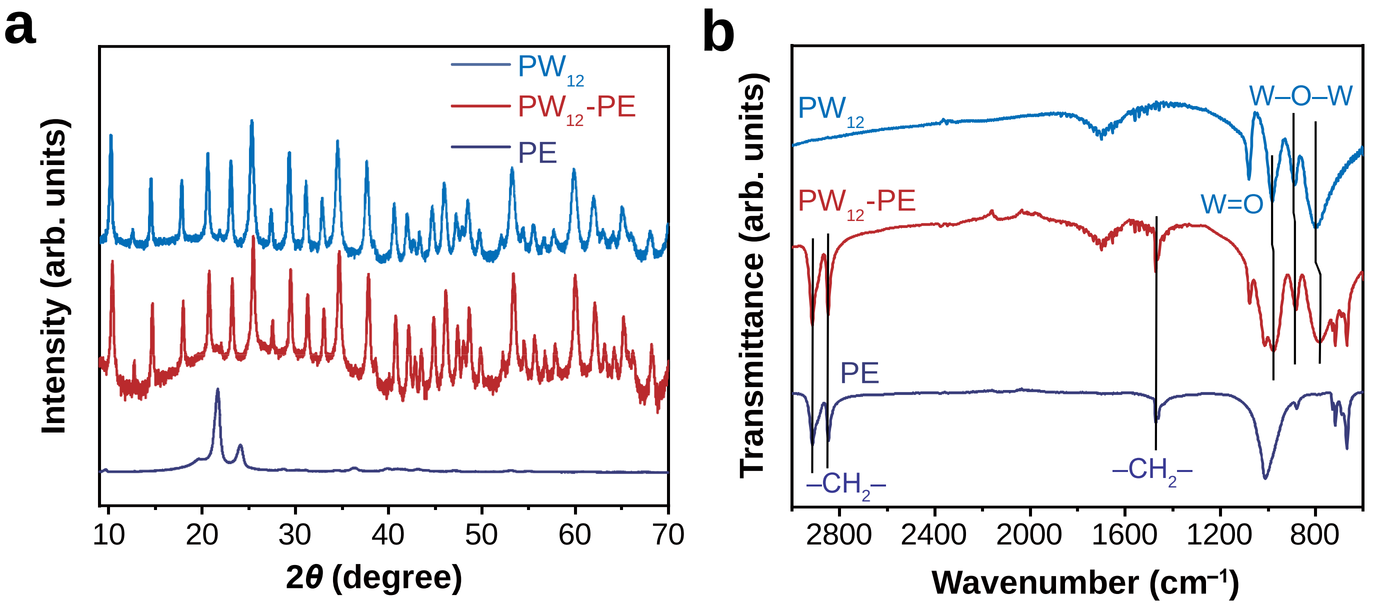

Supplementary Fig. 18 | Characterization of PW_{12}-PE extension composite. a, XRD and **b**, FTIR spectra of PW_{12}, PE, and PW_{12}-PE. Source data are provided as a Source Data file.

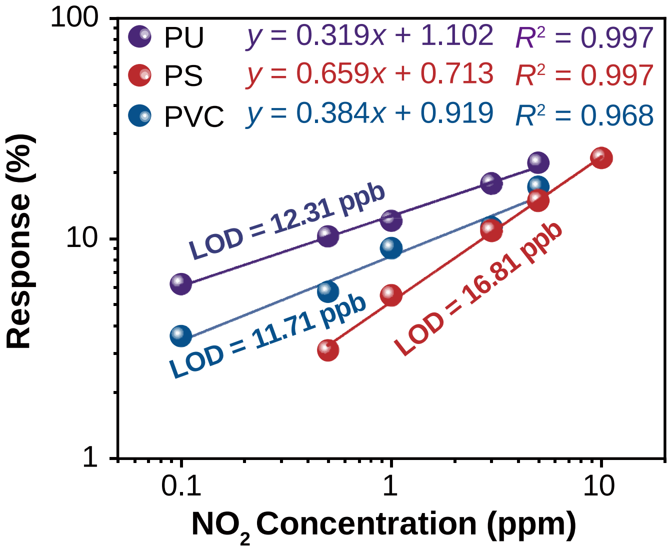

Supplementary Fig. 19 | NO_2 sensing performance of PW_{12}-plastic in the low concentration range of 0.1–10 ppm. The response value of PW_{12}-PU, PW_{12}-PS and PW_{12}-PVC composites have a log-log linear relationship with the NO_2 concentration. The solid line represents a linear fit. All experiments represent the standard deviation of at least three independent measurements. Data are shown as representative results from at least three independent experiments. Source data are provided as a Source Data file.

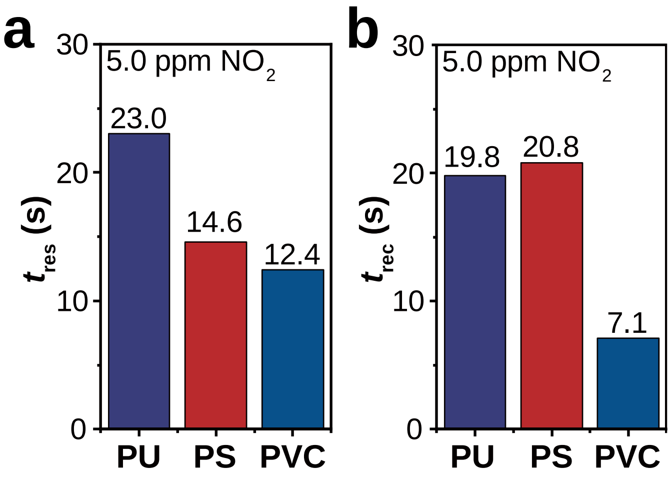

Supplementary Fig. 20 | Comparison of t_{res} and t_{rec} for three extended PW_{12}-plastic composites. a, t_{res} and **b**, t_{rec} of PW_{12}-PU, PW_{12}-PS, and PW_{12}-PVC at 5.0 ppm NO_2. Data are shown as representative results from at least three independent experiments. Source data are provided as a Source Data file.

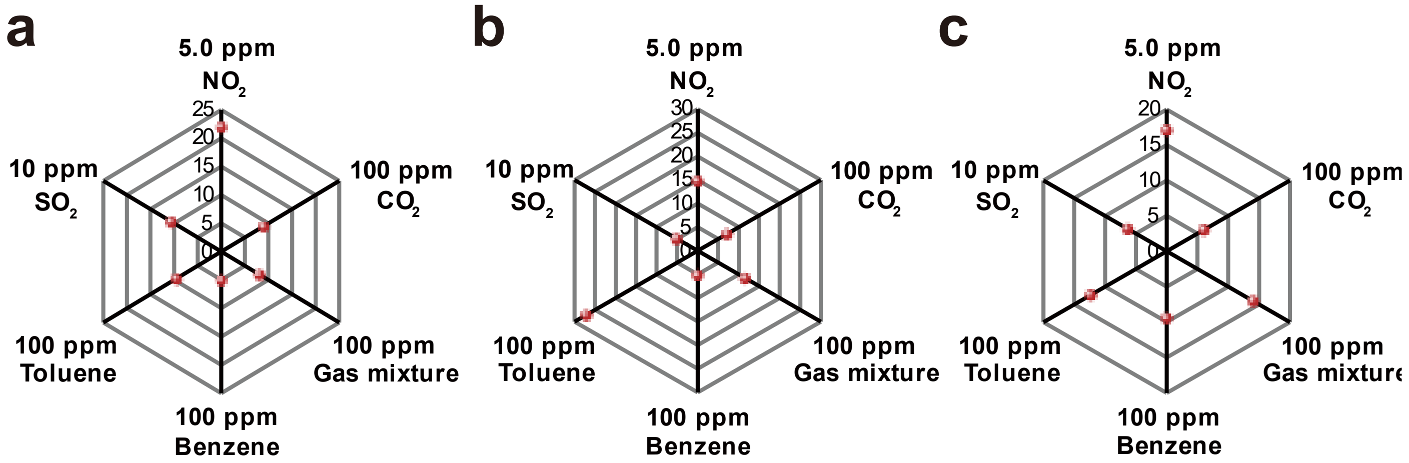

Supplementary Fig. 21 | Selectivity of three extended PW_{12}-plastics. Responses of **a**, PW_{12}-PU, **b**, PW_{12}-PS, and **c**, PW_{12}-PVC sensors to various gases [inset: the gas mixture includes CH_4 (25.63%), C_2H_4 (7.58%), C_2H_6 (7.41%), C_3H_8 (5.05%), C_4H_{10} (5.12%), H_2 (49.22%)]. CO_2, SO_2 and gas mixture are tested directly using standard gases, benzene and toluene are tested after being vaporized into gases. Data are shown as representative results from at least three independent experiments. Source data are provided as a Source Data file.

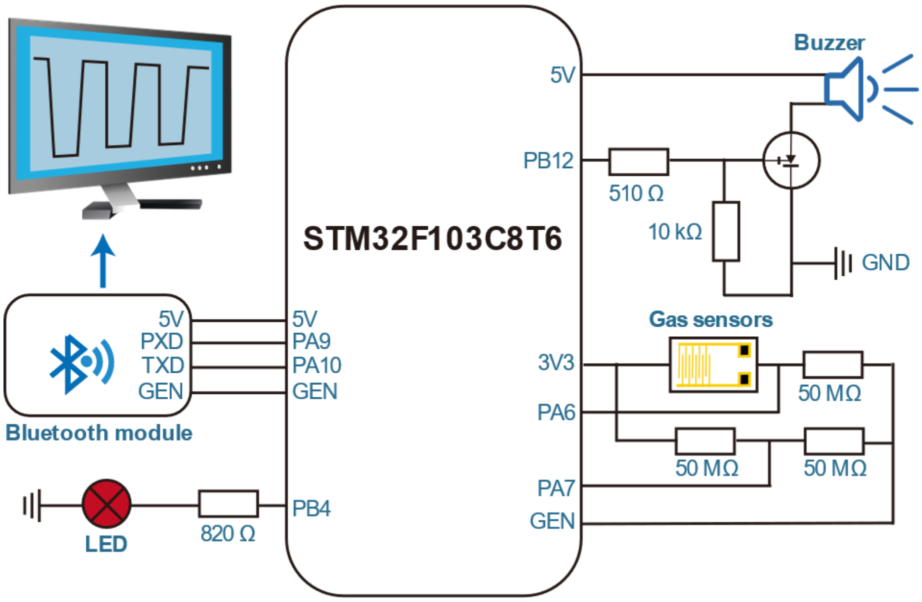

Supplementary Fig. 22 | Schematic diagram of the smart sensing device. Illustration of the integrated smart device based on the PW_{12}-PET-1 composite. The system comprises a power module, sensor module, STM32C8T6 microcontroller, and a low-power Bluetooth module, enabling functions such as signal acquisition, conditioning, processing, and wireless transmission. When the NO_2 concentration exceeds 5.0 ppm, the LED indicator will light up and emit an alarm signal in real time.

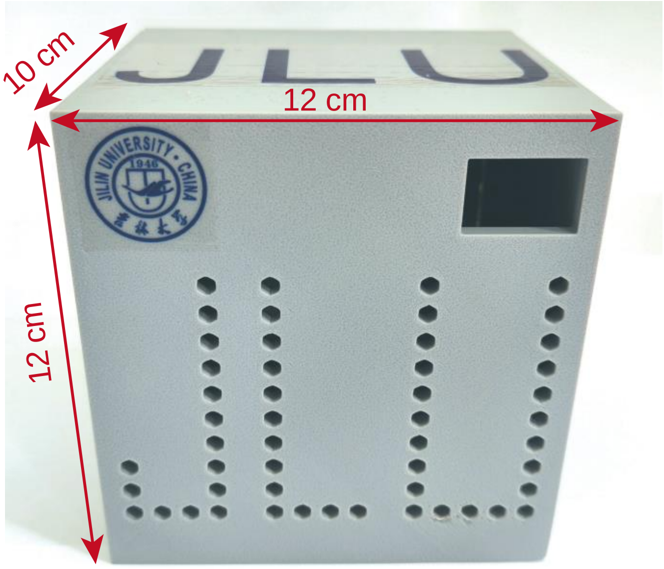

Supplementary Fig. 23 | Physical picture of the sensor box. The box of the sensing device was manufactured using 3D printing technology.

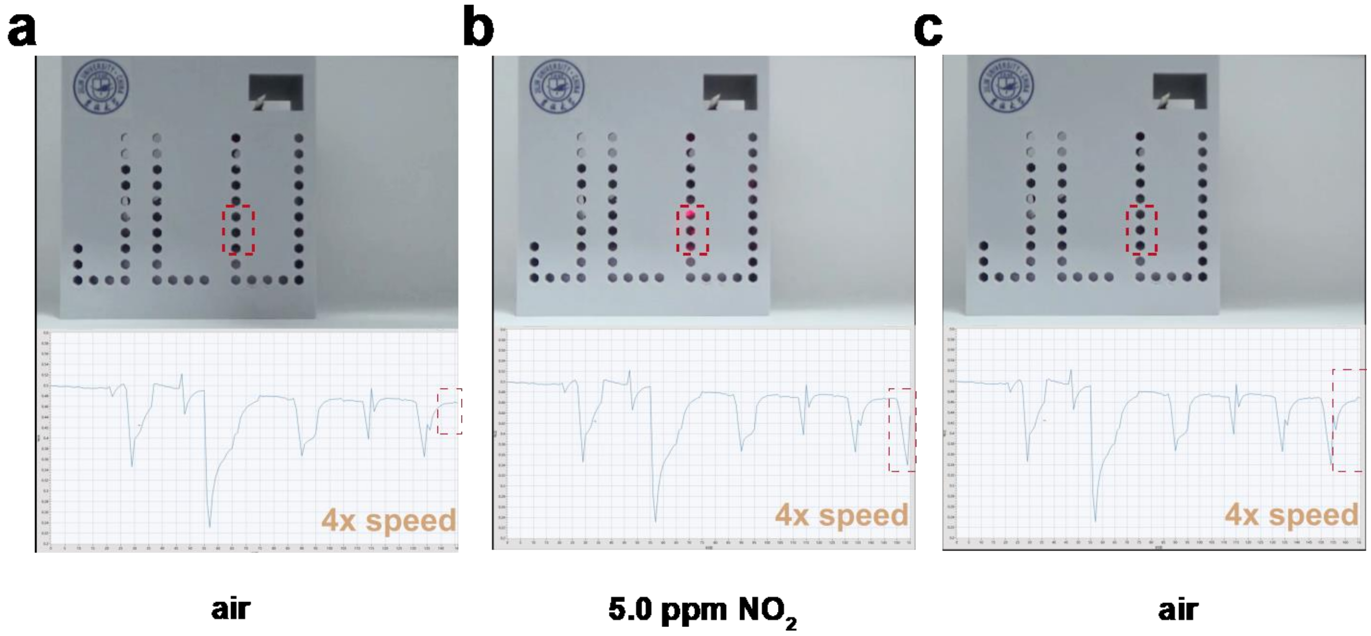

Supplementary Fig. 24 | Real-time monitoring of the NO_2. a, Under ambient air conditions, the current remains stable, and the LED indicator remains off. **b**, When the NO_2 concentration exceeds 5.0 ppm, a noticeable change in current is observed, and the LED light gives an alarm. **c**, When the NO_2 concentration decreases, it will rapidly revert to its state in air.

Supplementary Tables

Supplementary Table 1 | FTIR characteristic absorption bands. Assignments of the major PET and PW_{12} FTIR bands

PET		PW_{12}	
1717 cm^{-1}	Carbonyl (C=O) group stretching vibrations (amorphous)	1080 cm^{-1}	P–O
1508 cm^{-1}	Ring stretching vibrations	980 cm^{-1}	W=O
1457 cm^{-1}	Methylene (–CH_2) group bending vibrations	890, 810 cm^{-1}	W–O–W
1407 cm^{-1}	Aromatic skeleton stretching vibrations		
1338 cm^{-1}	Amorphous glycol (CH_2) group wagging vibrations		
1239 cm^{-1}	Amorphous ester group stretching		
1175, 1118, 1091 cm^{-1}	Glycol (CH_2) group conformational changes		
1017 cm^{-1}	In-plane bending vibrations of C–H bond		
971, 874, 848 cm^{-1}	In-plane bending vibrations of glycol (CH_2) group		
725 cm^{-1}	Out-of-plane bending vibrations of C–H bond		

Supplementary Table 2 | Performance Comparison with other Chemoreceptive Materials. Comparison of the sensing performance of various materials toward NO_2 at room temperature (25 °C), as reported in the literature

Materials	Auxiliary method	t_{res}/t_{rec} (s)	LOD (ppb)	Res. %/ Con. (ppm)	Humidity (%)	Repeatability (day)	Ref.
Au-CeO_2	photo-thermal-activated	17/15	15	20/10	5–70	18	[1]
Au-$Bi_xIn_{2-x}O_3$	light-activated	348/300	100	64.9/0.4	0–50	–	[2]
MoS_2 p-n junction	UV radiation	150/30	8	50/1	–	180	[3]
TiO_2@NH_2-MIL-125	520 nm light	16.8/78	1	207/100	–	365	[4]
HIOTP-Ni	–	101.4/619.2	210	405/10	–	–	[5]
$Mo_2TiC_2T_x$/MoS_2	–	34.8/140.5	2.5	415.8/50	0–90	14	[6]
VO_x/LIG	–	217/650	0.45	2.5/1	50–80	16	[7]
CdS QDs	–	29/28	11	17.6/1.76	50	–	[8]
Al-ZnO	–	360/180	200	1.4/0.5	10–75	60	[9]
C-MoS_2	–	43.1/301.2	130	2500/10	–	–	[10]
PW_{12}-PET-1	–	23.2/1.8	10.52	2588/100	10–54	21	(This work)
	–	19.2/13.5		30/5.0			

Supplementary References

1. Ou, Y. *et al*. Tandem electric-fields prolong energetic hot electrons lifetime for ultra-fast and stable NO_2 detection. *Adv. Mater.* **36**, 2403215 (2024).
2. Park, S. *et al*. Dual-photosensitizer synergy empowers ambient light photoactivation of indium oxide for high-performance NO_2 sensing. *Adv. Mater.* **36**, 2313731 (2024).
3. Zheng, W. *et al*. MoS_2 van der Waals p-n junctions enabling highly selective room-temperature NO_2 sensor. *Adv. Funct. Mater.* **30**, 2000435 (2020).
4. Deng, W.-H., Zhang, M.-Y., Li, C.-S., Yao, M.-S.&Xu, G. Energy-level alignment at TiO_2@NH_2-MIL-125 interface for high-performance gas sensing. *Angew. Chem. Int. Ed.* **64**, e202419195 (2025).
5. Chen, P. *et al*. Two-dimensional conjugated metal-organic frameworks with large pore apertures and high surface areas for NO_2 selective chemiresistive sensing. *Angew. Chem. Int. Ed.* **62**, e202306224 (2023).
6. Zhao, Q. *et al*. Edge-enriched Mo_2TiC_2Tx/MoS_2 heterostructure with coupling interface for selective NO_2 monitoring. *Adv. Funct. Mater.* **32**, 2203528 (2022).
7. Yang, L. *et al*. Vanadium oxide-doped laser-induced graphene multi-parameter sensor to decouple soil nitrogen loss and temperature. *Adv. Mater.* **35**, 2210322 (2023).
8. Hewa-Rahinduwage, C.C. *et al*. Reversible electrochemical gelation of metal chalcogenide quantum dots. *J. Am. Chem. Soc.* **142**, 12207–12215 (2020).
9. Sanger, A. *et al*. Morphology-controlled aluminum-doped zinc oxide nanofibers for highly sensitive NO_2 sensors with full recovery at room temperature. *Adv. Sci.* **5**, 1800816 (2018).
10. Kim, J. *et al*. Synergetic phase modulation and n-doping of MoS_2 for highly sensitive flexible NO_2 sensors. *Adv. Sci.* **12**, 2410825 (2025).
