# Supplementary material for: Mechanochemically functionalized waste plastics for NO2 sensing
Source: Nat Commun. 2026 Apr 25;17:5745. doi: 10.1038/s41467-026-72492-8 (PMC13324026; doi:10.1038/s41467-026-72492-8)
Supplement: Supplementary file 2 — Description of Additional Supplementary File [file 41467_2026_72492_MOESM2_ESM.pdf]

The Description of Additional Supplementary Files

Supplementary Data 1: This file includes all the atomic coordinates of the optimized computational models included in Figs. 4c–4f and Supplementary Figs. 3, 10–13.

Supplementary Movie 1: PW12-PET-1 smart device for real-time NO_2 monitoring. This video demonstrates the response and recovery process of the sensor, highlighting its functional reliability. When NO_2 concentration surpasses 5.0 ppm, the system will trigger a real-time LED and buzzer alarm.
