# Supplementary figures and images for: Mechanochemically functionalized waste plastics for NO2 sensing
Source: Nat Commun. 2026 Apr 25;17:5745. doi: 10.1038/s41467-026-72492-8 (PMC13324026; doi:10.1038/s41467-026-72492-8)

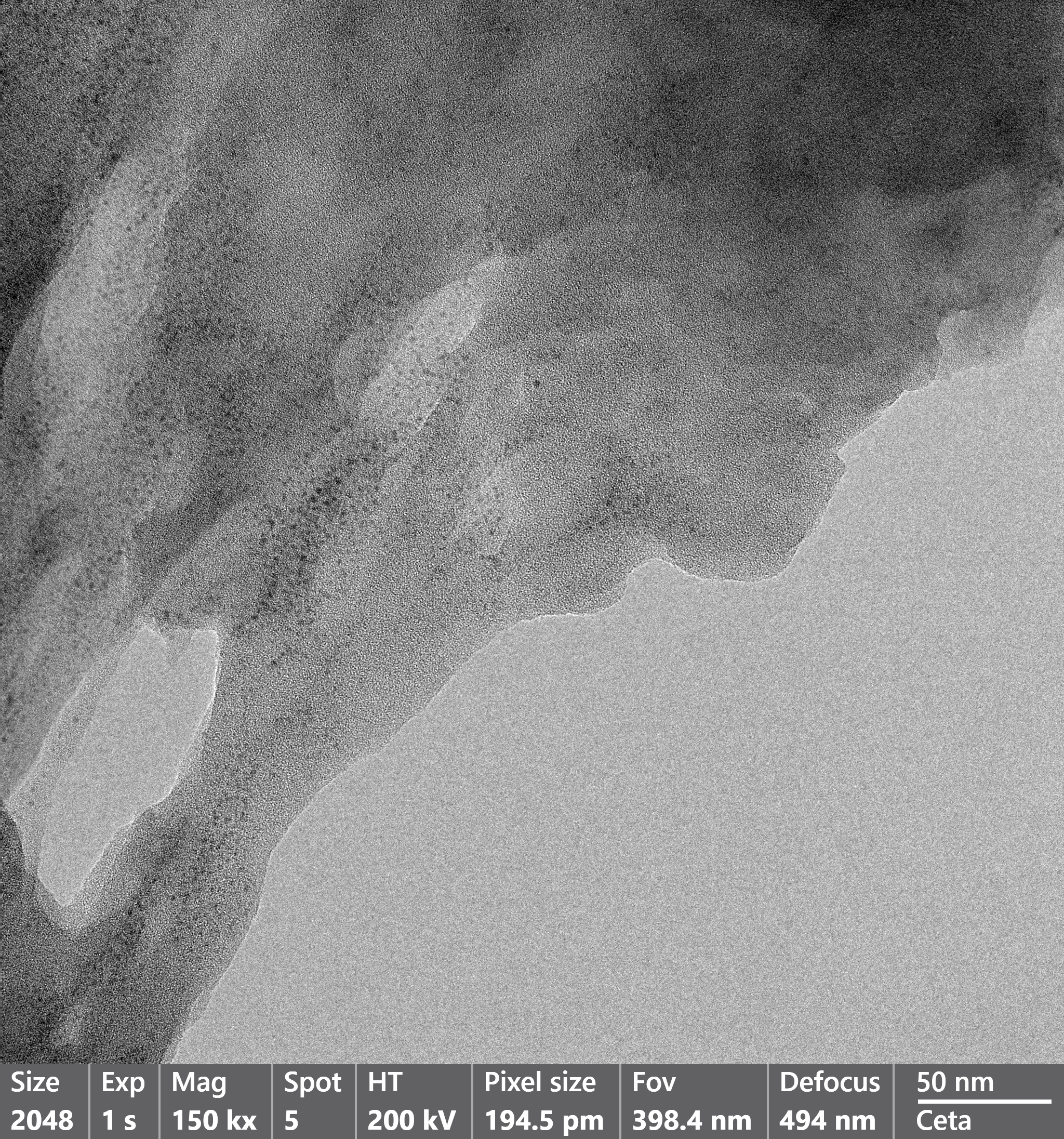

Supplement: Supplementary file 7 — Source Data [file 41467_2026_72492_MOESM7_ESM.zip › Source Data/Figure 2a,b.tif]
