# Supplementary material for: Mechanochemically functionalized waste plastics for NO2 sensing
Source: Nat Commun. 2026 Apr 25;17:5745. doi: 10.1038/s41467-026-72492-8 (PMC13324026; doi:10.1038/s41467-026-72492-8)
Supplement: Supplementary file 6 — Transparent Peer Review file [file 41467_2026_72492_MOESM6_ESM.pdf]

nature portfolio

Peer Review File

Mechanochemically functionalized waste plastics for NO2 sensing

Corresponding Author: Professor Gao-Feng Han

This file contains all reviewer reports in order by version, followed by all author rebuttals in order by version.

Version 0:

Reviewer comments:

Reviewer #1

(Remarks to the Author)
In this work, the authors present a mechanochemical approach for the functionalization of plastics with polyoxometalate (POM) molecules, which exhibits a fast response-recovery times to NO2. Besides, the charge transfer between the material and NO2 was explored by DFT analysis. However, the analysis of the NO2 sensing mechanism is insufficient, and significant issues remain unresolved. I do not recommend publishing this article in its current form in Nature Communications.
1. The author repeatedly emphasizes that the detection limit of this material for NO2 is lower than most sensors currently reported. However, upon careful analysis of the calculation formula for the detection limit, it seems that the results in the manuscript cannot be obtained. The calculated result may be at the ppm level, which does not achieve the superior performance described by the author. If there is a calculation error and the actual detection limit in the manuscript is 100 ppb, which is lower than the theoretical detection limit, how should the author explain it.
2. If the detection limit calculation is incorrect, the material does not show significant advantages compared to reported sensors.
3. Merely explaining electron transfer through DFT, XPS, and Raman analysis seems insufficient, as it is an electronic mechanism within the material. The author did not explore the sensing mechanism for detecting NO2.
4. In the page of 68-69 “but also increases lubricity and prevents the fracture of polymer chains.” The author should provide more characterization and experiments to confirm this statement.
5. The effect of humidity on the NO2 performance of materials should be explored, especially for polymer materials with abundant functional groups on their surfaces.
6. In the Introduction section, the author did not clearly describe the advantages of POM and the motivation for choosing POM.
7. Dynamic charge transfer between NO2 and material surfaces, as well as the adsorption and desorption processes of NO2, should be investigated.

Reviewer #2

(Remarks to the Author)
The manuscript titled “Mechanochemically functionalized waste plastics for NO2 sensing” reports on a mechanochemical method for directly functionalizing plastics with polyoxyethylene ester (POM) molecules. The research work is innovative. The experimental results largely support the conclusions drawn. However, prior to formal publication, a few issues require attention and revision.

Comment 1:
In the introduction, the author emphasizes the impacts of waste plastics, seemingly overemphasizing the significance of waste recycling while neglecting the importance of the sensing material itself. The logic and rigor in developing the PW12-PET sensing material are lacking. Additionally, the author mentions “The optimized PW12-PET-1 sample achieves a low limit of detection (LOD) 54 of 2.76 ppb, along with fast response and recovery times of 19.2 s and 13.5 s at 5.0 ppm NO2, respectively. It outperforms reported NO2 sensors.” The author’s introduction presents a one-sided view of the sensor’s performance. Can these few points alone demonstrate superiority over the reported NO2 sensors? For gas sensors, characteristics such as response values, moisture resistance and good repeatability are all factors that need to be considered.

Comment 2:
As mentioned above, the role of waste plastics appears to be overstated. The author should focus on elaborating the significance of using plastics in combination with polyoxometalates as sensing materials. After all, polyoxometalates have been widely applied as gas sensing materials.
Comment 3:
Regarding the microstructure of the composite materials, the manuscript provides only one TEM image, and the authors' stated findings cannot be clearly discerned from it. Additional evidence is required.
Comment 4:
The theoretical analysis in the manuscript are detailed and thorough, and PDOS is provided as supplementary material. However, the gas sensing test section is relatively weak. In Figure 3e, the sensor's resistance in NO2 did not reach a stable value.
Comment 5:
For NO2 gas detection, humidity testing is crucial. Supplement the sensor with test curves for NO2 at different humidity levels.

Reviewer #3

(Remarks to the Author)
The authors demonstrate a mechanochemically synthesized sensor for NO_2 detection, which exhibits a relatively low limit of detection (LOD) and a fast response rate. Upon addressing the following points, the manuscript would be suitable for publication in Nature Communications:
1. In lines 67–69, the authors state: "Water not only promotes complete dissolution of PW_{12}, facilitating proton activation and molecular-level functionalization of PET by polyoxoanions, but also increases lubricity and prevents the fracture of polymer chains." This claim should be supported by a relevant reference to strengthen its validity.
2. The field of view in Figure 2a is too large to clearly distinguish the PW_{12} nanoparticles. It is recommended that the authors either magnify the image or include an inset showing a higher-magnification region to better visualize the PW_{12} particles.
3. Regarding the O 1s XPS spectra in Fig. 2d, the signals for both PW_{12} and PW_{12}-PET-1 appear as a broad envelope at approximately 534-536 eV. The deconvolution into three, or even five, closely spaced peaks and the subsequent reliance on the subtle shifts of these deconvoluted peaks to demonstrate charge transfer lack sufficient rigor. The presented O 1s data may not be robust evidence. It might be preferable to remove Fig. 2d, as the C 1s spectra in Fig. 2e, which show more distinct peak shifts, provide adequate and clearer XPS evidence for the charge transfer.
4. In lines 263–264, the authors note that all measurements were conducted under laboratory conditions at 25 °C. It would be important to evaluate the influence of temperature and humidity on the NO_2 sensing performance of PW_{12}-PET to assess its practical applicability.
5. Table 1 and Supplementary Table 2 largely present overlapping information. Merging them into a single comprehensive table would improve clarity and avoid redundancy.
6. While the study effectively demonstrates the principle of functionalization using reagent-grade PET, a more compelling case for the "upcycling of waste plastics" could be made by validating the method with real post-consumer waste. It is therefore recommended that the authors synthesize PW_{12}-PET from real-life PET waste like discarded bottles or fibers as sources and compare its sensing performance to the baseline material. This direct comparison is crucial for bridging the gap between a laboratory proof-of-concept and a genuine waste upcycling strategy.

Version 1:

Reviewer comments:

Reviewer #1

(Remarks to the Author)
The author provided detailed answers to the previous questions. However, some concerns remain unresolved.
Although the impact of humidity on sensors has been extensively studied, this has been limited to the aspect of response. Authors should conduct a comprehensive study on the impact of humidity. For instance, the impact of different humidity levels on the baseline should be investigated. In the existing data, only changes after the injection of target gas are observed, and the baseline characteristics before the gas injection are missing. Changes in the baseline can affect the performance of sensor, such as cycle stability and long-term stability, etc.

Reviewer #2

(Remarks to the Author)
The revised version is acceptable and the issues raised have been resolved.

Reviewer #3

(Remarks to the Author)
All reviewer comments have been clearly responded to, and the corresponding revisions have enhanced the quality of the manuscript. We consider the paper now ready for publication in Nature Communications in its present form.

Version 2:

Reviewer comments:

Reviewer #1

(Remarks to the Author)
The manuscript was well revised, and it could be accepted for publication now.

Open Access This Peer Review File is licensed under a Creative Commons Attribution 4.0 International License, which permits use, sharing, adaptation, distribution and reproduction in any medium or format, as long as you give appropriate credit to the original author(s) and the source, provide a link to the Creative Commons license, and indicate if changes were made.
In cases where reviewers are anonymous, credit should be given to 'Anonymous Referee' and the source.
The images or other third party material in this Peer Review File are included in the article's Creative Commons license, unless indicated otherwise in a credit line to the material. If material is not included in the article's Creative Commons license and your intended use is not permitted by statutory regulation or exceeds the permitted use, you will need to obtain permission directly from the copyright holder.
To view a copy of this license, visit https://creativecommons.org/licenses/by/4.0/

Response to the Reviewers

Gao-Feng Han (On the behalf of all authors, who contributed to this work).

As a whole, the reviewers′ comments were very helpful to improve the quality of the manuscript. We are very grateful to the reviewers for their time and valuable comments.

Reviewer #1: (Remarks to the authors)

In this work, the authors present a mechanochemical approach for the functionalization of plastics with polyoxometalate (POM) molecules, which exhibits a fast response-recovery times to NO_2. Besides, the charge transfer between the material and NO_2 was explored by DFT analysis. However, the analysis of the NO_2 sensing mechanism is insufficient, and significant issues remain unresolved. I do not recommend publishing this article in its current form in Nature Communications.

Response #1: We sincerely thank Reviewer #1 for his/her careful evaluation of our work and the valuable constructive comments, which have undoubtedly helped us to further improve the quality and clarity of the manuscript. In response to these comments, we have carefully revised the paper and provided detailed explanations and additional analyses as outlined below. We hope that the revised version will meet with the reviewer's approval.

Comment 1.1: *The author repeatedly emphasizes that the detection limit of this material for NO_2 is lower than most sensors currently reported. However, upon careful analysis of the calculation formula for the detection limit, it seems that the results in the manuscript cannot be obtained. The calculated result may be at the ppm level, which does not achieve the superior performance described by the author. If there is a calculation error and the actual detection limit in the manuscript is 100 ppb, which is lower than the theoretical detection limit, how should the author explain it.*

Response 1.1: Many thanks for the reviewer's careful reading. Upon careful re-examination of our data, we found that we mistakenly used the absolute current values rather than the response values to calculate the root-mean-square deviation from the baseline. We sincerely appreciate the reviewer pointing out our carelessness, which has enabled us to identify and correct this error.

Accordingly, we have recalculated the limit of detection (LOD) values, and the detailed calculation procedure is provided below:

Step I: Fifty data points, collected at one-second intervals from the blank baseline, were extracted, converted into response values of background noise, and plotted as a function of time, as shown in Fig. R1.

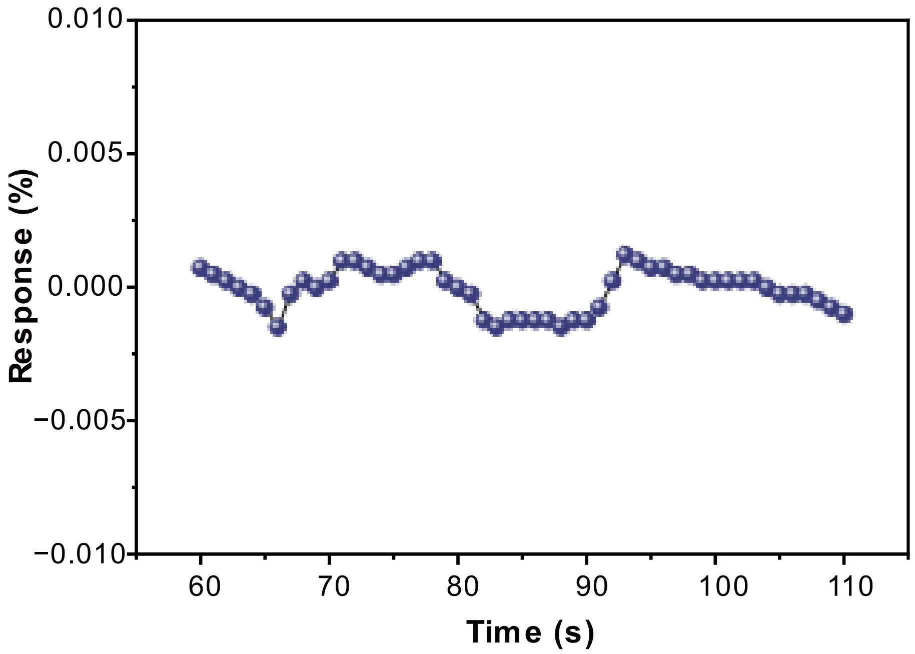

Fig. R1 | Blank background response of the PW_{12}-PET-1 sensor prior to NO_2 exposure.

Step II: Using Equations R1 and R2, the root-mean-square $\boldsymbol{RMS_{noise}}$ **was calculated to be 0.000782**.

$$V_{x^2} = \sum_{i=1}^{N} (Re_i - \overline{Re})^2 \qquad \text{(R1)}$$

$$RMS_{\text{noise}} = \sqrt{\frac{V_{x^2}}{N}} \qquad \text{(R2)}$$

Re_i represents the measured data point, $\overline{Re}$ is the mean of the blank background data, and N is the number of data points (50 data points were used in the curve fitting).

Step III: As the slope obtained from Fig. 3a is 0.223, according to Equation R3, **the LOD value was recalculated to be 10.52 ppb.**

$$LOD(\text{ppm}) = \frac{3 \times RMS_{\text{noise}}}{\text{slope}} \qquad \text{(R3)}$$

Subsequently, the errors related to the LOD values have been corrected throughout the Manuscript and Supplementary Information. In short, **the recalculated LOD values for PW_{12}-PET, PW_{12}-PU, PW_{12}-PS, and PW_{12}-PVC are 10.52, 12.31, 16.81, and 11.71 ppb, respectively.**

Comment 1.2: *If the detection limit calculation is incorrect, the material does not show significant advantages compared to reported sensors.*

Response 1.2: Many thanks for the reviewer's comment. As noted in Response 1.1, the recalculated LOD value of PW_{12}-PET-1 is **10.52 ppb**, which is just a bit higher than the value (2.76 ppb) we previously reported. Based on this value, we compared its response/recovery time, and LOD value with those reported in the literature. As shown in Fig. R2 (Fig. 3c in Manuscript) and Table R1 (Table 1 in Manuscript), **PW_{12}-PET-1 still demonstrates superior performance relative to previously reported NO_2 chemoreceptive materials and the other detection methods.**

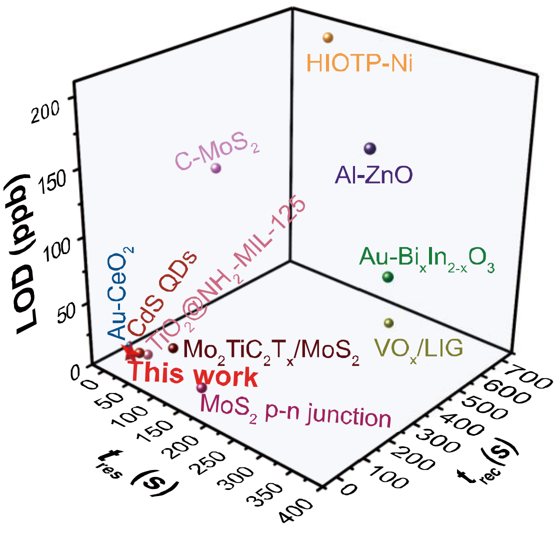

Fig. R2 | Comparison of t_{res}, t_{rec} and limit of detection (LOD) of PW_{12}-PET-1 at 5.0 ppm with previously reported chemoreceptive materials.

Table R1 | Compared with other detection methods. Comparison of the t_{res}, t_{rec} and LOD value of the PW_{12}-PET-1 chemoreceptive sensor with previous reported optical and field-effect transistor (FET)-based sensors for NO_2 detection

Sensor types	Materials	Auxiliary method	Concentrations (ppm)	t_{res} (s)	t_{rec} (s)	LOD (ppb)	Ref.
Optical	g-C_3N_4	-	5.0	30	120	3.2×10^5	[29]
	Cu-MOF-808	-	50	160	180	16	[30]
FET	CS-FETs	-	100	83	20	1000	[31]
	Pd/WS_2-FET	420 nm light	1.0	250	800	3.2	[32]
Chemoreceptive	**PW_{12}-PET-1**	-	**100**	**23.2**	**1.8**	**10.52**	**This work**
		-	**5.0**	**19.2**	**13.5**		

Comment 1.3: *Merely explaining electron transfer through DFT, XPS, and Raman analysis seems insufficient, as it is an electronic mechanism within the material. The author did not explore the sensing mechanism for detecting NO_2.*

Response 1.3: Thanks for the reviewer's thoughtful comment. To further elucidate the sensing mechanism for NO_2 detection, **we supplemented additional diffuse reflectance infrared Fourier transform spectra (DRIFTS) and in-situ Raman spectra measurements.**

As shown in Fig. R3a, the DRIFTS results reveal that NO_2 is effectively adsorbed on the PW_{12}-PET-1 surface, as evidenced by the characteristic NO_2 absorption band at 1621 cm^{-1}. Upon adsorption, NO_2 accepts electrons from PW_{12}-PET-1 and subsequently reacts with counter cations of H^+ to form NO and H_2O, which is supported by the appearance of corresponding peaks at 1894 cm^{-1} and 3582 cm^{-1}, attributable to NO and –OH, respectively[1–3]. Additionally, there is a strong $NO^{\delta+}$ signal observed at 2033 cm^{-1}, which is assigned to the stronger interaction between NO and PW_{12}-PET-1, leading to the formation of $[PW_{12}^{\delta-}\text{-}NO^{\delta+}]$ (Ref. 4). The key reactions involved in this process are summarized as follows (Equations R4 and R5):

$$NO_2 + 2e^- + 2H^+ \rightarrow NO + H_2O \quad \text{(R4)}$$

$$NO + PW_{12} \rightarrow [PW_{12}^{\delta-}\text{-}NO^{\delta+}] \quad \text{(R5)}$$

Additionally, in-situ Raman results (Fig. R3b) reveal that the bridge-oxygen of O_b1 on PW_{12}-PET-1 serves as the main active site for NO_2 adsorption. Upon NO_2 adsorption, the characteristic W–O–W vibrational peak gradually shifts towards higher wavenumbers, indicating electron transfer from the W–O–W in PW_{12}-PET-1 to NO_2. DFT calculations further confirm that O_b1 is indeed an important active site for NO_2 adsorption with adsorption energy of −0.52 eV and −0.42 eV on O_b1-1 and O_b1-2, respectively, accompanied by charge transfers of approximately 0.20*e* and 0.01*e* from PW_{12}-PET-1 to NO_2 (Fig. R3c,d).

Overall, by combining the DRIFTS, in-situ Raman and DFT results, the sensing mechanism for NO_2 detection is proposed: **During the ball-milling process of PET and PW_{12}, electron transfer occurs from PET to PW_{12}. Upon exposure to NO_2, NO_2 mainly adsorb on the bridge-oxygen active sites of O_b1 on PW_{12}-PET-1 surface. This process extracts electrons from PW_{12}-PET-1 and reacts with counter cations of H^+ to generate NO, H_2O and $[PW_{12}^{\delta-}\text{-}NO^{\delta+}]$ species, which are ultimately captured as the electrochemical sensing signal.**

According to these analyses, the following sentences were added in the Manuscript and the Methods: "To understand the dynamic surface reactions and charge transfer processes of PW_{12}-PET-1 during NO_2 sensing, in-situ diffuse reflectance infrared Fourier transform spectra (DRIFTS) and in-situ Raman measurements were performed. As shown in Fig.4a, upon exposure to NO_2, DRIFTS reveals that NO_2 is rapidly adsorbed on the PW_{12}-PET-1 surface, as evidenced by the characteristic NO_2 band observed at 1621 cm^{-1}. Following adsorption, NO_2 accepts electrons from the sensor

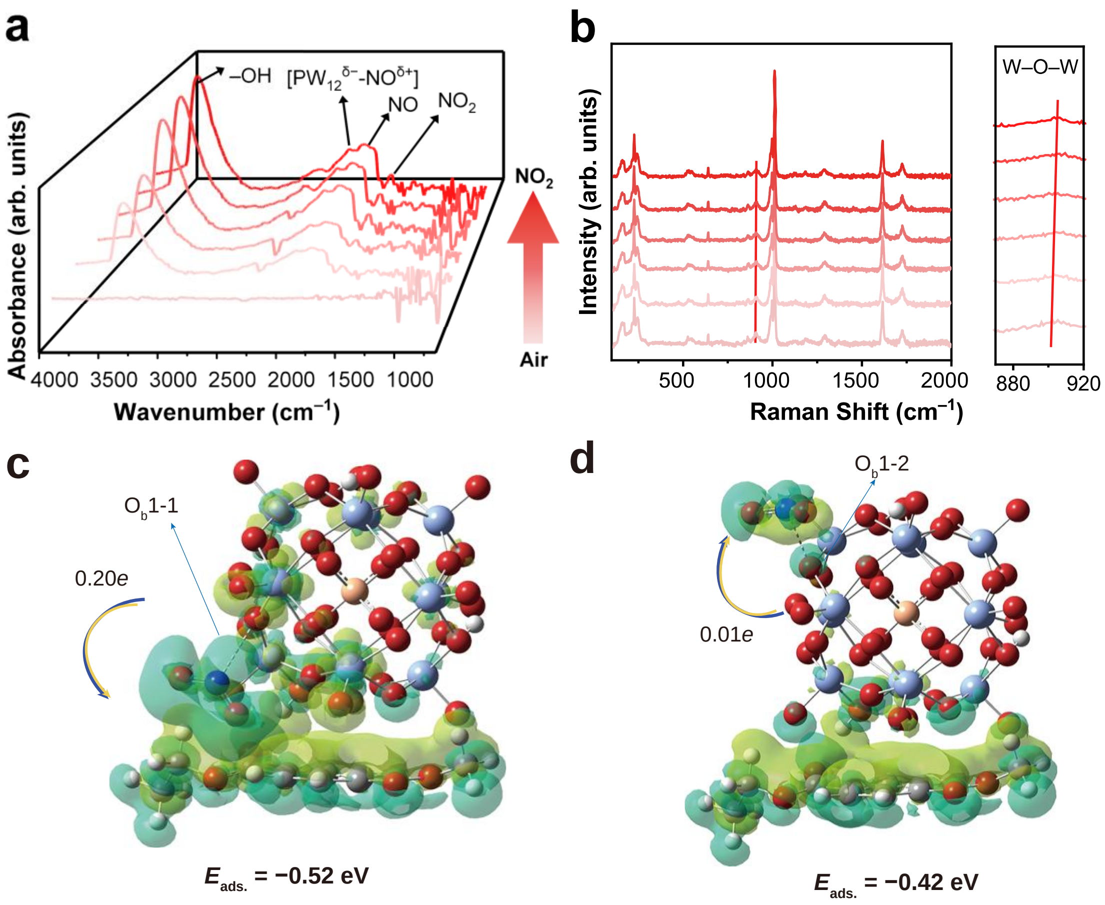

Fig. R3 | Mechanism of NO_2 detection on PW_{12}-PET-1. a, DRIFTS difference spectra recorded during the NO_2 sensing process of PW_{12}-PET-1 at room temperature. To obtain more obvious signal, the NO_2 concentration was set to 100 ppm. Upon continuous exposure to NO_2, the peaks corresponding to NO_2, NO, $NO^{\delta+}$ and –OH gradually increase, indicating the dynamic interaction between NO_2 and PW_{12}-PET-1. **b**, In-situ Raman spectra recorded during the NO_2 sensing process of PW_{12}-PET-1 at room temperature. During continuous adsorption, the characteristic W–O–W vibrational peak gradually shifts toward higher wavenumbers. **c**,**d**, Differential charge density distribution diagrams for NO_2 adsorption on the O_b1-1 (**c**, near the PET interface) and O_b1-2 (**d**, away from PET) bridge oxygen sites of 3*e* reduced PW_{12}. Here, O_b1 and O_b2 denote the corner-sharing and face-sharing bridging oxygen atoms, respectively, while O_t denotes the terminal oxygen atom. The grey-white, orange, blue, and red spheres represent H, P, W, and O atoms, respectively.

and reacts with the protons around adsorption sites to form NO and H_2O, as indicated by the appearance of peaks at 1894 cm^{-1} and 3582 cm^{-1}, respectively. Moreover, a strong signal at 2033 cm^{-1}, attributed to the $NO^{\delta+}$ intermediate appears, reflecting a strong interaction between NO and PW_{12}. Concurrently, in-situ Raman spectra (Fig. 4b) show that the W–O–W bridge-oxygen peak gradually shifts to higher wavenumbers

during NO_2 adsorption, providing direct evidence for electron transfer from PW_{12}-PET-1 to NO_2. Thus, the corresponding adsorption pathways can be summarized as follows:

$$NO_2+2e^{-}+2H^{+} \rightarrow NO+H_2O \quad (1)$$

$$NO+PW_{12} \rightarrow [PW_{12}^{\delta-}\text{-}NO^{\delta+}] \quad (2)$$"

and "The diffuse reflectance infrared Fourier transform spectra (DRIFTS) was performed on Nicolet iS10 (Thermo Fisher) spanning 600–4000 cm^{-1}, and controlled by OMINIC software. Raman and in-situ Raman spectra were recorded using a Renishaw micro-Raman spectrometer with a 532 nm excitation laser."

Comment 1.4: *In the page of 68-69 "but also increases lubricity and prevents the fracture of polymer chains." The author should provide more characterization and experiments to confirm this statement.*

Response 1.4: Thank you for the thoughtful suggestion. The previous explanation regarding the role of water was unscientific.

To clarify the role of water in the ball-milling process, we carried out a control experiment without the addition of water during ball milling (PW_{12}-PET-without water, Fig. R4a). As shown in Figs. R4b,c, it can be observed that, **in the absence of water, the samples are agglomerated and sticked to the bottom of the ball-mill jar due to high surface energy**. Conversely, the introduction of water during ball milling effectively prevented the sticking issue (Figs. R4d–4f).

Subsequently, X-ray diffraction (XRD) analysis (Fig. R4g) reveals that PW_{12} maintains its crystalline structure, while PET exhibits a weak and broad diffraction peak owing to the low crystallinity of pristine PET, consistent with the PW_{12}-PET-1. Fourier-transform infrared (FTIR) spectroscopic analysis (Fig. R4h) reveals that only a small fraction of PET in PW_{12}-PET-without water is effectively functionalized by PW_{12}. This is evidenced by the slight blue shifts of several characteristic vibrational bands of PET (C=O, C–O–C, and $–CH_2$), whereas the majority of the characteristic peaks still remain unchanged compared with those of pristine PET and PW_{12}. **These results demonstrate that PET cannot be effectively functionalized by PW_{12} in the absence of water.**

Therefore, water plays a crucial role in the molecular-level functionalization of PET by PW_{12}, as summarized below:

(1) It preventing agglomeration during ball milling;

(2) It promotes the complete dissolution of PW_{12};

(3) It enables proton activation and molecular-level functionalization of PET by PW_{12}.

In mechanochemical processes, small amounts of liquids (such as H_2O) are commonly added as lubricants[5,6]. However, in our ball-milling system operating at a low transmission ratio (1:−1) and a low rotation speed of 300 rpm, lubricity does not represent the primary role of water.

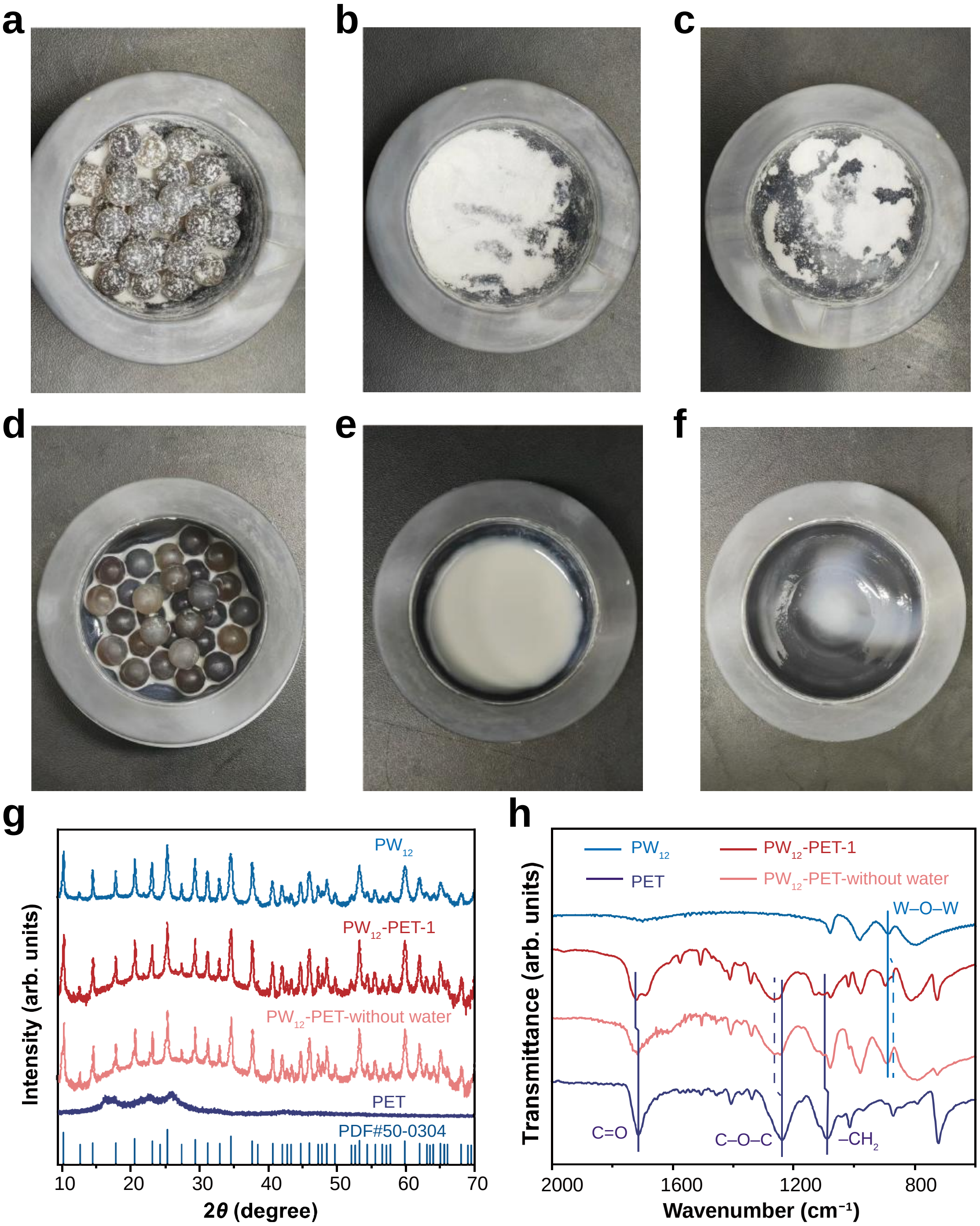

Fig. R4 | Comparison of samples under different ball-milling conditions. a–c, Optical images after ball milling without water. Part of the sample adheres to the surface of the agate ball (a). The sample condition after removing the agate ball shows that the most of the material remains in powder form (b), regions exhibit an agglomerated phenomenon (c). This compaction is speculated to be related to the crystalline water in PW_{12}. **d–f,** Optical images after ball milling with water, where no agglomeration was observed. **g**, XRD patterns of PW_{12}, PET, PW_{12}-PET-1 and PW_{12}-PET-without water. PW_{12}-PET-without water consisted of the amorphous PET phase with broad peaks and crystalline PW_{12} peaks (PDF#50-0304 corresponds to $PW_{12}\cdot 6H_2O$). **h**, FTIR spectra of PW_{12}, PET, PW_{12}-PET-1 and PW_{12}-PET-1 without water.

Accordingly, the description of the function of water in the Manuscript has been revised as follows: "In this process, water plays a crucial role. It prevents particle agglomeration and promotes complete dissolution of PW_{12}, thereby facilitating effective proton activation and molecular-level functionalization of PET by polyoxoanions."

Comment 1.5: *The effect of humidity on the NO_2 performance of materials should be explored, especially for polymer materials with abundant functional groups on their surfaces.*

Response 1.5: Thanks for the reviewer's constructive suggestion. In accordance with the reviewer's suggestion, we have conducted additional humidity-dependent sensing measurements at a fixed NO_2 concentration of 5.0 ppm, spanning a **relative humidity (RH) range of 10%–98%**.

As shown in Fig. R5a, within the 10%–98% RH range, the sensing response of PW_{12}-PET-1 first increases and then decreases, **reaching a maximum of approximately 40% at 34% RH**. Since our laboratory humidity generally ranges between 10% and 40% RH, the previously measured NO_2 response (30%) at ambient humidity falls within this range, confirming the reproducibility and reliability of the humidity-dependent results.

The observed humidity-dependent behavior can be explained as follows: Below 34% RH, increasing humidity facilitates charge transport within the PW_{12}-PET-1 composite, thereby enhancing the sensing response[7]. In contrast, when the humidity exceeds this threshold, excessive water molecules may competitively occupy the active adsorption sites, especially on the oxygen-rich surfaces of PW_{12}, which hinders NO_2 adsorption and reduces the sensing signal. Once the humidity exceeds 54% RH, the detection process becomes irreversible because the desorption of the analyte during the recovery process becomes extremely slow[8]. Thus, the current cannot return to its baseline after the recovery step. At extreme humidity (98% RH), the formation of a continuous water film on the surface leads to a short circuit (Fig. R5b), resulting in functional failure. This phenomenon is commonly observed in many analogous materials[9–12].

Following these discussions, the corresponding content has been added to the Manuscript and the Methods: "Similarly, the effect of relative humidity was also assessed (Fig. 3h and Supplementary Fig. 7b), with a peak response at 35% RH, as moderate humidity facilitates charge transport. Beyond this range, increased humidity slows recovery, and near 100% RH, short-circuiting may occur due to surface water film formation." and "Correspondingly, the relative humidity (RH) was independently adjusted (10%, 34%, 54%, 76%, and 98%) under a constant 5.0 ppm NO_2."

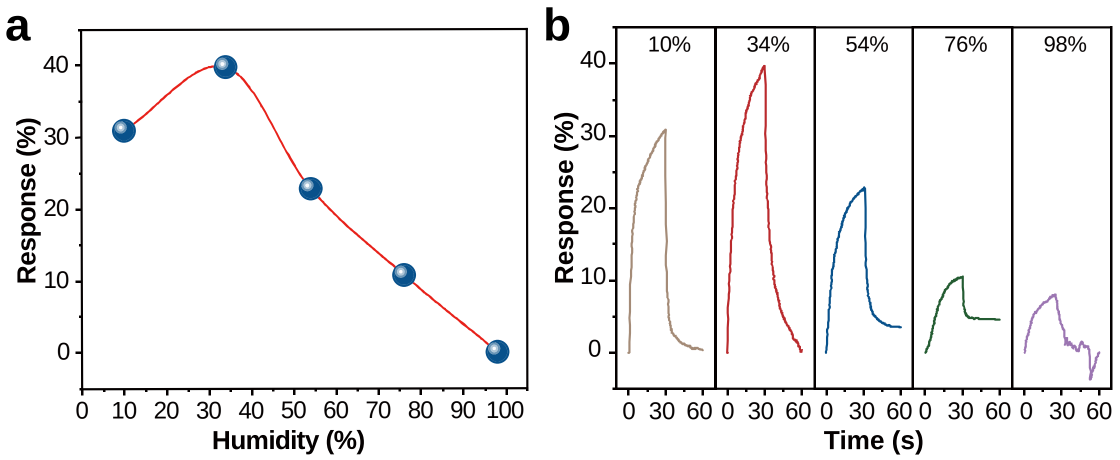

Fig. R5 | Humidity-dependent sensing performance of PW_{12}-PET-1 toward 5.0 ppm NO_2. **a**, Gas-sensing response and **b**, normalized response-recovery curves at various relative humidity (RH) levels (10%, 34%, 54%, 76%, and 98%).

Comment 1.6: *In the Introduction section, the author did not clearly describe the advantages of POM and the motivation for choosing POM.*

Response 1.6: Many thanks for the reviewer's meaningful comment. In our composite materials, the selection of polyoxometalates (POMs) as key components is driven by the two motivations as follows:

(1) POMs are environmentally friendly inorganic acids with low corrosivity[13]. Due to their **strong Brønsted acidity**, this allows them to act as **interfacial activators** that protonate and activate specific functional groups (such as C=O in esters) on inert plastic surfaces, ensuring stable chemical anchoring through a hydrogen-bonding and electrostatic network. The synergistic interaction ensures tight binding, **achieving functionalization of the plastic substrate at the molecular level** through a mechanochemical approach.

(2) POM anions offer unique advantages for gas sensing due to their **tunable redox properties at the molecular level**[14,15]. Specifically, their **ability to undergo reversible multi-electron transfer without structural degradation** significantly facilitates charge exchange between the sensing material and the target gas molecules.

Accordingly, we have revised the Introduction as follows: "In this context, polyoxometalates (POMs) provide a unique solution to this challenge. Specifically, their molecular-level tunability enables them to function as both interfacial activators and sensing centers. Their strong Brønsted acidity and low corrosivity enable the selective activation of specific functional groups (such as C=O in esters) on inert plastic surfaces, facilitating stable chemical anchoring through a synergistic network of hydrogen bonding and electrostatic interactions. Furthermore, the inherent ability of POM anions to undergo reversible multi-electron transfer without structural degradation significantly facilitates charge transfer during gas interaction. Crucially,

the plastic functions as a functionalized platform that modulates the electronic environment and charge kinetics of the POM active sites. This structure effectively addresses the inherent limitations of pure POMs (limited active site exposure), thereby transforming waste plastics into high-performance, active components for gas sensing."

Comment 1.7: *Dynamic charge transfer between NO_2 and material surfaces, as well as the adsorption and desorption processes of NO_2, should be investigated.*

Response 1.7: Thanks for the reviewer's thoughtful comment. Following the research discussed in Response 1.3, we conducted a more detailed study of the adsorption-desorption process of NO_2 through **a synergistic analysis of DRIFTS, in-situ Raman spectroscopy, and DFT calculations**. The details are provided as follows:

During the adsorption process, DRIFTS results (Fig. R6a) reveal that NO_2 is effectively adsorbed on the PW_{12}-PET-1 surface, as evidenced by the characteristic NO_2 absorption band at 1621 cm^{-1}. Upon adsorption, NO_2 accepts electrons from PW_{12}-PET-1, and reacts with the counter cations of H^+ to form NO and H_2O, which is supported by the appearance of corresponding peaks at 1894 cm^{-1} and 3582 cm^{-1}, attributable to NO and –OH, respectively[1–3]. Additionally, there is a strong $NO^{\delta+}$ signal observed at 2033 cm^{-1}, which is assigned to the stronger interaction between NO and PW_{12}-PET-1, leading to the formation of $[PW_{12}^{\delta-}\text{-}NO^{\delta+}]$[4]. The key reactions involved are summarized as follows (Equations R6 and R7):

$$NO_2 + 2e^- + 2H^+ \rightarrow NO + H_2O \qquad \text{(R6)}$$

$$NO + PW_{12} \rightarrow [PW_{12}^{\delta-}\text{-}NO^{\delta+}] \qquad \text{(R7)}$$

Additionally, in-situ Raman results (Fig. R6b) reveal that the bridge-oxygen of O_b1 on PW_{12}-PET-1 serves as the main active sites for NO_2 adsorption. Upon NO_2 adsorption, the characteristic W–O–W vibrational peak gradually shifts towards higher wavenumbers, indicating electron transfer from the W–O–W in PW_{12}-PET-1 to NO_2。 DFT calculations further confirm that O_b1 is indeed an important active site for NO_2 adsorption with adsorption energy of −0.52 eV and −0.42 eV on O_b1-1 and O_b1-2, respectively, accompanied by charge transfers of approximately 0.20*e* and 0.01*e* from PW_{12}-PET-1 to NO_2 (Fig. R6c,d).

Overall, by combining the DRIFTS, in-situ Raman and DFT results, the dynamic charge transfer and adsorption process of NO_2 were proposed: **During the ball-milling process of PET and PW_{12}, electron transfer occurs from PET to PW_{12}. Upon exposure to NO_2, NO_2 mainly adsorb on the bridge-oxygen active sites of O_b1 on PW_{12}-PET-1 surface. This process extracts electrons from PW_{12}-PET-1 and reacts with counter cations of H^+ to generate NO, H_2O and $[PW_{12}^{\delta-}\text{-}NO^{\delta+}]$ species, which are ultimately captured as the electrochemical sensing signal.**

During the NO_2 desorption process, DRIFTS spectra demonstrate the recovery behavior of PW_{12}-PET-1. This process exhibits a trend opposite to the adsorption stage,

indicating that the adsorption-desorption reaction is highly reversible, which ensures the stable cycling performance. As shown in Fig. R6a, when the atmosphere is switched to air, the NO_2 adsorption peak rapid decline. Subsequently, the $NO^{\delta+}$ signal weakens, indicating that the intermediate $[PW_{12}^{\delta-}\text{-}NO^{\delta+}]$ dissociates and releases NO. Meanwhile, the weakening of the NO and H_2O shows that NO and H_2O undergo the inverse reaction, regenerating NO_2 and H^+, ultimately restoring the initial state. The desorption reaction pathway can be summarized as follows (Equations R8 and R9):

$$[PW_{12}^{\delta-}\text{-}NO^{\delta+}] \rightarrow NO + PW_{12} \qquad \text{(R8)}$$

$$NO + H_2O \rightarrow NO_2 + 2e^- + 2H^+ \qquad \text{(R9)}$$

Concurrently, the corresponding in-situ Raman spectra (Fig. R6b) show that the W–O–W characteristic peak gradually shifts to lower wavenumbers and recovers to its initial state, indicating that both the electronic structure and the chemical environment of PW_{12}-PET-1 are restored.

Overall, the dynamic electron and proton recovery process can be summarized as follows: **During desorption, physically adsorbed NO_2 rapidly desorbs, followed by the dissociation of $[PW_{12}^{\delta-}\text{-}NO^{\delta+}]$ to release NO. Subsequently, NO undergoes a reverse reaction with H_2O, regenerating NO_2 and H^+ while transferring electrons and protons back to PW_{12}-PET-1.**

In summary, the entire adsorption-desorption process of NO_2 on PW_{12}-PET-1 exhibits good chemical reversibility. This stems from the counter cations of H^+ and the flexible electron donation/acceptance capability of PW_{12}-PET-1, which actively participate in the surface NO_2 adsorption-desorption reactions. These features are crucial for ensuring the long-term sensing stability of PW_{12}-PET-1.

Accordingly, we have revised the Manuscript and the Methods as follows: "To understand the dynamic surface reactions and charge transfer processes of PW_{12}-PET-1 during NO_2 sensing, in-situ diffuse reflectance infrared Fourier transform spectra (DRIFTS) and in-situ Raman measurements were performed. As shown in Fig.4a, upon exposure to NO_2, DRIFTS reveals that NO_2 is rapidly adsorbed on the PW_{12}-PET-1 surface, as evidenced by the characteristic NO_2 band observed at 1621 cm^{-1}. Following adsorption, NO_2 accepts electrons from the sensor and reacts with the protons around adsorption sites to form NO and H_2O, as indicated by the appearance of peaks at 1894 cm^{-1} and 3582 cm^{-1}, respectively[47–49]. Moreover, a strong signal at 2033 cm^{-1}, attributed to the $NO^{\delta+}$ intermediate appears[50], reflecting a strong interaction between NO and PW_{12}. Concurrently, in-situ Raman spectra (Fig. 4b) show that the W–O–W bridge-oxygen peak gradually shifts to higher wavenumbers during NO_2 adsorption, providing direct evidence for electron transfer from PW_{12}-PET-1 to NO_2. Thus, the corresponding adsorption pathways can be summarized as follows:

$$NO_2 + 2e^- + 2H^+ \rightarrow NO + H_2O \qquad (1)$$

$$NO + PW_{12} \rightarrow [PW_{12}^{\delta-}\text{-}NO^{\delta+}] \qquad (2)$$

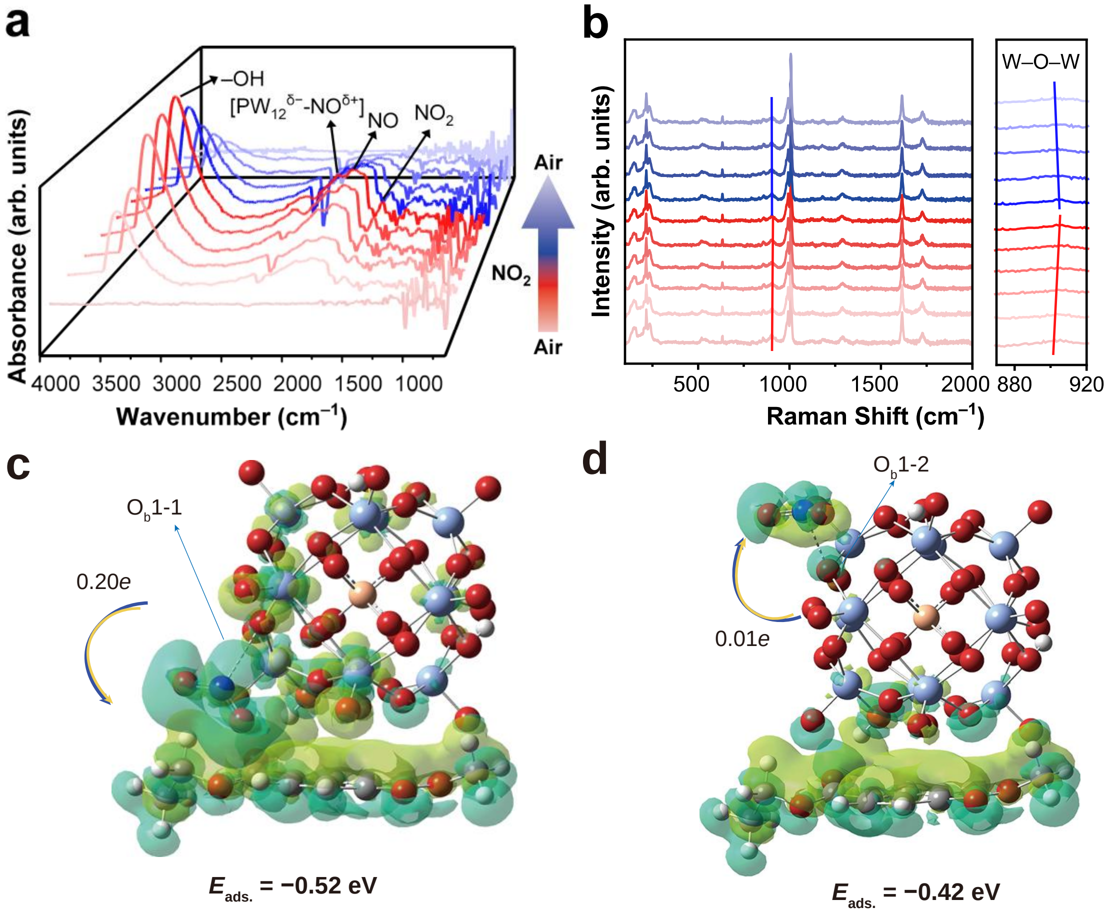

Fig. R6 | Mechanism of NO_2 adsorption and desorption processes of PW_{12}-PET-1. a, NO_2 adsorption-desorption DRIFTS difference spectra of PW_{12}-PET-1 at room temperature. To obtain more obvious signal, the concentration of NO_2 was used 100 ppm. Upon continuous exposure to NO_2, the peaks corresponding to NO_2, NO, $NO^{\delta+}$ and –OH gradually increase, indicating a dynamic interaction between NO_2 and PW_{12}-PET-1. After reaching a stable state, switching the NO_2 to air leads to desorption, restoring the spectra to their initial state. **b**, NO_2 adsorption-desorption in-situ Raman spectra of PW_{12}-PET-1 at room temperature. During continuous adsorption, the characteristic W–O–W vibrational peak gradually shifts toward higher wavenumbers. When exposed to air, the W–O–W peak recovers, shifting back toward its initial position. **c**,**d**, Differential charge density distribution diagrams for NO_2 adsorption on the O_b1-1 (**c**, near the PET interface) and O_b1-2 (**d**, away from PET) bridge oxygen sites of 3*e* reduced PW_{12}. Here, O_b1 and O_b2 denote the corner-sharing and face-sharing bridging oxygen atoms, respectively, while O_t denotes the terminal oxygen atom. The grey-white, orange, blue, and red spheres represent H, P, W, and O atoms, respectively.

Subsequently, the reversibility of PW_{12}-PET-1 was confirmed during the desorption process. Upon switching to air, the NO_2 adsorption peak and the intermediate signals in DRIFTS difference spectra rapidly declined, while the W–O–W peak in Raman returned to its initial position. This indicates that the adsorption-desorption reaction is

highly reversible, with NO and H_2O undergoing an inverse reaction to regenerate NO_2 and H^+, thereby restoring the initial electronic state.

$$[PW_{12}^{\delta-}\text{-}NO^{\delta+}] \rightarrow NO + PW_{12} \quad (3)$$

$$NO + H_2O \rightarrow NO_2 + 2e^- + 2H^+ \quad (4)$$"

and "The diffuse reflectance infrared Fourier transform spectra (DRIFTS) was performed on Nicolet iS10 (Thermo Fisher) spanning 600–4000 cm^{-1}, and controlled by OMINIC software. Raman and in-situ Raman spectra were recorded using a Renishaw micro-Raman spectrometer with a 532 nm excitation laser."

Reviewer #2: (Remarks to the authors)

The manuscript titled "Mechanochemically functionalized waste plastics for NO_2 sensing" reports on a mechanochemical method for directly functionalizing plastics with polyoxyethylene ester (POM) molecules. The research work is innovative. The experimental results largely support the conclusions drawn. However, prior to formal publication, a few issues require attention and revision.

Response #2: We thank the reviewer #2 for his/her highly positive and constructive comments. We also appreciate very comprehensive suggestions to further improve the quality of our work.

Comment 2.1.1: *In the introduction, the author emphasizes the impacts of waste plastics, seemingly overemphasizing the significance of waste recycling while neglecting the importance of the sensing material itself. The logic and rigor in developing the PW_{12}-PET sensing material are lacking.*

Response 2.1.1: We sincerely thank the reviewer for this insightful comment. In response to this suggestion, we have streamlined the discussion regarding the impact of waste plastics. Furthermore, we have substantially revised the Introduction to clarify the rational design of the PW_{12}-PET sensing material. The logic is now strengthened based on the following two aspects:

1. The molecular-level tunability of polyoxometalates (POMs) enables them to function as both interfacial activators and sensing centers. Specifically, their robust **Brønsted acidity** facilitates **the proton-mediated activation of functional groups on otherwise inert plastic surfaces**[13]. This process ensures stable chemical anchoring and molecular-level functionalization through a synergistic network of hydrogen bonding and electrostatic interactions.

2. The specific functional groups (such as esters, phenyl groups, amides, or halogens) introduced by the plastic effectively **modulate the surface electronic environment of the POM active sites**[14,15]. Specifically, the functional groups provided by the plastic (such as esters, phenyl groups, amides, or halogens) **effectively modulate the surface electronic environment of the POM active sites**, leading to enhanced sensing performance.

Accordingly, we have revised the Introduction and the corresponding changes are highlighted in yellow: "In this context, polyoxometalates (POMs) provide a unique solution to this challenge. Specifically, their molecular-level tunability enables them to function as both interfacial activators and sensing centers. Their strong Brønsted acidity and low corrosivity enable the selective activation of specific functional groups (such as C=O in esters) on inert plastic surfaces, facilitating stable chemical anchoring

through a synergistic network of hydrogen bonding and electrostatic interactions. Furthermore, the inherent ability of POM anions to undergo reversible multi-electron transfer without structural degradation significantly facilitates charge transfer during gas interaction. Crucially, the plastic functions as a functionalized platform that modulates the electronic environment and charge kinetics of the POM active sites. This structure effectively addresses the inherent limitations of pure POMs (limited active site exposure), thereby transforming waste plastics into high-performance, active components for gas sensing." And "The significance of this composite proposing a synergistic mechanism rather than simple blending: ball-milling induces charge transfer from PET to PW_{12}, thereby optimizing the surface electronic structure and boosting sensitivity. This work provides a green, economical strategy for upcycling waste plastics into high-performance functional materials, offering a new perspective for sustainable waste management."

Comment 2.1.2: *Additionally, the author mentions "The optimized PW_{12}-PET-1 sample achieves a low limit of detection (LOD) 54 of 2.76 ppb, along with fast response and recovery times of 19.2 s and 13.5 s at 5.0 ppm NO_2, respectively. It outperforms reported NO_2 sensors." The author's introduction presents a one-sided view of the sensor's performance. Can these few points alone demonstrate superiority over the reported NO_2 sensors? For gas sensors, characteristics such as response values, moisture resistance and good repeatability are all factors that need to be considered.*

Response 2.1.2: Many thanks for the reviewer's constructive comment. Accordingly, **we have included experimental data on response values, moisture resistance, and repeatability** in the revised Manuscript and Table R2 (Supplementary Table 2 in Supplementary Information).

1. **Response values**: We acknowledge that while the response value of our sensor is within a competitive range rather than being the highest, its ability to detect ppm-level NO_2 at room temperature within seconds is a significant advantage. For practical real-time monitoring (especially for toxic gases like NO_2), the rapid response within seconds is often more critical than a high absolute value. This allows for immediate early warning, which provides a significant advantage.

2. **Moisture resistance**: We acknowledge the current limitation under high-humidity conditions (>54% RH). However, this operational window effectively covers indoor air quality monitoring and dry industrial process control, meeting the requirements for many sensing environments.

3. **Repeatability**: The long-term cyclic stability test over 21 days demonstrates its satisfactory repeatability. Achieving such reliable stability using waste plastic as a matrix is a significant breakthrough, showing the potential for transforming waste

polymers into dependable functional materials.

While certain reported sensors may exhibit superior performance in specific parameters, the primary contribution of this work aims to balance high-value upcycling with performance. We have revised the statement in the Introduction from "It outperforms reported NO_2 sensors." to "It demonstrates an impressive combination of rapid response/recovery time and LOD."

Table R2 | Performance Comparison with other Chemoreceptive Materials. Comparison of the sensing performance of various materials toward NO_2 at room temperature (25 °C), as reported in the literature

Materials	Auxiliary method	t_{res}/t_{rec} (s)	LOD (ppb)	Res. %/ Con. (ppm)	Humidity (%)	Repeatability (day)	Ref.
Au-CeO_2	photo-thermal-activated	17/15	15	20/10	5–70	18	[3]
Au-$Bi_xIn_{2-x}O_3$	light-activated	348/300	100	64.9/0.4	0–50	–	[4]
p–n MoS_2	UV radiation	150/30	8	50/1	–	180	[5]
TiO_2@NH_2-MIL-125	520 nm light	16.8/78	1	207/100	–	365	[6]
HIOTP-Ni	–	101.4/619.2	210	405/10	–	–	[7]
$Mo_2TiC_2T_x$/MoS_2	–	34.8/140.5	2.5	415.8/50	0–90	14	[8]
VO_x/LIG	–	217/650	0.45	2.5/1	50–80	16	[9]
CdS QDs	–	29/28	11	17.6/1.76	50	–	[10]
Al-ZnO	–	360/180	200	1.4/0.5	10–75	60	[11]
C-MoS_2	–	43.1/301.2	130	2500/10	–	–	[12]
**PW_{12}-PET-1**	–	**23.2/1.8**	**10.52**	**2588/100**	**10–54**	**21**	**This work**
	–	**19.2/13.5**		**30/5.0**			

Comment 2.2: *As mentioned above, the role of waste plastics appears to be overstated. The author should focus on elaborating the significance of using plastics in combination with polyoxometalates as sensing materials. After all, polyoxometalates have been widely applied as gas sensing materials.*

Response 2.2: We thank the reviewer for carefully reading of our work. We have substantially revised the Introduction to clarify the role of waste plastics and the significance of the PW_{12}-PET sensing material.

(1) This strategy provides an innovative strategy for the **upcycling of waste plastics towards high-value-added functional materials**. Unlike traditional chemical depolymerization, which requires harsh conditions (high temperatures or corrosive reagents), our approach retains the polymer backbone as a functional scaffold, achieving a high degree of economy and environmental benefits.

(2) The molecular-level tunability of polyoxometalates (POMs) enables them to function as both **interfacial activators and sensing centers**. Specifically, their robust **Brønsted acidity** facilitates the **proton-mediated activation of functional groups on otherwise inert plastic surfaces**[13]. This process ensures **stable chemical anchoring and molecular-level functionalization** through a synergistic network of hydrogen bonding and electrostatic interactions.

(3) The specific functional groups (such as esters, phenyl groups, amides, or halogens) introduced by the plastic effectively **modulate the surface electronic environment of the POM active sites**[14,15]. Specifically, the functional groups provided by the plastic (such as esters, phenyl groups, amides, or halogens) effectively **modulate the surface electronic environment of the POM active sites**, leading to enhanced sensing performance.

We have revised the Introduction to clearly state the significance of using plastics in combination with polyoxometalates as a sensing material: "In this context, polyoxometalates (POMs) provide a unique solution to this challenge. Specifically, their molecular-level tunability enables them to function as both interfacial activators and sensing centers. Their strong Brønsted acidity and low corrosivity enable the selective activation of specific functional groups (such as C=O in esters) on inert plastic surfaces, facilitating stable chemical anchoring through a synergistic network of hydrogen bonding and electrostatic interactions. Furthermore, the inherent ability of POM anions to undergo reversible multi-electron transfer without structural degradation significantly facilitates charge transfer during gas interaction. Crucially, the plastic functions as a functionalized platform that modulates the electronic environment and charge kinetics of the POM active sites. This structure effectively addresses the inherent limitations of pure POMs (limited active site exposure), thereby transforming waste plastics into high-performance, active components for gas sensing." And "The significance of this composite proposing a synergistic mechanism rather than simple blending: ball-milling induces charge transfer from PET to PW_{12}, thereby optimizing the surface electronic structure and boosting sensitivity. This work provides a green, economical strategy for upcycling waste plastics into high-performance functional materials, offering a new perspective for sustainable waste management."

Comment 2.3: *Regarding the microstructure of the composite materials, the manuscript provides only one TEM image, and the authors' stated findings cannot be clearly discerned from it. Additional evidence is required.*

Response 2.3: Thanks for the reviewer's comments. To clearly distinguish the PW_{12} nanoparticles within the PW_{12}-PET-1, **we have provided additional Cryo-Transmission Electron Microscopy (Cryo-TEM) images**. As shown in Fig. R7a, the PET surface is modified with many nanoparticles exhibiting different contrast, representing PW_{12} clusters. The magnified view in Fig. R7b further confirms that these clusters are dispersed with **an average diameter of approximately 2.5 nm**. This small particle size is benefit for exposing more active sites for gas sensing. However, under further higher magnification, the PET is highly beam-sensitive and undergoes localized radiolytic damage and thermal degradation. This leads to morphological instability and deterioration of the image focus, which prevents the acquisition of higher-resolution image.

These additions have been incorporated into the revised Manuscript and the Methods: "As shown in Fig. 2a, the Cryo-Transmission Electron Microscopy (Cryo-TEM) image reveals that PW_{12} nanoparticles are well dispersed on the PET matrix. After magnifying the selected region highlighted by the red circle in Fig. 2a, the average particle size was statistically determined to be approximately 2.5 nm (Fig. 2b)." and "Cryo-Transmission Electron Microscopy (Cryo-TEM) images were carried out using a field-emission transmission electron microscope (Talos F200C, FEI) with 200 keV accelerating voltage."

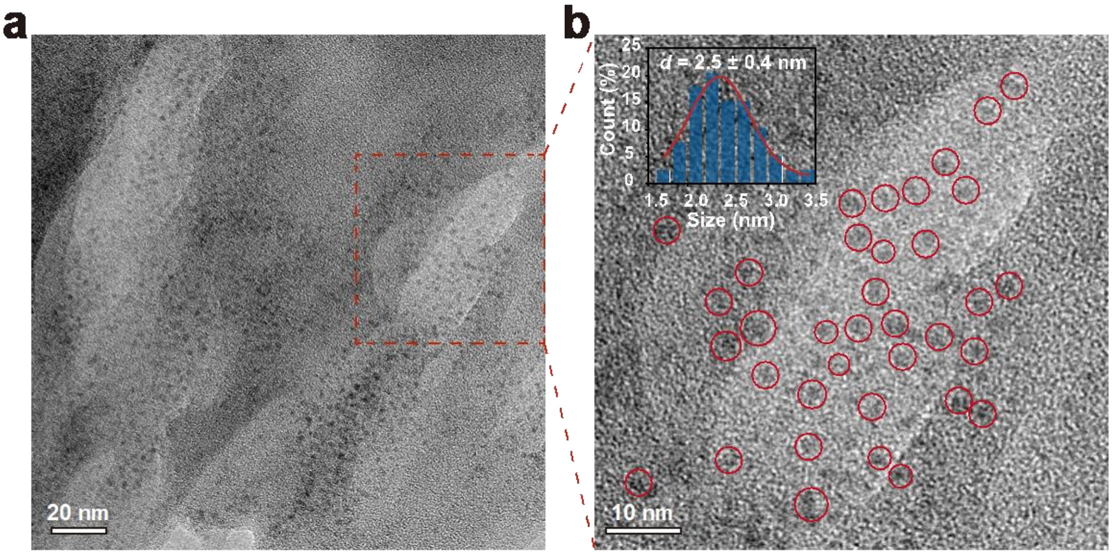

Fig. R7 | Cryo-TEM characterization of PW_{12}-PET-1. a. Cryo-TEM image and **b.** corresponding magnified image of PW_{12}-PET-1. The red circle identifies a region of high contrast assigned to PW_{12} particles. The particle size distribution plot in the upper left corner corresponds to PW_{12} in PW_{12}-PET-1, with an average diameter of approximately 2.5 nm.

Comment 2.4: *The theoretical analysis in the manuscript are detailed and thorough, and PDOS is provided as supplementary material. However, the gas sensing test section is relatively weak. In Figure 3e, the sensor's resistance in* NO_2 *did not reach a*

stable value.

Response 2.4: We thank the reviewer for the specific comment. To strengthen the gas sensing test, we have conducted additional **humidity-dependent and temperature-dependent sensing tests**. Furthermore, **diffuse reflectance infrared Fourier transform spectra (DRIFTS) and in-situ Raman measurements** were performed to further investigate the sensing mechanism.

The discussion regarding humidity-dependent performance is mentioned in Response 2.5. Furthermore, additional investigations concerning **temperature-dependent performance, as well as in-situ Raman and DRIFTS results**, have been added to the Manuscript: "As shown in Fig. 3g and Supplementary Fig. 7a, the temperature-dependent response exhibits a volcano-shaped profile, peaking at 100 °C. The initial increase is attributed to the accelerated thermal motion and improved charge transfer, whereas the subsequent decline results from the loss of crystalline water in PW_{12} and thermal deterioration of the PET substrate." and "To understand the dynamic surface reactions and charge transfer processes of PW_{12}-PET-1 during NO_2 sensing, in-situ diffuse reflectance infrared Fourier transform spectra (DRIFTS) and in-situ Raman measurements were performed. As shown in Fig.4a, upon exposure to NO_2, DRIFTS reveals that NO_2 is rapidly adsorbed on the PW_{12}-PET-1 surface, as evidenced by the characteristic NO_2 band observed at 1621 cm^{-1}. Following adsorption, NO_2 accepts electrons from the sensor and reacts with the protons around adsorption sites to form NO and H_2O, as indicated by the appearance of peaks at 1894 cm^{-1} and 3582 cm^{-1}, respectively[47–49]. Moreover, a strong signal at 2033 cm^{-1}, attributed to the $NO^{\delta+}$ intermediate appears[50], reflecting a strong interaction between NO and PW_{12}. Concurrently, in-situ Raman spectra (Fig. 4b) show that the W–O–W bridge-oxygen peak gradually shifts to higher wavenumbers during NO_2 adsorption, providing direct evidence for electron transfer from PW_{12}-PET-1 to NO_2. Thus, the corresponding adsorption pathways can be summarized as follows:

$$NO_2+2e^-+2H^+ \rightarrow NO+H_2O \quad (1)$$

$$NO+PW_{12} \rightarrow [PW_{12}^{\delta-}\text{-}NO^{\delta+}] \quad (2)$$

Subsequently, the reversibility of PW_{12}-PET-1 was confirmed during the desorption process. Upon switching to air, the NO_2 adsorption peak and the intermediate signals in DRIFTS difference spectra rapidly declined, while the W–O–W peak in Raman returned to its initial position. This indicates that the adsorption-desorption reaction is

highly reversible, with NO and H_2O undergoing an inverse reaction to regenerate NO_2 and H^+, thereby restoring the initial electronic state.

$$[PW_{12}^{\delta-}\text{-}NO^{\delta+}] \rightarrow NO+PW_{12} \quad (3)$$

$$NO+H_2O \rightarrow NO_2+2e^-+2H^+ \quad (4)$$"

Furthermore, to address the concern regarding stability, we retested the continuous cycle stability of PW_{12}-PET-1 under 5.0 ppm NO_2 at room temperature. **During five consecutive cycles, the sensor consistently showed a response of around 30% and successfully returned to its baseline after each cycle (Fig. R8)**, demonstrating the excellent stability and reproducibility of the sensor.

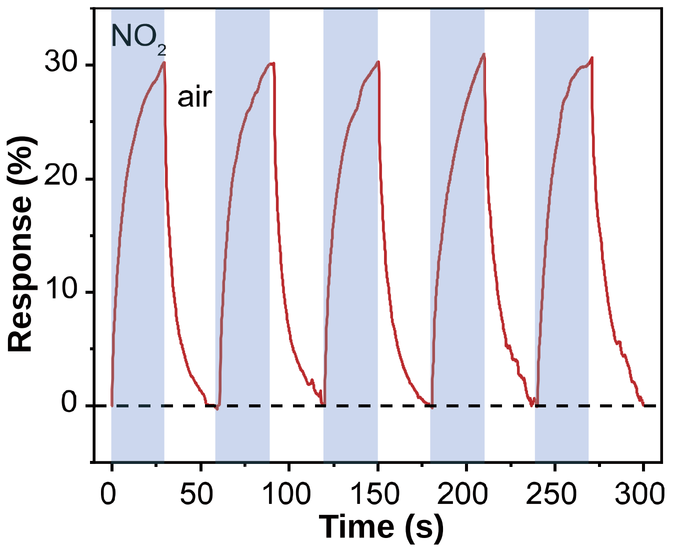

Fig. R8 | Five consecutive response-recovery cycles of PW_{12}-PET-1 at 5.0 ppm NO_2. The dotted line indicates a response value of 0%.

Comment 2.5: *For NO_2 gas detection, humidity testing is crucial. Supplement the sensor with test curves for NO_2 at different humidity levels.*

Response 2.5: Thanks for the reviewer's constructive suggestion. In accordance with the reviewer's recommendation, we conducted additional humidity-dependent sensing measurements at a fixed NO_2 concentration of 5.0 ppm, spanning a **relative humidity (RH) range of 10%–98%**.

As shown in Fig. R5a, within the 10%–98% RH range, the sensing response of PW_{12}-PET-1 first increases and then decreases, **reaching a maximum of approximately 40% at 34% RH**. Since our laboratory humidity generally ranges between 10% and 40% RH, the previously measured NO_2 response (30%) at ambient humidity falls within this range, confirming the reproducibility and reliability of the humidity-dependent results.

The observed humidity-dependent behavior can be explained as follows: Below 34% RH, increasing humidity facilitates charge transport within the PW_{12}-PET-1 composite, thereby enhancing the sensing response[7]. In contrast, when the humidity exceeds this threshold, excessive water molecules may competitively occupy the active adsorption sites, especially on the oxygen-rich surfaces of PW_{12}, which hinders NO_2 adsorption and reduces the sensing signal. Once the humidity exceeds 54% RH, the detection process becomes irreversible because the desorption of the analyte during the recovery process becomes extremely slow[8]. Thus, the current cannot return to its baseline after the recovery step. At extreme humidity (98% RH), the formation of a continuous water film on the surface leads to a short circuit (Fig. R5b), resulting in functional failure. This phenomenon is commonly observed in many analogous materials[9–12].

Following these discussions, the corresponding content has been added to the Manuscript and the Methods: "Similarly, the effect of relative humidity was also assessed (Fig. 3h and Supplementary Fig. 7b), with a peak response at 35% RH, as moderate humidity facilitates charge transport. Beyond this range, increased humidity slows recovery, and near 100% RH, short-circuiting may occur due to surface water film formation." and "Correspondingly, the relative humidity (RH) was independently adjusted (10%, 34%, 54%, 76%, and 98%) under a constant 5.0 ppm NO_2."

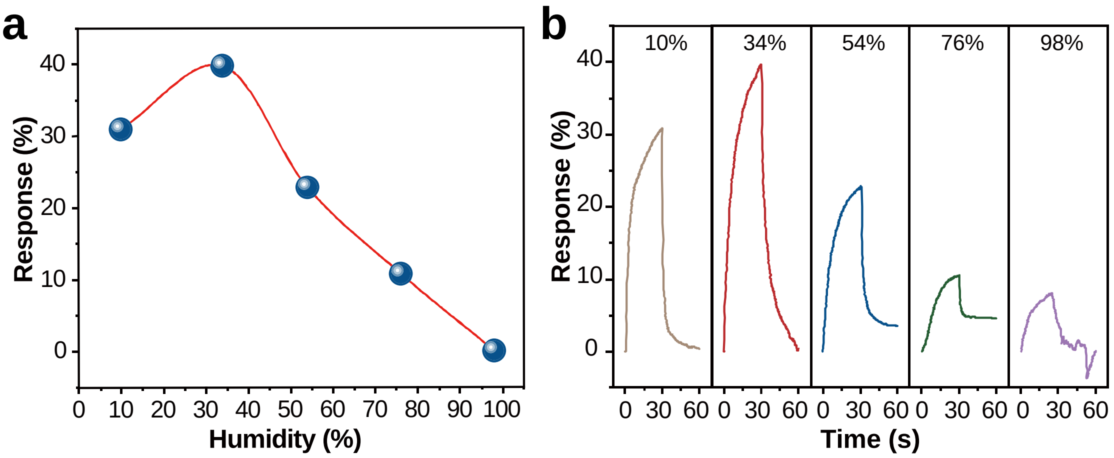

Fig. R5 | Humidity-dependent sensing performance of PW_{12}-PET-1 toward 5.0 ppm NO_2. **a**, Gas-sensing response and **b**, normalized response-recovery curves at various relative humidity (RH) levels (10%, 34%, 54%, 76%, and 98%).

Reviewer #3: (Remarks to the authors)

The authors demonstrate a mechanochemically synthesized sensor for NO_2 detection, which exhibits a relatively low limit of detection (LOD) and a fast response rate. Upon addressing the following points, the manuscript would be suitable for publication in Nature Communications:

Response #3: We thank the reviewer #3 for his/her opinion on our work. We also appreciate the reviewer's approval on our experimental section.

Comment 3.1: *In lines 67–69, the authors state: "Water not only promotes complete dissolution of PW_{12}, facilitating proton activation and molecular-level functionalization of PET by polyoxoanions, but also increases lubricity and prevents the fracture of polymer chains." This claim should be supported by a relevant reference to strengthen its validity.*

Response 3.1: Thank you for the suggestion. The previous explanation regarding the role of water was unscientific.

To clarify the role of water in the ball-milling process, we supplemented a control experiment without the addition of water during ball milling (PW_{12}-PET-without water, Fig. R4a). As shown in Figs. R4b,c, it can be observed that, **in the absence of water, the samples are agglomerated and sticked to the bottom of the ball-mill jar due to mechanical forces**. Conversely, the introduction of water during ball milling effectively prevented these issues (Figs. R4d–4f).

Subsequently, X-ray diffraction (XRD) analysis (Fig. R4g) reveals that PW_{12} maintains its crystalline structure, while PET exhibits a weak and broad diffraction peak owing to the low crystallinity of pristine PET, consistent with the PW_{12}-PET-1. Fourier-transform infrared (FTIR) spectroscopic analysis (Fig. R4h) reveals that only a small fraction of PET in PW_{12}-PET-without water is effectively functionalized by PW_{12}. This is evidenced by the slight blue shifts of several characteristic vibrational bands of PET (C=O, C–O–C, and $–CH_2$), whereas the majority of the characteristic peaks still remain unchanged compared with those of pristine PET and PW_{12}. **These results demonstrate that PET cannot be effectively functionalized by PW_{12} in the absence of water.**

Therefore, water plays a crucial role in the molecular-level functionalization of PET by PW_{12}, as summarized below:

(1) It preventing agglomeration during ball milling;

(2) It promotes the complete dissolution of PW_{12};

(3) It enables proton activation and molecular-level functionalization of PET by PW_{12}.

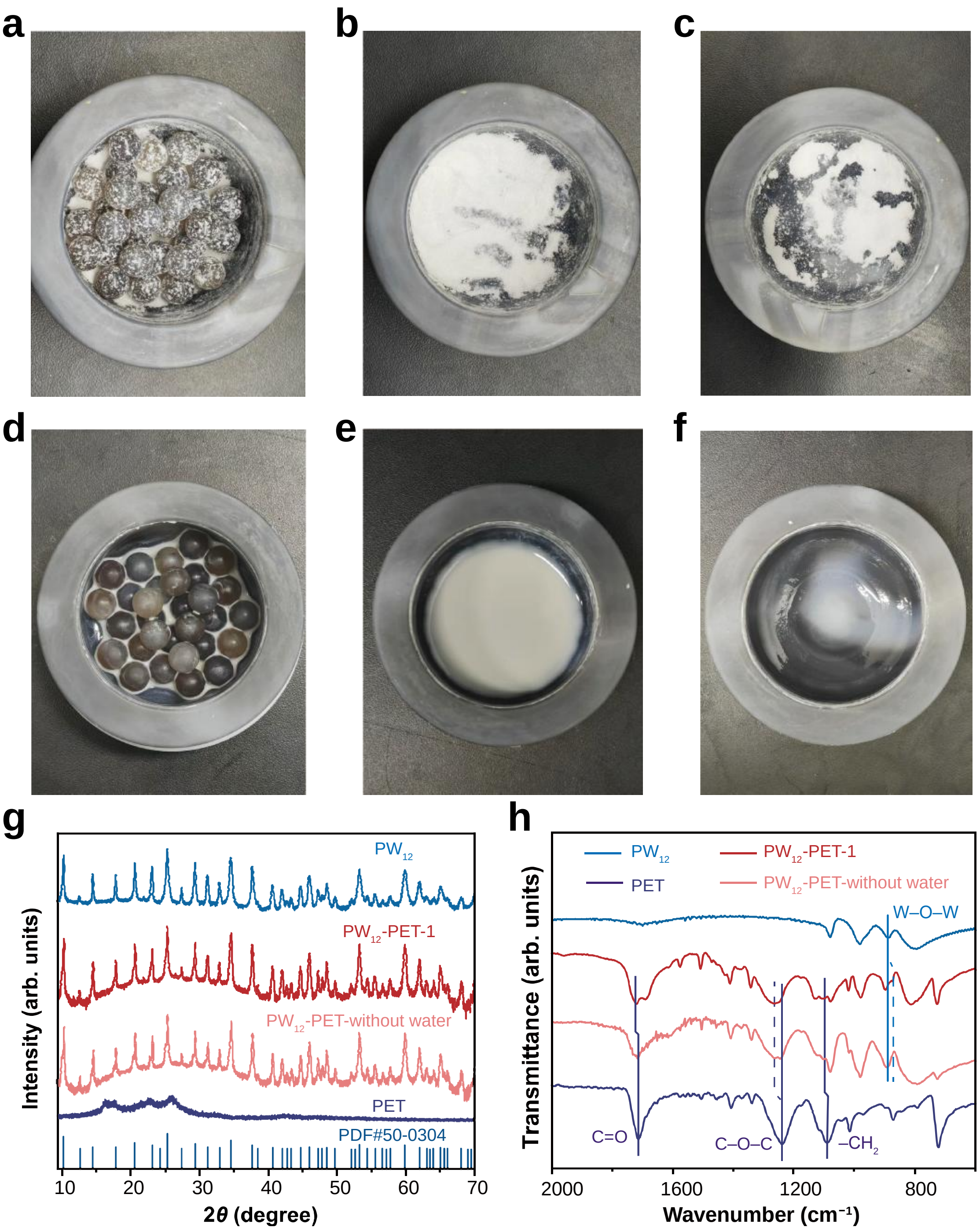

Fig. R4 | Comparison of samples under different ball-milling conditions. a–c, Optical images after ball milling without water. Part of the sample adheres to the surface of the agate ball (**a**). The sample condition after removing the agate ball shows that the most of the material remains in powder form (**b**), regions exhibit an agglomerated phenomenon (**c**). This compaction is speculated to be related to the crystalline water in PW_{12}. **d–f,** Optical images after ball milling with water, where no agglomeration was observed. **g**, XRD patterns of PW_{12}, PET, PW_{12}-PET-1 and PW_{12}-PET-without water. PW_{12}-PET-without water consisted of the amorphous PET phase with broad peaks and crystalline PW_{12} peaks (PDF#50-0304 corresponds to $PW_{12}\cdot 6H_2O$). **h**, FTIR spectra of PW_{12}, PET, PW_{12}-PET-1 and PW_{12}-PET-1 without water.

In mechanochemical processes, small amounts of liquids (such as H_2O) are commonly added as lubricants[5,6]. However, in our ball-milling system operating at a low transmission ratio (1:−1) and a low rotation speed of 300 rpm, lubricity does not represent the primary role of water.

Accordingly, the description of the function of water in the Manuscript has been revised as follows: "In this process, water plays a crucial role. It prevents particle agglomeration and promotes complete dissolution of PW_{12}, thereby facilitating effective proton activation and molecular-level functionalization of PET by polyoxoanions."

Comment 3.2: *The field of view in Figure 2a is too large to clearly distinguish the PW_{12} nanoparticles. It is recommended that the authors either magnify the image or include an inset showing a higher-magnification region to better visualize the PW_{12} particles.*

Response 3.2: Thanks for the reviewer's comments. To clearly distinguish the PW_{12} nanoparticles within the PW_{12}-PET-1, we have provided additional Cryo-Transmission Electron Microscopy (Cryo-TEM) images. As shown in Fig. R7a, the PET surface is modified with many nanoparticles exhibiting different contrast, representing PW_{12} clusters. The magnified view in Fig. R7b further confirms that these clusters are dispersed with **an average diameter of approximately 2.5 nm**. This small particle size is crucial for maintaining a high active surface area for gas sensing. However, during imaging under higher magnification, the PET is highly beam-sensitive, undergoing localized radiolytic damage and thermal degradation. This leads to morphological instability and deterioration of the image focus, which prevents the acquisition of higher-resolution image.

These additions have been incorporated into the revised Manuscript and the Methods: "As shown in Fig. 2a, the Cryo-Transmission Electron Microscopy (Cryo-TEM) image reveals that PW_{12} nanoparticles are well dispersed on the PET matrix. After magnifying the selected region highlighted by the red circle in Fig. 2a, the average particle size was statistically determined to be approximately 2.5 nm (Fig. 2b)." and " Cryo-Transmission Electron Microscopy (Cryo-TEM) images were carried out using a field-emission transmission electron microscope (Talos F200C, FEI) with 200 keV accelerating voltage."

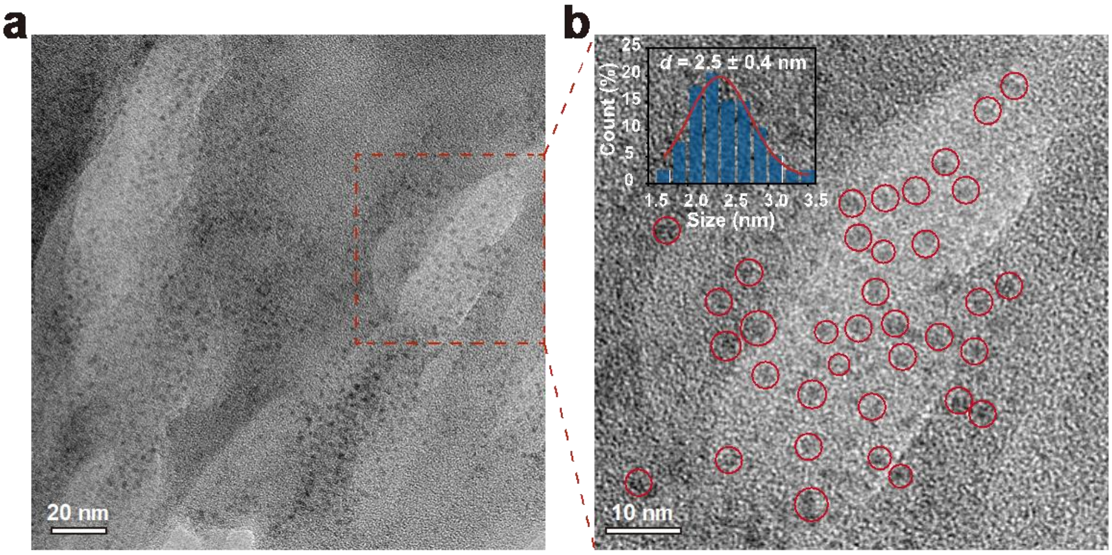

Fig. R7 | Cryo-TEM characterization of PW_{12}-PET-1. a. Cryo-TEM image and **b.** corresponding magnified image of PW_{12}-PET-1. The red circle identifies a region of high contrast assigned to PW_{12} particles. The particle size distribution plot in the upper left corner corresponds to PW_{12} in PW_{12}-PET-1, with an average diameter of approximately 2.5 nm.

Comment 3.3: *Regarding the O 1s XPS spectra in Fig. 2d, the signals for both PW_{12} and PW_{12}-PET-1 appear as a broad envelope at approximately 534-536 eV. The deconvolution into three, or even five, closely spaced peaks and the subsequent reliance on the subtle shifts of these deconvoluted peaks to demonstrate charge transfer lack sufficient rigor. The presented O 1s data may not be robust evidence. It might be preferable to remove Fig. 2d, as the C 1s spectra in Fig. 2e, which show more distinct peak shifts, provide adequate and clearer XPS evidence for the charge transfer.*

Response 3.3: Thank you for your valuable comment**.** We agree with the reviewer's comments that the broad O 1s envelope and the subsequent deconvolution lack the necessary rigor to serve as robust evidence for charge transfer. Following your suggestion, we have removed Fig. 2d (O 1s spectra) and the related discussion from the manuscript. Corresponding revisions have been made throughout the text to ensure consistency and coherence.

Comment 3.4: *In lines 263–264, the authors note that all measurements were conducted under laboratory conditions at 25 °C. It would be important to evaluate the influence of temperature and humidity on the NO_2 sensing performance of PW_{12}-PET to assess its practical applicability.*

Response 3.4: Thanks for the reviewer's constructive suggestion. Following the reviewers' suggestions, we conducted additional **temperature-dependent and humidity-dependent sensing tests at a fixed NO_2 concentration of 5.0 ppm.** The experiment employed the single-factor variable method: keeping all other experimental conditions constant, the temperature was independently adjusted within the range of **25 to 200 °C**, and the relative humidity (RH) was independently controlled within the range of **10% to 98%**.

As shown in Fig. R9, the sensing response of **PW_{12}-PET-1 exhibits a typical temperature-dependent trend between 25 and 200 °C**, **reaching a maximum of approximately 113% at 100 °C**. The initial response enhancement (25–100 °C) is primarily driven by intensified thermal motion and reduced diffusion resistance, which facilitate the rapid transport of NO_2 molecules to active sites[16]. Additionally, rising temperatures accelerate the charge transfer kinetics between NO_2 and the PW_{12}-PET-1 surface. However, beyond 100 °C, the response begins to declines. This degradation is primarily ascribed to the loss of crystalline water within the PW_{12}[17], which reduces proton conductivity and counteracts the thermal promotion effect. Furthermore, as temperatures exceed 120 °C, the PET substrate begins to undergo thermal deterioration[18], further leading to a decline in sensing performance. Collectively, these detrimental effects counteract the benefits of thermal activation, leading to the observed decrease in response at higher temperatures.

As shown in Fig. R5a, within the 10%–98% RH range, the sensing response of PW_{12}-PET-1 first increases and then decreases, **reaching a maximum of approximately 40% at 34% RH**. Since our laboratory humidity generally ranges between 10% and 40% RH, the previously measured NO_2 response (30%) at ambient humidity falls within this range, confirming the reproducibility and reliability of the humidity-dependent results.

The observed humidity-dependent behavior can be explained as follows: Below 34% RH, increasing humidity facilitates charge transport within the PW_{12}-PET-1 composite, thereby enhancing the sensing response[7]. In contrast, when the humidity exceeds this threshold, excessive water molecules may competitively occupy the active adsorption sites, especially on the oxygen-rich surfaces of PW_{12}, which hinders NO_2 adsorption and reduces the sensing signal. Once the humidity exceeds 54% RH, the detection process becomes irreversible because the desorption of the analyte during the recovery process becomes extremely slow[8]. Thus, the current cannot return to its baseline after the recovery step. At extreme humidity (98% RH), the formation of a continuous water film on the surface leads to a short circuit (Fig. R5b), resulting in functional failure. This phenomenon is commonly observed in many analogous materials[9–12].

Following these discussions, the corresponding content has been added to the Manuscript and the Methods: "To further examine environmental adaptability, the response of PW_{12}-PET-1 under varying temperature and relative humidity (RH) conditions was investigated. As shown in Fig. 3g and Supplementary Fig. 7a, the temperature-dependent response exhibits a volcano-shaped profile, peaking at 100 °C. The initial increase is attributed to the accelerated thermal motion and improved charge transfer, whereas the subsequent decline results from the loss of crystalline water in PW_{12} and thermal deterioration of the PET substrate. Similarly, the effect of relative humidity was also assessed (Fig. 3h and Supplementary Fig. 7b), with a peak response at 35% RH, as moderate humidity facilitates charge transport[45]. Beyond this range, increased humidity slows recovery, and near 100% RH, short-circuiting may occur due to surface water film formation." and "The temperature-dependent and humidity-dependent tests employed the single-factor variable method. While keeping other experimental conditions constant, the temperature was varied (25, 50, 100, 150 and 200 °C) during exposure to 5.0 ppm NO_2. Correspondingly, the relative humidity (RH) was independently adjusted (10%, 34%, 54%, 76%, and 98%) under a constant 5.0 ppm NO_2."

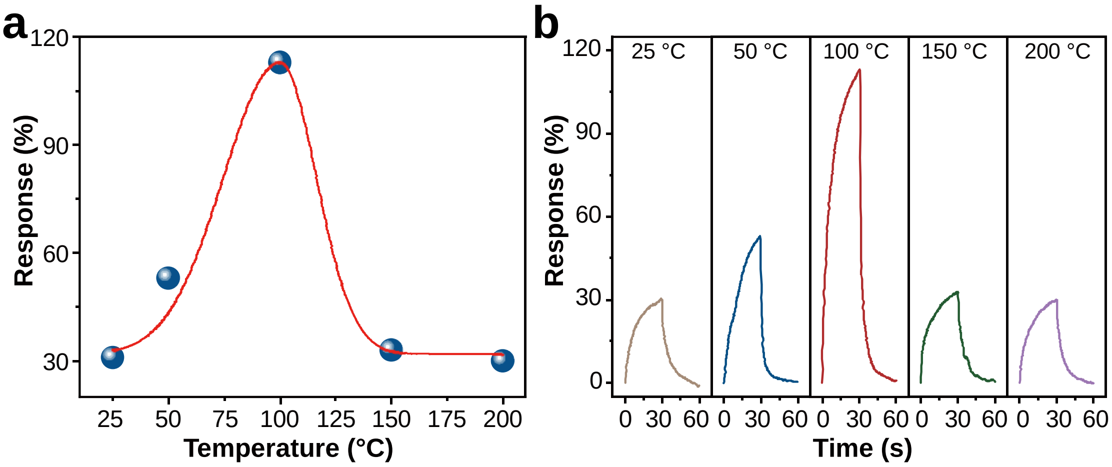

Fig. R9 | a, NO_2 gas-sensing response measurement and **b**, normalized response-recovery curve of PW_{12}-PET-1 under different temperature conditions (25, 50, 100, 150 and 200 °C) upon exposure to 5.0 ppm NO_2.

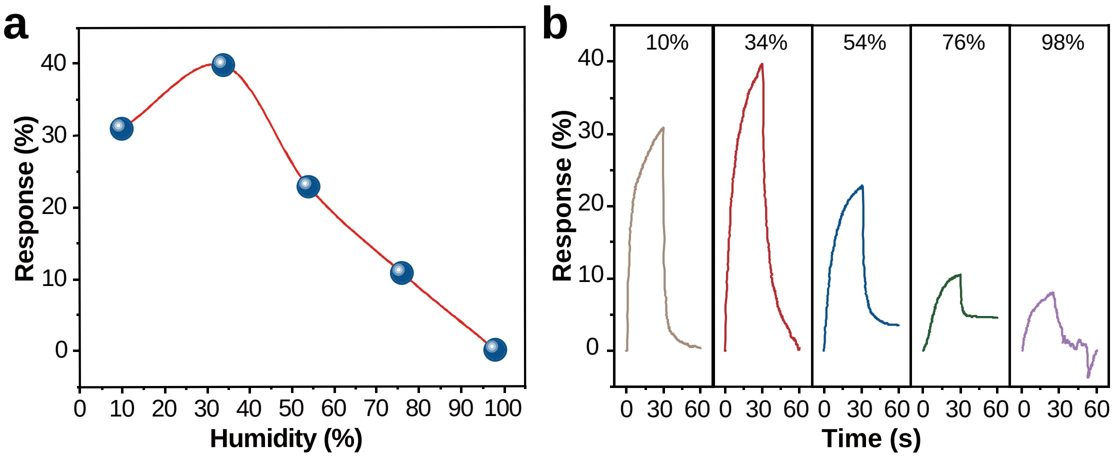

Fig. R5 | a, NO_2 gas-sensing response measurement and **b**, normalized response-recovery curve of PW_{12}-PET-1 under different relative humidity conditions (10%, 34%, 54%, 76%, and 98%) upon exposure to 5.0 ppm NO_2.

Comment 3.5: *Table 1 and Supplementary Table 2 largely present overlapping information. Merging them into a single comprehensive table would improve clarity and avoid redundancy.*

Response 3.5: Thanks for the reviewer's comments. We would like to clarify that **Table 1 and Supplementary Table 2 serve two distinct comparative purposes**. **Table 1 compares the performance of our material across different sensing methods** (such as optical and field-effect transistor (FET)-based sensors). In contrast, **Supplementary Table 2 provides a detailed comparison with other chemoreceptive materials**, specifically to complement the performance data shown in Fig. R2 (Fig. 3c in Manuscript). To avoid any confusion and clarify the distinction between these two tables, we have made the following revisions:

1. The caption for Fig. R2 (Fig. 3c in Manuscript) has been revised to: "Comparison of t_{res}, t_{rec} and limit of detection (LOD) of PW_{12}-PET-1 at 5.0 ppm with previously reported chemoreceptive materials."

2. The caption for Supplementary Table 2 has been updated to: "**Performance Comparison with other Chemoreceptive Materials.** Comparison of the sensing performance of various materials toward NO_2 at room temperature (25 °C), as reported in the literature"

3. The sequence of lines 138 has been adjusted to (Fig. 3c, Supplementary Table 2 and Table 1), and the position of Table 1 has been moved after Figure 3 to improve the narrative flow.

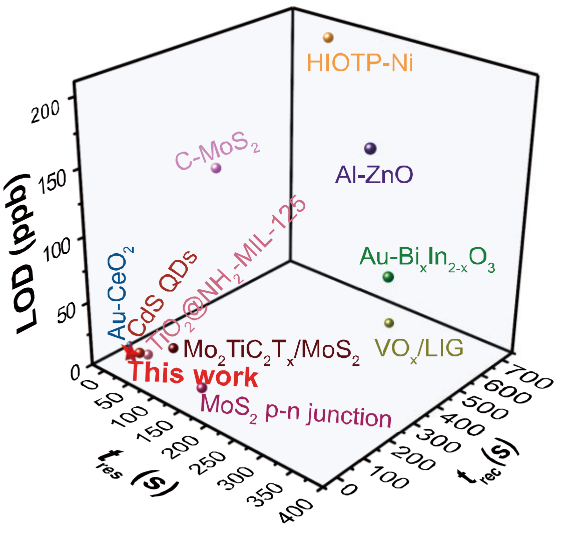

Fig. R2 | Comparison of t_{res}, t_{rec} and limit of detection (LOD) of PW_{12}-PET-1 at 5.0 ppm with previously reported chemoreceptive materials.

Comment 3.6: *While the study effectively demonstrates the principle of functionalization using reagent-grade PET, a more compelling case for the "upcycling of waste plastics" could be made by validating the method with real post-consumer waste. It is therefore recommended that the authors synthesize PW_{12}-PET from real-life PET waste like discarded bottles or fibers as sources and compare its sensing performance to the baseline material. This direct comparison is crucial for bridging the gap between a laboratory proof-of-concept and a genuine waste upcycling strategy.*

Response 3.6: We thank the reviewer for the valuable and constructive suggestion. In response, **we have conducted additional experiments using real post-consumer PET waste**. Four representative forms of PET waste—discarded bottles, packaging sheets, thermoformed plates, and textile fibers—were collected and subjected to the same mechanochemical functionalization procedure as applied to reagent-grade (virgin) PET. Then, their gas-sensing performance were tested and compared under 100 ppm NO_2.

As shown in Fig. R10, the sensing responses of PW_{12}-PET derived from discarded bottles, packaging sheets, thermoformed plates, and textile fibers were 2384%, 2273%, 2647%, and 2694%, respectively, which are comparable to the 2588% observed for reagent-grade PET. The minor variations are likely due to experimental uncertainties or the presence of small amounts of additives added in the PET consume; however, these differences do not affect the overall sensing performance. These results indicate that

our mechanochemical functionalization strategy is robust and readily applicable to real post-consumer PET waste.

The Manuscript and the Methods have been updated as follows: "Based on these findings, we further evaluated the universality of the mechanochemical PW_{12}-PET approach by preparing composites from a variety of real-life PET waste sources, including bottles, sheets, plates, and fibers. Under 100 ppm NO_2, all composites exhibited consistently high responses (Fig. 3i), demonstrating that this strategy is broadly applicable across different PET substrates." and "The PET waste (discarded bottles, packaging sheets, thermoformed plates, and textile fibers) followed the same procedure as that of virgin PET: grinding into fine powder using a batch mill, followed by sieving through a 100-mesh screen. Subsequently, the same ball-milling method used for the preparation of PW_{12}-PET was adopted, and the resulting products of four types of PW_{12}-PET were obtained after drying."

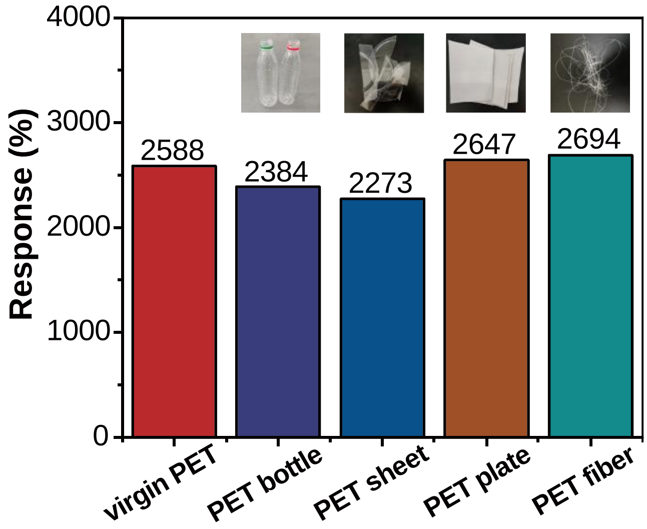

Fig. R10 | NO_2 Sensing Performance of PW_{12}-PET from various PET sources. Sensing responses of PW_{12}-PET derived from reagent-grade PET (virgin) and four types of post-consumer PET waste—including discarded bottles, packaging sheets, thermoformed plates, and textile fibers—under 100 ppm NO_2. All samples were prepared using the same mechanochemical functionalization procedure.

References

1. Cui, R., et al. The roles of brønsted acidity in low-temperature catalytic oxidation of NO over acidic zeolites with H_2O_2. *Chemosphere* 251, 126561 (2020).

2. Zhao, X., et al. Titanium nitride sensor for selective NO_2 detection. *Nat. Commun.* 16, 182 (2025).

3. Hu, J., et al. Dual-motor oxygen-deficiency-engineering al-doped $W_{18}O_{49-X}$ circular nanorod arrays for parts per billion-level acetone detection. *Nano Lett.* 25, 14131-14139 (2025).

4. Loiland, J.A.&Lobo, R.F. Oxidation of zeolite acid sites in NO/O_2 mixtures and the catalytic properties of the new site in no oxidation. *J. Catal.* 325, 68-78 (2015).

5. Braga, D., Maini, L.&Grepioni, F. Mechanochemical preparation of co-crystals. *Chem. Soc. Rev.* 42, 7638-7648 (2013).

6. Friščić, T., Trask, A.V., Jones, W.&Motherwell, W.D.S. Screening for inclusion compounds and systematic construction of three-component solids by liquid-assisted grinding. *Angew. Chem. Int. Ed.* 45, 7546-7550 (2006).

7. Min, H., et al. N-carbon-doped binary nanophase of metal oxide/metal–organic framework for extremely sensitive and selective gas response. *Adv. Mater.* 36, 2309041 (2024).

8. Park, S., et al. Dual-photosensitizer synergy empowers ambient light photoactivation of indium oxide for high-performance NO_2 sensing. *Adv. Mater.* 36, 2313731 (2024).

9. Niu, W., et al. Metal–organic framework-derived Ni-doped indium oxide nanorods for parts per billion-level nitrogen dioxide gas sensing at high humidity. *ACS Sens.* 9, 6103-6112 (2024).

10. Ou, Y., et al. Tandem electric-fields prolong energetic hot electrons lifetime for ultra-fast and stable NO_2 detection. *Adv. Mater.* 36, 2403215 (2024).

11. Kim, J.-O., et al. Large-area synthesis of nanoscopic catalyst-decorated conductive mof film using microfluidic-based solution shearing. *Nat. Commun.* 12, 4294 (2021).

12. Feng, Q., Huang, B.&Li, X. Graphene-based heterostructure composite sensing materials for detection of nitrogen-containing harmful gases. *Adv. Funct. Mater.* 31, 2104058 (2021).

13. Du, D.-Y., Qin, J.-S., Li, S.-L., Su, Z.-M.&Lan, Y.-Q. Recent advances in porous polyoxometalate-based metal–organic framework materials. *Chem. Soc. Rev.* 43, 4615-4632 (2014).

14. Cameron, J.M., et al. Supramolecular assemblies of organo-functionalised hybrid polyoxometalates: From functional building blocks to hierarchical nanomaterials. *Chem. Soc. Rev.* 51, 293-328 (2022).

15. Zhao, Y., et al. 3D covalent polyoxovanadate-organic framework as an anode for high-performance lithium-ion batteries. *Adv. Funct. Mater.* 33, 2306598 (2023).

16. Kim, S., et al. Aerosol-synthesized surfactant-free single-walled carbon nanotube-based NO_2 sensors: Unprecedentedly high sensitivity and fast recovery. *Adv. Mater.* 36, 2313830 (2024).

17. Ilbeygi, H., et al. Highly crystalline mesoporous phosphotungstic acid: A high-performance electrode material for energy-storage applications. *Angew. Chem. Int. Ed.* 58, 10849-10854 (2019).

18. Guo, Z., et al. Hydrogenating polyethylene terephthalate into degradable polyesters. *Angew. Chem. Int. Ed.* 64, e202418157 (2025).

Response to the Reviewers

Gao-Feng Han (On the behalf of all authors, who contributed to this work).

The additional comment regarding humidity from Reviewer 1 has been very helpful in further improving the quality of the manuscript. We sincerely thank the reviewer for the constructive suggestion and careful evaluation.

Reviewer #1: (Remarks to the authors)

The author provided detailed answers to the previous questions. However, some concerns remain unresolved.

Response #1: We sincerely thank the reviewer #1 for his/her highly positive and constructive comments. We also appreciate very comprehensive suggestions to further improve the quality of the work. In response to these comments, we have carefully revised the paper and provided detailed explanations and additional analyses as outlined below. We hope that the revised version will meet with the reviewer's approval.

Comment 1.1: *Although the impact of humidity on sensors has been extensively studied, this has been limited to the aspect of response. Authors should conduct a comprehensive study on the impact of humidity. For instance, the impact of different humidity levels on the baseline should be investigated. In the existing data, only changes after the injection of target gas are observed, and the baseline characteristics before the gas injection are missing. Changes in the baseline can affect the performance of sensor, such as cycle stability and long-term stability, etc.*

Response 1.1: Thanks for the reviewer's constructive suggestion. In response to this comment, **we have conducted a comprehensive investigation into the baseline and cyclic stability across a wide range of relative humidity (RH) levels (10%, 34%, 54%, 76%, and 98%).**

1. We have updated Fig. R1 to show the baseline before the introduction of the target NO_2 gas. Before exposure to NO_2, the prepared electrode was stabilized under each humidity condition for 1 h. The 10 s baseline resistance measured before gas introduction remains relatively stable, with negligible fluctuations when the RH is below 54%. This indicates that water adsorption at these humidity levels does not significantly influence the electronic properties of the sensing layer. In contrast, at 76% and 98% RH, the baseline shows noticeable fluctuations accompanied by a gradual upward drift. This behavior suggests a cumulative adsorption of water molecules on the PW_{12}-PET-1 surface, which may induce persistent changes in surface charge distribution and consequently lead to time-dependent baseline variation.

2. To evaluate the impact of humidity on the stability of PW_{12}-PET-1, we performed repetitive three cyclic tests (Fig.R2). The results reveal a clear humidity-dependent performance trend: Below 34% RH, the PW_{12}-PET-1 exhibits excellent baseline recovery and highly stable cyclic performance. The baseline remains nearly constant throughout the cycles, indicating that NO_2 adsorption at these levels is reversible. Once the humidity exceeds 54% RH, a slight baseline drift was observed. However, the PW_{12}-PET-1 still maintains over 85% of its initial response after three repetitive cyclic tests, demonstrating reasonable stability. The baseline drift becomes more pronounced at 76% RH, and the response magnitude decreases to approximately 60% after three repetitive cyclic tests. This decline is due to the fact that during the high-humidity recovery process, the water molecules hinder the desorption of NO_2, which remain on the surface and occupy the active sites for subsequent NO_2 adsorption. At extreme humidity (98% RH), the formation of a continuous water film on the surface leads to a short circuit (Fig. R1), thus making it impossible to conduct the cycle test.

Following these discussions, the corresponding content has been added to the Manuscript: "Beyond this range, increased humidity leads to time-dependent baseline drift and slower recovery, which is detrimental to the long-term stability and reusability of the sensor (Supplementary Fig. 8)[46]. When the relative humidity approaches 100%, short-circuiting may occur due to the formation of a surface water film."

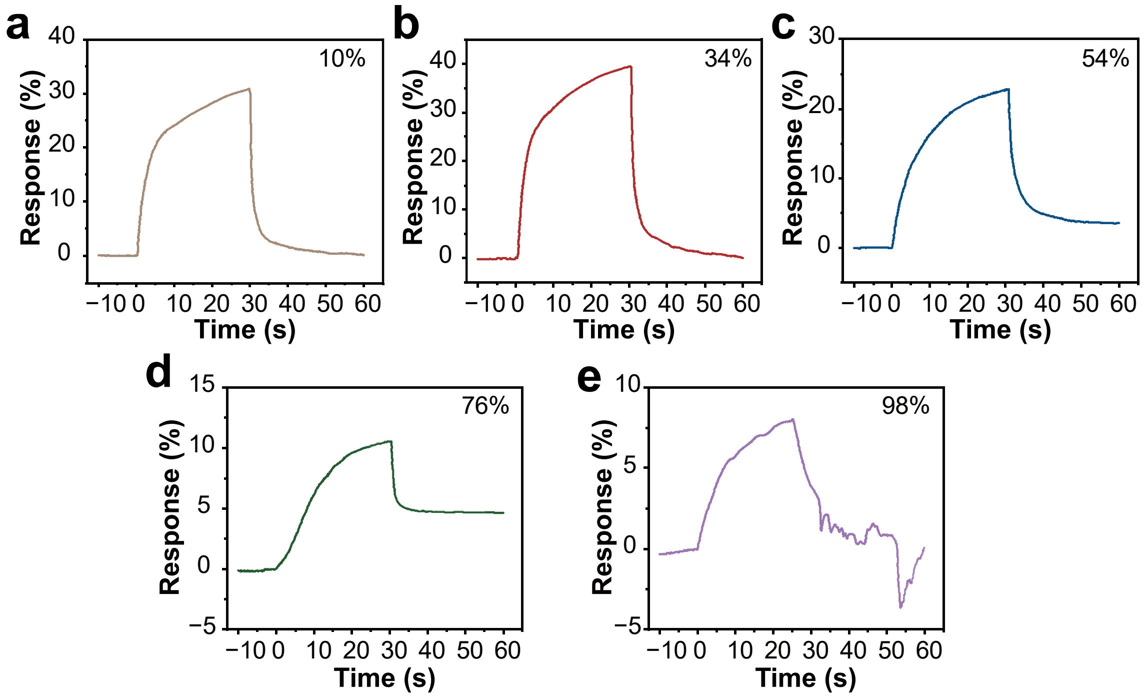

Fig. R1 | Normalized response-recovery curves of PW_{12}-PET-1 toward 5.0 ppm NO_2 under various relative humidity (RH) levels 10% (a), 34% (b), 54% (c), 76% (d), and 98% (e).

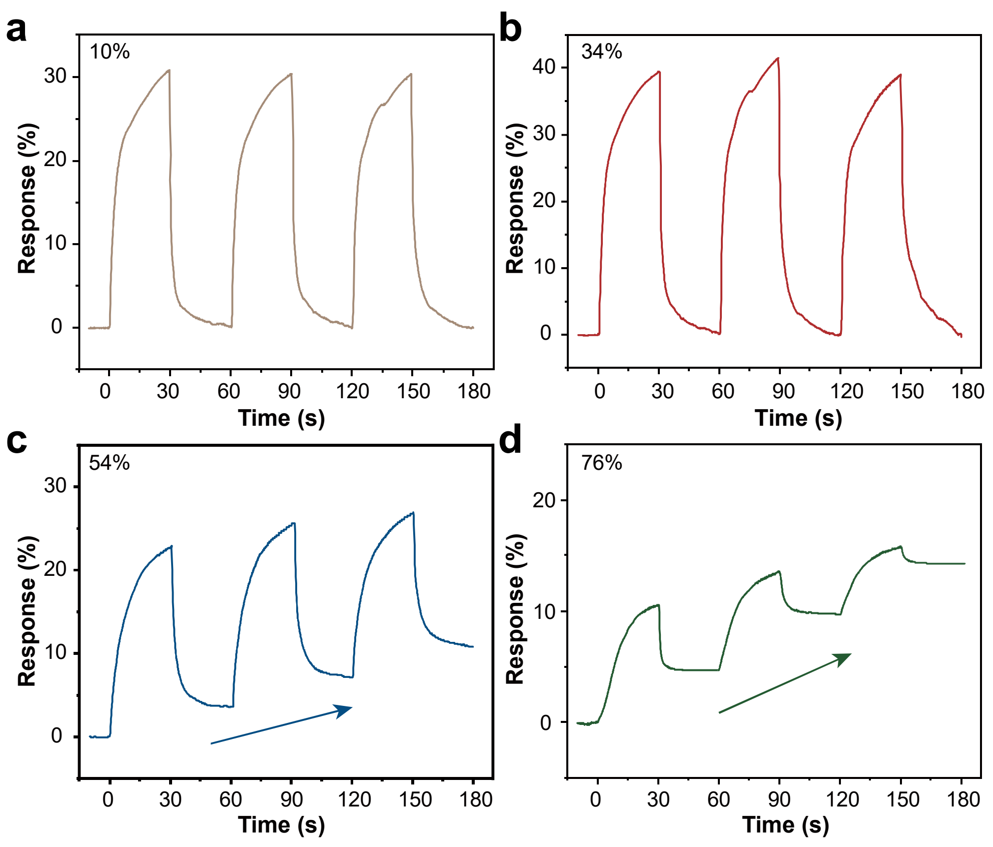

Fig. R2 | Repetitive response-recovery cycles of PW_{12}-PET-1 toward 5.0 ppm NO_2 at various relative humidity (RH) levels (10%, 34%, 54%, and 76%). The sensor exhibits high cyclic stability and baseline consistency at 10% RH (a) and 34% RH (b). Gradual baseline drift and decreased responses are observed at 54% RH (c) and 76% RH (d).

Reviewer #2: (Remarks to the authors)

The revised version is acceptable and the issues raised have been resolved.

Response #2: We thank the reviewer #2 for his/her highly positive comments. We also appreciate the reviewer's approval on our work.

Reviewer #3: (Remarks to the authors)

All reviewer comments have been clearly responded to, and the corresponding revisions have enhanced the quality of the manuscript. We consider the paper now ready for publication in Nature Communications in its present form.

Response #3: We thank the reviewer #3 for his/her opinion on our work. We also appreciate the reviewer's approval on our experimental section.
